# Robust perfluorophenylboronic acid-catalyzed stereoselective synthesis of 2,3-unsaturated *O*-, *C*-, *N*- and *S*-linked glycosides

**DOI:** 10.3762/bjoc.15.125

**Published:** 2019-06-11

**Authors:** Madhu Babu Tatina, Xia Mengxin, Rao Peilin, Zaher M A Judeh

**Affiliations:** 1 School of Chemical and Biomedical Engineering, Nanyang Technological University, Singapore, 62 Nanyang Drive, N1.2–B1-14, Singapore 637459, Tel.: +65-6790-6738; Fax: +65-6794-7553

**Keywords:** *C-*, *O-*, *N*- and *S*-linked glycosides, enosides, Ferrier-rearrangement, organocatalyst, pseudo-glycosides

## Abstract

A convenient protocol was developed for the synthesis of 2,3-unsaturated *C-, O-*, *N*- and *S*-linked glycosides (enosides) using 20 mol % perflurophenylboronic acid catalyst via Ferrier rearrangement. Using this protocol, D-glucals and L-rhamnals reacted with various *C-*, *O-, N*- and *S*-nucleophiles to give a wide range of glycosides in up to 98% yields with mainly α-anomeric selectivity. The perflurophenylboronic acid successfully catalyzed a wide range of substrates (both glucals and nucleophiles) under very mild reaction conditions.

## Introduction

2,3-Unsaturated glycosides, also known as pseudo-glycosides or enosides, are an important class of natural products with many biological activities and capacity to serve as substrates for further reactions [1–3]. They are involved in biochemical processes such as molecular recognition, cell–cell interaction, immunological recognition and transmission of biological information [4–6]. They are easily transformed into important bioactive compounds such as oligosaccharides, glycopeptides, nucleosides, antibiotics, uronic acids and other natural products [1–3].

The Ferrier rearrangement is one of the most useful processes to synthesize pseudo-glycosides in a direct and stereoselective fashion. Several classes of catalysts have been successfully applied in the Ferrier rearrangement including Brønsted acids [7–13], Lewis acids [14–19], redox reagents [20] and metal catalysts [21–23]. However, many of these catalysts have limited substrate scope, give variable selectivities and yields, require harsh reaction conditions and an excess amount of catalysts that are typically expensive, toxic and moisture/air sensitive. The majority of the reported catalysts are metal-based, and in pharmaceutical manufacturing, traces of metals pose a major challenge for their removal to acceptable limits. Therefore, the discovery of efficient, metal-free and mild catalysts for the Ferrier rearrangement is still challenging and desirable especially if such catalysts work well with a wide range of *C-*, *O-*, *N*- and *S*-nucleophiles under mild conditions. We also noted that the use of organocatalysts to catalyze the Ferrier rearrangement is scarcely reported.

Recently, organoboron-catalysis emerged as a mild and effective strategy for activation of alcohols [24], epoxide opening [25–26], Friedel–Crafts alkylations [27], dehydrative glycosylation [28] and many other reactions [29–31]. The robustness and mildness of organoboronic acid catalysts in comparison to traditional strong Lewis and Brønsted acid catalysts inspired us to investigate them as promoters for the Ferrier rearrangement. We envisioned that organoboronic acids can activate the allylic acetate of glycals making them susceptible to nucleophilic attacks under conditions favoring a strong polarization of the allylic acetate moiety (see Figure 4).

Herein, we report a phenylboronic acid-catalyzed synthesis of 2,3-unsaturated *C-, O-*, *N*- and *S*-glycosides via Ferrier rearrangement under very mild conditions. We also demonstrate the scope of the reaction using a wide range of glycals and *C-, O-*, *N*- and *S*-nucleophiles.

## Results and Discussion

We began our study by investigating the reaction of 3,4,6-tri-*O*-acetyl-D-glucal (**1a**) with benzyl alcohol (**2**) in the presence of 20 mol % of arylboronic acids in different solvents (Table 1, entries 1–6). Phenylboronic acid failed to promote the reaction in several solvents and the starting glucal **1a** was recovered unchanged (Table 1, entry 1). This is attributed to its low acidity. Gratifyingly, the more acidic perflurophenylboronic acid successfully promoted the reaction to give 4,6-di-*O*-acetyl-2,3-unsaturated glucoside **3a** in CH_3_CN or CH_3_NO_2_ solvents (Table 1, entries 3 and 4). It gave better 92% yield of glucoside **3a** in CH_3_NO_2_ over a shorter reaction time (Table 1, entries 4 vs 3). Under the same conditions, the reaction did not proceed in THF or DCM due to the lower polarity of these solvents in comparison to CH_3_CN and CH_3_NO_2_ (Table 1, entry 2 vs 3 and 4). Attempts to reduce the amount of perfluorophenylboronic acid to 5 mol % resulted in a reduction of the yield of glucoside **3a** despite various attempts to promote the reaction by increasing the temperature and time (Table 1, entries 5 and 6). In all the cases, the α:β ratio of glucoside **3a** was 90:10. Conditions in Table 1, entry 4 were considered as optimum. The structure of glucoside **3a** was confirmed by the ^1^H NMR spectra where the anomeric proton (H1) appeared at δ 5.16 ppm (for glucal **1a** it appears at δ 6.47 ppm) and the protons of the new double bond (H2, H3) appeared at δ 5.90–5.88 ppm [19]. The corresponding protons in the β-isomer appeared at δ 5.22 (H1) and δ 6.01 (H2, H3) [19,21].

**Table 1:** Optimization of the arylboronic acid-catalyzed reaction of 3,4,6-tri-*O*-acetyl-D-glucal (**1a**) with benzyl alcohol (**2**).

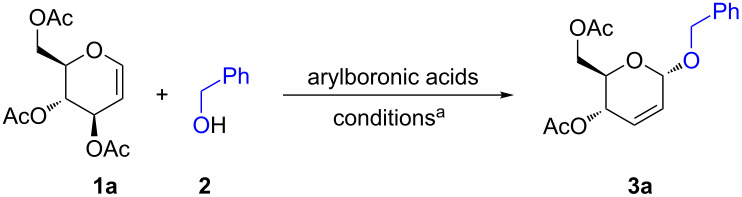				

Entry	Arylboronic acid (mol %)	Solvent	Time (h) / *T* (°C)	**3a** Yield (%) (α:β)^b^

1	phenylboronic acid (20)	CH_3_CN or DCM or THF or CH_3_NO_2_	10/40	ND^c^
2	perfluorophenylboronic acid (20)	DCM or THF	10/40	ND
3	perfluorophenylboronic acid (20)	CH_3_CN	10/40	70 (90:10)
4	perfluorophenylboronic acid (20)	CH_3_NO_2_	6/40	92 (90:10)
5	perfluorophenylboronic acid (10)	CH_3_NO_2_	6/60	88 (90:10)
6	perfluorophenylboronic acid (5)	CH_3_NO_2_	12/60	60 (90:10)

^a^3,4,6-Tri-*O*-acetyl-D-glucal (**1a**, 1 equiv) reacted with benzyl alcohol (**2**, 1.1 equiv). ^b^Isolated yields. α:β ratio calculated from NMR after column chromatography purification. ^c^ND: not detected.

**Figure 1:**
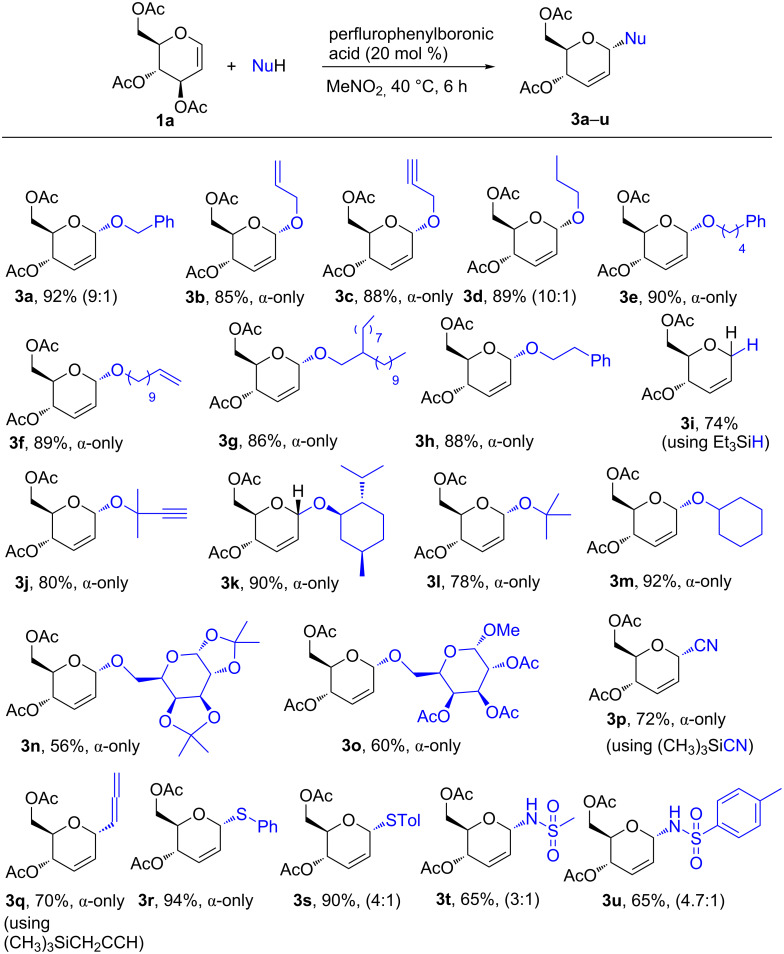
Perfluorophenylboronic acid-catalyzed reaction between 3,4,6-tri-*O*-acetyl-D-glucal **1a** and *O-*, *C-, S*-, *N*-nucleophiles.

Using the optimized conditions in Table 1, entry 4, we then examined the substrate scope. Therefore, glucal **1a** was reacted with various *O*-nucleophiles (using primary, secondary, tertiary, allyl, propargyl alcohols and sugars), *C*-nucleophiles (using trimethylsilyl cyanide and trimethyl(propargyl)silane), *S*-nucleophiles (using thiophenol and *p*-toluenethiol) and *N*-nucleophiles (methane sulfonamide and *p*-toluene sulfonamide) (Figure 1). In all the cases, the reactions successfully gave the respective 2,3-unsaturated glycosides **3a**–**u** in up to 92% yield with mainly α-anomeric selectivity (Table 1). Noteworthy, the reaction also gave disaccharide **3n** and **3o** smoothly with complete α-anomeric selectivity albeit in a moderate yield. Likewise, reaction using Et_3_SiH gave the desired 2,3-unsaturated sugar **3i** in 74% yield. These results testify to the robustness of the perflurophenylboronic acid as a versatile organocatalyst for the Ferrier rearrangement reaction. We noted that the yields of the disaccharides **3n** and **3o** and sulfonamides **3t** and **3u** can be increased with increase in the temperature (60 °C) and extension of the reaction time. The results in Table 1 are superior to the results obtained using boron trifluoride diethyl etherate [32].

**Figure 2:**
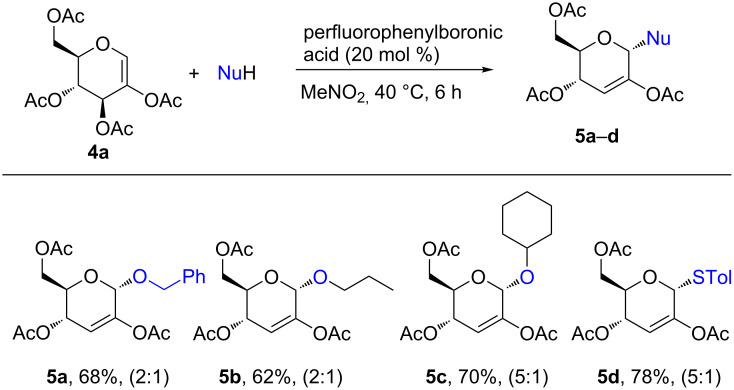
Perfluorophenylboronic acid-catalyzed reaction between 2,3,4,6-tetra-*O*-acetyl-D-glucal **4a** and *O-* and *S*-nucleophiles.

We then applied the perfluorophenylboronic acid catalyst to promote the reaction between 2,3,4,6-tetra-*O*-acetyl-D-glucal (**4a**) and *O*- and *S*-nucleophiles (Figure 2). The Ferrier-catalyzed rearrangements of 2-substituted sugars such as 2,3,4,6-tetra-*O*-acetyl-D-glucal (**4a**) to enosides are limited in the literature and pose special challenges including low product yields and selectivities, the need for a large excess of the catalyst and formation of by-products such as furaldehydes and enones [1,33–35]. Enosides are important building blocks especially for natural product synthesis [36–40]. Therefore, we used the perfluorophenyl boronic acid catalyst in the reaction between 2,3,4,6-tetra-*O*-acetyl-D-glucal (**1a**) and benzyl alcohol, *n*-butyl alcohol, cyclohexyl alcohol and *p*-toluenethiol (Figure 2). Gratifyingly, the reaction proceeded smoothly under mild and catalytic conditions to give the respective 2-acetoxy-2,3-unsaturated glycosides (enosides) **5a**–**d** in 62–78% yields albeit with moderate α-selectivity. No byproducts were detected and no further attempts were made to optimize the yield and selectivity of this reaction. The yields and selectivities are similar to those reported using HClO_4_·SiO_2_ [33].

**Figure 3:**
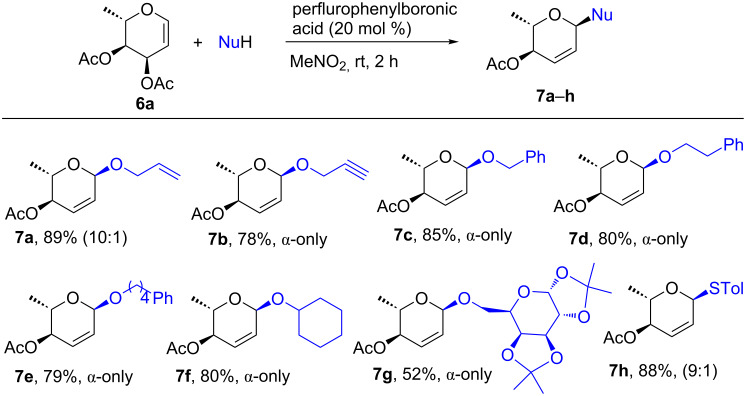
Perfluorophenylboronic-acid-catalyzed reaction between 3,4-di-*O*-acetyl-L-rhamnal (**6a**) and *O-* and *S*-nucleophiles.

Based on the excellent results obtained with the reactions of glucals **1a** and **4a** with *O-*, *C-, N*-, *S*-nucleophiles, we further extended the scope of this reaction to 3,4-di-*O*-acetyl-L-rhamnal (**6a**, Figure 3). As a demonstration, the reaction between 3,4-di-*O*-acetyl-L-rhamnal (**6a**) and selected alcohols and *p*-toluenethiol proceed smoothly and afforded the desired 2,3-unsaturated L-rhamnosides (enosides) **7a**–**h** in up to 89% yield with complete α-anomeric selectivity (except for **7a**). Disaccharide **7g** was also obtained smoothly with complete α-anomeric selectivity. The reactions using rhamnal **6a** was completed at a much faster rate within 2 h at room temperature in comparison to glucals **1a** and **4a** which required ≈6 h at 40 °C to give the products.

**Figure 4:**
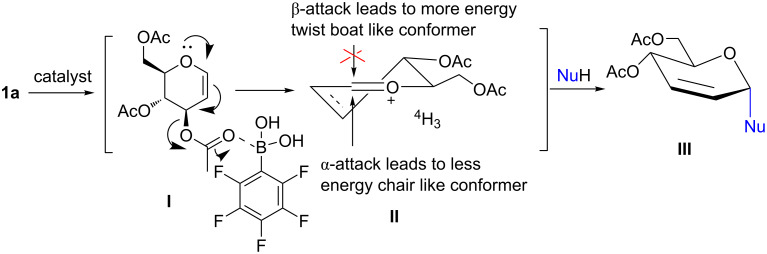
Plausible perfluorophenylboronic acid-catalyzed activation of glycal **1a**.

A plausible pathway of the reaction is proposed in Figure 4. Coordination of perflurophenylboronic acid to the allylic acetate moiety of glucal **1a** induces polarization (structure **I**) and leads to the formation of an allyloxycarbenium ion (structure **II**) in the preferred ^4^H_3_ conformation. Addition of the nucleophiles to C1 from the α-face gives the lower energy half-chair conformer and results in the observed α-selectivity of the 2,3-unsaturated glycosides **III** (Figure 4) [22]. However, the addition of the nucleophiles from the β-face gives the higher energy twist-boat conformer.

## Conclusion

We developed a robust perfluorophenylboronic-acid-catalyzed protocol for the synthesis of a broad range of 2,3-unsaturated *O-*, *C-, S*- and *N*-linked glycosides (enosides) in high yields and mostly α-anomeric selectivity through the reactions of D-glucal **1a**, 2-acetoxy D-glucal **4a** and L-rhamnal **6a** with various *C-*, *O-, N*- and *S*-nucleophiles. Application of this protocol using other glycals is underway in our laboratory.

## Experimental

### General procedure for the synthesis of compounds **3a–u**, **5a–d** and **7a–h**

To a stirred solution of 3,4,6-tri-*O*-acetyl-D-glucal (**1a**, 136 mg, 0.5 mmol) or 2,3,4,6-tetra-*O*-acetyl-D-glucal (**4a**, 165 mg, 0.5 mmol) or 3,4-di-*O*-acetyl-L-rhamnal (**6a**, 107 mg, 0.5 mmol) in anhydrous nitromethane (3 mL) was added the acceptor (0.55 mmol) and perfluorophenylboronic acid (0.1 mmol) at room temperature. In the case of **1a** and **4a**, the resulting solution was stirred at 40 °C for 6 h while in the case of **6a**, it was stirred at room temperature for 2 h (monitor by TLC). The reaction mixture was evaporated under reduced pressure, and the residue was purified using silica gel column chromatography (EtOAc/hexane).

## Supporting Information

File 1 Experimental data and copies of ^1^H and ^13^C NMR spectra of glycosides **3a**–**u**, **5a**–**d** and **7a**–**h** are provided.
