# Supplementary material for: Robust perfluorophenylboronic acid-catalyzed stereoselective synthesis of 2,3-unsaturated O-, C-, N- and S-linked glycosides
Source: Beilstein J Org Chem. 2019 Jun 11;15:1275–80. doi: 10.3762/bjoc.15.125 (PMC6604698; doi:10.3762/bjoc.15.125)

BEILSTEIN JOURNAL OF ORGANIC CHEMISTRY

Supporting Information

for

Robust perfluorophenylboronic acid-catalyzed stereoselective synthesis of 2,3-unsaturated *O*-, *C*-, *N*- and *S*-linked glycosides

Madhu Babu Tatina, Xia Mengxin, Rao Peilin and Zaher M. A. Judeh

Beilstein J. Org. Chem. **2019,** *15,* 1275–1280. doi:10.3762/bjoc.15.125

Experimental data and copies of ^{1}H and ^{13}C NMR spectra of glycosides 3a–u, 5a–d and 7a–h are provided

License and Terms: This is a supporting information file under the terms of the Creative Commons Attribution License (http://creativecommons.org/licenses/by/4.0). Please note that the reuse, redistribution and reproduction in particular requires that the authors and source are credited.
The license is subject to the *Beilstein Journal of Organic Chemistry* terms and conditions: (https://www.beilstein-journals.org/bjoc)

Table of contents

1. **Materials and methods.**
2. **General procedure for the synthesis of compounds 3a–u, 5a–d and 7a–h.**
3. **^{1}H NMR and ^{13}C NMR spectra of glycosides 3a–u, 5a–d and 7a–h.**

1. Materials and methods

Chemical reagents were purchased from Sigma-Aldrich or Alfa Aesar and were used as received without further purification. ^{1}H NMR spectra were recorded at 300 MHz on a Bruker Avance DPX 300. ^{13}C NMR spectra were recorded at 75.47 MHz on a Bruker Avance DPX 300. Unless stated otherwise, data refer to solutions in $CDCl_3$ with TMS as an internal reference. HRMS were recorded on a Qstar XL MS/MS system. Analytical TLC was performed using Merck 60 F_{254} precoated silica gel plates (0.2 mm thickness) and visualized using UV radiation (254 nm) or stained using ceric ammonium nitrate in 30% H_2SO_4 solution. Flash chromatography was performed using Merck silica gel 60 (60–120 mesh).

2. General procedure for the synthesis of compounds 3a-3u, 5a-5d and 7a-7h

To a stirred solution of 3,4,6-tri-*O*-acetyl-D-glucal (**1a**, 136 mg, 0.5 mmol) or 2,3,4,6-tetra-*O*-acetyl-D-glucal (**4a**, 165 mg, 0.5 mmol) or 3,4-di-*O*-acetyl-L-rhamnal **6a** (107 mg, 0.5 mmol) in anhydrous nitromethane (3 mL) was added the acceptor (0.55 mmol) and perfluorophenylboronic acid (0.1 mmol) at room temperature. In the case of **1a** and **4a**, the resulting solution was stirred at 40 °C for 6 h while in the case of **6a**, it was stirred at room temperature for 2 h (monitored by TLC). The reaction mixture was evaporated under reduced pressure, and the residue was purified using silica gel column chromatography (EtOAc/hexane).

2.1. Benzyl 4,6-di-*O*-acetyl-2,3-dideoxy-α-D-erythro-hex-2-enopyranoside (3a)[1]

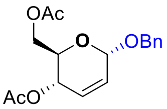

Prepared by the general procedure using **1a** (0.5 mmol, 136 mg) and benzyl alcohol (0.55 mmol, 58 µL). Column chromatography purification using EtOAc/hexane (2:8) gave **3a** as white solid (147 mg, 92%). ^{1}H NMR (300 MHz, $CDCl_3$) δ 7.37 (m, 5H), 5.89 (m, 2H), 5.36 (d, *J* = 9.5, 1H), 5.16 (s, 1H), 4.83 (d, *J* = 11.7 Hz, 1H), 4.62 (d, *J* = 11.7 Hz, 1H), 4.31 – 4.22 (m, 1H), 4.21 – 4.08 (m, 1H), 2.12 (s, 3H), 2.10 (s, 3H). ^{13}C NMR (75 MHz, $CDCl_3$) δ 170.8, 170.3, 137.6, 129.3, 128.5, 128.0, 127.9, 127.7, 126.9, 93.6, 70.3, 67.1, 65.3, 62.9, 20.9, 20.8.

2.2 Allyl 4,6-di-*O*-acetyl-2,3-dideoxy-α-D-erythro-hex-2-enopyranoside (3b)[1]

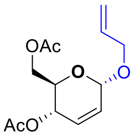

Prepared by the general procedure using **1a** (0.5 mmol, 136 mg) and allyl alcohol (0.55 mmol, 38 µL). Column chromatography purification using EtOAc/hexane (2:8) gave **3b** as white solid (114 mg, 85%). ^{1}H NMR (300 MHz, $CDCl_3$) δ 5.98 – 5.72 (m, 3H), 5.29 – 5.19 (m, 2H), 5.14 (dd, *J* = 10.3, 1.5 Hz, 1H), 5.01 (s, 1H), 4.26 – 4.11 (m, 3H), 4.12 – 3.96 (m,

2H), 2.04 (s, 3H), 2.02 (s, 3H). ^{13}C NMR (75 MHz, $CDCl_3$) δ 169.8, 169.3, 133.1, 128.2, 126.7, 116.5, 92.6, 68.3, 65.9, 64.3, 61.9, 19.9, 19.8.

2.3 Propargyl 4,6-di-*O*-acetyl-2,3-dideoxy-α-D-erythro-hex-2-enopyranoside (3c)[1]

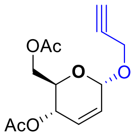

Prepared by the general procedure **1a** (0.5 mmol, 136 mg) and propargyl alcohol (0.55 mmol, 32 µL). Column chromatography purification using EtOAc/hexane (2:8) gave **3c** as white solid (117 mg, 88%). ^{1}H NMR (300 MHz, $CDCl_3$) δ 5.98 – 5.81 (m, 2H), 5.35 (dd, *J* = 9.5, 1.3 Hz, 1H), 5.25 (s, 1H), 4.40 – 4.15 (m, 2H), 4.11 (m, 1H), 2.48 (t, *J* = 2.4 Hz, 1H), 2.12 (s, 2H), 2.10 (s, 2H). ^{13}C NMR (75 MHz, $CDCl_3$) δ 170.7, 170.2, 129.8, 127.2, 92.7, 79.1, 74.8, 67.2, 65.1, 62.7, 55.0, 20.9, 20.8.

2.4 *n*-propyl 4,6-di-*O*-acetyl-2,3-dideoxy-α-D-erythro-hex-2-enopyranoside (3d)[1]

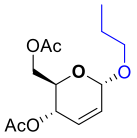

Prepared by the general procedure using **1a** (0.5 mmol, 136 mg) and *n*-propanol (0.55 mmol, 41 µl). Column chromatography purification using EtOAc/hexane (2:8) gave **3d** as colourless liquid (121 mg, 89%). ^{1}H NMR (300 MHz, $CDCl_3$) δ 5.88 – 5.76 (m, 2H), 5.28 (dd, *J* = 9.6, 1.3 Hz, 1H), 4.98 (s, 1H), 4.25 – 4.15 (m, 2H), 4.15 – 4.06 (m, 1H), 3.69 (dd, *J* = 11.5, 4.7 Hz, 1H), 3.46 (dt, *J* = 9.5, 6.5 Hz, 1H), 1.59 (m, 2H), 0.91 (t, *J* = 7.4 Hz, 3H).

2.5 Benzenebutyl 4,6-di-*O*-acetyl-2,3-dideoxy-α-D-erythro-hex-2-enopyranoside (3e)[2]

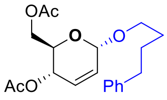

Prepared by the general procedure using **1a** (0.5 mmol, 136 mg) and 4-Phenyl-1-butanol (0.55 mmol, 84 µL). Column chromatography purification using EtOAc/hexane (2:8) gave **3e** as colourless liquid (115 mg, 90%). ^{1}H NMR (300 MHz, $CDCl_3$) δ 7.29 (dd, *J* = 5.7, 1.4 Hz, 3H), 7.23 – 7.15 (m, 2H), 5.96 – 5.77 (m, 2H), 5.33 (dd, *J* = 9.6, 1.4 Hz, 1H), 5.03 (s, 1H), 4.30 – 4.22 (m, 1H), 4.20 (d, *J* = 2.4 Hz, 1H), 4.17 – 4.07 (m, 1H), 3.87 – 3.77 (m, 1H), 3.54 (dt, *J* = 9.6, 6.1 Hz, 1H) 2.67 (t, *J* = 7.3 Hz, 2H), 2.10 (s, 3H), 2.09 (s, 3H), 1.76 – 1.64 (m, 4H). ^{13}C NMR (75 MHz, $CDCl_3$) δ 170.8, 170.3, 142.2, 129.0, 128.4, 128.33, 128.30, 127.9, 125.7, 94.4, 68.7, 66.9, 65.3, 63.0, 35.6, 29.3, 28.1, 20.9, 20.7.

2.6 10-Undecen-1-yl 4,6-di-*O*-acetyl-2,3-dideoxy-α-D-erythro-hex-2-enopyranoside (3f)

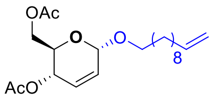

Prepared by the general procedure using **1a** (0.5 mmol, 136 mg) and 10-undecen-1-ol (0.55 mmol, 110 µL). Column chromatography purification using EtOAc/hexane (1:9) gave **3f** as colourless liquid (169 mg, 89%). $[\alpha]_D^{20}$ +63.617 (c, 1.4, $CHCl_3$). ^1H NMR (300 MHz, $CDCl_3$) δ 5.92 – 5.75 (m, 3H), 5.33 (dd, *J* = 9.6, 1.3 Hz, 1H), 5.03 (s, 1H), 5.00 – 4.90 (m, 2H), 4.32 – 4.19 (m, 2H), 4.19 – 4.00 (m, 1H), 3.78 (dt, *J* = 9.5, 6.7 Hz, 1H), 3.51 (dt, *J* = 9.5, 6.6 Hz, 1H), 2.11 (s, 3H), 2.10 (s, 3H),2.08 – 2.00 (m, 2H) 1.61 (m, 3H), 1.30 (s, 9H), 1.00 – 0.74 (m, 2H). ^{13}C NMR (75 MHz, $CDCl_3$) δ 170.8, 170.3, 139.1, 128.9, 127.9, 114.1, 94.4, 69.0, 66.8, 65.3, 63.0, 33.7, 29.7, 29.5, 29.4, 29.3, 29.2, 28.9, 26.2, 20.9, 20.8. HRMS (ESI+): m/z [M + Na]$^+$ calcd for $C_{21}H_{34}O_6Na$: 405.2253; found: 405.2268.

2.7 2-Octyl-dodecan-1-yl 4,6-di-*O*-acetyl-2,3-dideoxy-α-D-erythro-hex-2-enopyranoside (3g)

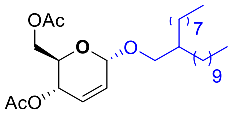

Prepared by the general procedure using **1a** (0.5 mmol, 136 mg) and 2-Octyl-1-dodecanol (0.55 mmol, 195 µL). Column chromatography purification using EtOAc/hexane (0.5:9.5) gave **3g** as colourless liquid (219 mg, 86%). $[\alpha]_D^{20}$ +25.984 (c, 1.9, $CHCl_3$). ^1H NMR (300 MHz, $CDCl_3$) δ 5.97 – 5.80 (m, 2H), 5.33 (dd, *J* = 9.7, 1.2 Hz, 1H), 5.01 (s, 1H), 4.28 (dd, = 11.9, 5.4 Hz, 1H), 4.18 (dd, *J* = 12.1, 2.3 Hz, 1H), 4.14 – 3.98 (m, 1H), 3.70 (dd, *J* = 9.4, 6.2 Hz, 1H), 3.37 (dd, *J* = 9.3, 5.6 Hz, 1H), 2.12 (s, 3H), 2.10 (s, 3H), 1.65 (s, 1H), 1.28 (s, 32H), 0.90 (t, *J* = 6.7 Hz, 6H). ^{13}C NMR (75 MHz, $CDCl_3$) δ 170.8, 170.3, 128.8, 128.0, 94.6, 72.0, 66.9, 65.6, 65.3, 63.0, 40.5, 38.1, 31.91, 31.9, 30.9,30.0, 29.64, 29.61, 29.3, 26.8,26.7, 22.6, 20.9, 20.7, 14.0. HRMS (ESI+): m/z [M + Na]$^+$ calcd for $C_{30}H_{54}O_6Na$: 533.3818; found: 533.3812

2.8 Benzeneethyl 4,6-di-*O*-acetyl-2,3-dideoxy-α-D-erythro-hex-2-enopyranoside (3h)[2]

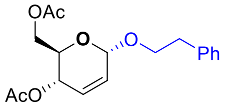

Prepared by the general procedure using **1a** (0.5 mmol, 136 mg) and 1-phenylethanol (0.55 mmol, 66 µL). Column chromatography purification using EtOAc/hexane (2:8) gave **3h** as colourless liquid (146 mg, 88%). ^1H NMR (300 MHz, $CDCl_3$) δ 7.36 – 7.27 (m, 3H), 7.24 (d, *J* = 4.5 Hz, 2H), 5.96 – 5.78 (m, 2H), 5.31 (dd, *J* = 9.7, 1.4 Hz, 1H), 5.04 (s, 1H), 4.21 (dd, *J* = 12.1, 5.2 Hz, 1H), 4.09 (d, *J* = 2.3 Hz, 1H), 4.06 – 3.95 (m, 2H), 3.78 (dt, *J* = 9.7, 6.9 Hz, 1H), 2.95 (t, *J* = 7.0 Hz, 2H), 2.10 (s, 3H), 2.09 (s, 3H). ^{13}C NMR (75 MHz, $CDCl_3$)

δ 170.8, 170.3, 138.7, 129.1, 128.9, 128.3, 127.7, 126.3, 94.4, 69.5, 66.9, 65.2, 62.9, 36.35, 20.9, 20.7.

2.9 ((2R,3S)-3-Acetoxy-3,6-dihydro-2*H*-pyran-2-yl) methyl acetate (3i)[3]

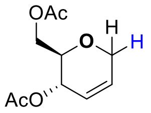

Prepared by the general procedure using **1a** (0.5 mmol, 136 mg) and triethylsilane (0.55 mmol, 88 µL). Column chromatography purification using EtOAc/hexane (2:8) gave 3i as colourless liquid (79 mg, 74%). ^{1}H NMR (400 MHz, $CDCl_3$) δ 5.95−5.92 (m, 1H), 5.78-5.75 (ddd, *J* = 9.4 Hz, 3.6 Hz, 1.8 Hz, 1H), 5.27−5.24 (1H, m), 4.24−4.15 (m, 4H), 3.75-3.70 (m, 1H), 2.10 (3H, s), 2.08 (3H, s). ^{13}C NMR (100 MHz, $CDCl_3$) δ 170.9, 170.3, 129.4, 124.2, 73.8, 65.2, 65.0, 63.2, 21.0, 20.8

2.10 2-Methyl-3-butyn-2-yl 4,6-di-*O*-acetyl-2,3-dideoxy-α-D-erythro-hex-2-enopyranoside (3j)

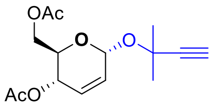

Prepared by the general procedure using **1a** (0.5 mmol, 136 mg) and 2-methyl-3-butyn-2-ol (0.55 mmol, 53 µL). Column chromatography purification using EtOAc/hexane (2:8) gave **3j** as colourless liquid (118 mg, 80%). $[\alpha]_D^{20}$ +113.627 (c, 0.07, $CHCl_3$). ^{1}H NMR (300 MHz, $CDCl_3$) δ 5.90 (d, *J* = 10.3 Hz, 1H), 5.86 – 5.74 (m, 1H), 5.67 (s, 1H), 5.29 (m, 1H), 4.27 – 4.22 (m, 1H), 4.17 (m, 2H), 2.54 (s, 1H), 2.10 (s, 6H), 1.63 (s, 3H), 1.61 (s, 3H). ^{13}C NMR (75 MHz, $CDCl_3$) δ 170.8, 170.3, 128.9, 128.5, 90.9, 85.3, 73.3, 71.6, 67.0, 65.2, 63.1, 30.54, 29.88, 21.0, 20.8. HRMS (ESI$^+$): m/z $[M + Na]^+$ calcd for $C_{15}H_{20}O_6Na$: 319.1158; found: 319.1138.

2.11 L-Menthyl 4,6-di-*O*-acetyl-2,3-dideoxy-α-D-erythrohex-2-enopyranoside (3k)[4]

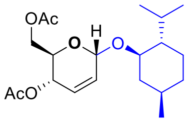

Prepared by the general procedure using **1a** (0.5 mmol, 136 mg) and L-menthol (0.55 mmol, 86 mg). Column chromatography purification using EtOAc/hexane (2:8) gave **3k** as colourless liquid (165 mg, 90%). ^{1}H NMR (300 MHz, $CDCl_3$) δ 5.87 (s, 2H), 5.29 (d, *J* = 8.7 Hz, 1H), 5.11 (s, 1H), 4.30 – 4.13 (m, 3H), 3.53 – 3.33 (m, 1H), 2.19 (m, 1H), 2.12 (s, 3H), 2.08 (s, 3H), 1.66 (m, 2H), 1.51 – 1.36 (m, 1H), 1.33 – 1.19 (m, 2H), 1.15 – 0.99 (m, 2H), 0.98 – 0.85 (m, 6H), 0.82 (d, *J* = 6.9 Hz, 1H), 0.78 (d, *J* = 7.0 Hz, 3H). ^{13}C NMR (75 MHz, $CDCl_3$) δ 170.8, 170.3, 128.5, 128.0, 96.1, 81.0, 66.7, 65.3, 63.3, 48.8, 43.3, 34.2, 31.8, 25.6, 23.1, 22.4, 21.1, 20.9, 20.8, 16.2.

2.12 *tert*-butyl 4,6-di-*O*-acetyl-2,3-dideoxy-α-D-erythro-hex-2-enopyranoside (3l)[5]

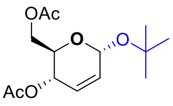

Prepared by the general procedure using **1a** (0.5 mmol, 136 mg) and *tert*-butanol (0.55 mmol, 53 µL). Column chromatography purification using EtOAc/hexane (2:8) gave 3l as colourless liquid (111 mg, 78%). ^{1}H NMR (300 MHz, $CDCl_3$) δ 5.86 (d, *J* = 10.2 Hz, 1H), 5.81 – 5.72 (m, 1H), 5.36 – 5.33 (m, 1H), 5.32 – 5.25 (m, 1H), 4.32 – 4.12 (m, 3H), 2.10 (s, 3H), 2.09 (s, 3H), 1.31 (s, 9H). ^{13}C NMR (75 MHz, $CDCl_3$) δ 170.8, 170.4, 129.5, 128.1, 88.8, 75.1, 66.6, 65.3, 63.2, 28.4, 21.0, 20.8.

2.13 Cyclohexyl 4,6-di-*O*-acetyl-2,3-dideoxy-α-D-erythro-hex-2-enopyranoside (3m)[1]

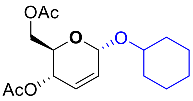

Prepared by the general procedure using **1a** (0.5 mmol, 136 mg) and cyclohexanol (0.55 mmol, 57 µL). Column chromatography purification using EtOAc/hexane (2:8) gave **3m** as colourless liquid (143 mg, 92%). ^{1}H NMR (300 MHz, $CDCl_3$) δ 6.03 – 5.72 (m, 2H), 5.31 (dd, *J* = 9.2, 1.3 Hz, 1H), 5.18 (s, 1H), 4.34 – 4.09 (m, 3H), 3.77 – 3.55 (m, 1H), 2.1 (s, 3H), 2.09 (s, 3H), 1.92 (m, 2H) 1.71 (m, 3H), 1.57 (m, 1H), 1.30 (m, 5H). ^{13}C NMR (75 MHz, $CDCl_3$) δ 170.8, 170.3, 128.7, 128.5, 92.7, 76.7, 66.7, 65.4, 63.1, 33.7, 32.1, 25.5, 24.4, 24.1, 20.9, 20.7.

2.14 4,6-Di-*O*-acetyl-2,3-dideoxy-D-threo-hex-2-enopyranoside-(α1-6)-1,2:3,4-di-*O*-isopropylidene-D-galactopyranoside (3n)[6a]

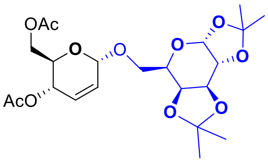

Prepared by the general procedure using **1a** (0.5 mmol, 136 mg) and 1,2:3,4-di-*O*-isopropylidene-α-D-galactopyranose (0.55 mmol, 143 mg). Column chromatography purification using EtOAc/hexane (2:8) gave **3n** as white solid (132 mg, 56%). ^{1}H NMR (300 MHz, $CDCl_3$) δ 5.93 – 5.79 (m, 2H), 5.54 (d, *J* = 5.0 Hz, 1H), 5.54 (d, *J* = 5.0 Hz, 1H), 5.11 (s, 1H), 4.64 (dd, *J* = 7.9, 2.4 Hz, 1H), 4.37 – 4.31 (m, 1H), 4.31 – 4.23 (m, 2H), 4.21 – 4.09 (m, 2H), 4.09 – 3.96 (m, 1H), 3.89 (dd, *J* = 10.2, 6.3 Hz, 1H), 3.77 (dd, *J* = 10.1, 7.0 Hz, 1H), 2.12 (s, 3H), 2.10 (s, 3H), 1.55 (s, 3H), 1.46 (s, 3H), 1.36 (s, 3H), 1.35 (s, 3H). ^{13}C NMR (75 MHz, $CDCl_3$) δ 170.9, 170.3, 129.2, 127.7, 109.3, 108.6, 96.3, 94.6, 70.9, 70.6, 70.5, 67.0, 66.9, 66.2, 65.2, 62.9, 26.05, 26.00, 24.9, 24.5, 20.9, 20.8.

2.15 Methyl-α-D-[2,3,4-Tri-*O*-acetyl-6-*O*-(4',6'-di-*O*-acetyl-2',3'-dideoxy-a-D-hex-2' enopyranosyl)]glucopyranoside (3o)[6b]

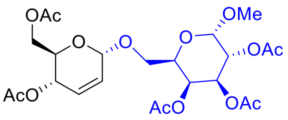

Prepared by the general procedure using **1a** (0.5 mmol, 136 mg) and 2,3,4-tri-O-acetyl-α-D-methylglucoopyranoside (0.55 mmol, 176 mg). Column chromatography purification using EtOAc:hexane (3:7) solvent system gave 3o as white solid (159 mg, 60% yield). ^{1}H NMR (300 MHz, CDCl3): δ = 5.89 (m, 2H), 5.50 (t, *J* = 9.3 Hz, 1H), 5.36 (dd, *J* = 9.7, 1.4 Hz, 1H), 5.23 – 5.13 (m, 1H), 5.07 (s, 1H), 4.97 (d, *J* = 3.6 Hz, 1H), 4.91 (dd, *J* = 10.2, 3.6 Hz, 1H), 4.30 (dd, *J* = 12.4, 4.5 Hz, 1H), 4.17 – 4.04 (m, 2H), 3.95 (dt, *J* = 10.0, 3.4 Hz, 1H), 3.87 (dd, *J* = 11.1, 3.9 Hz, 1H), 3.65 (dd, *J* = 11.1, 2.7 Hz, 1H), 3.43 (s, 3H), 2.12 (s, 3H), 2.11 (s, 3H), 2.10 (s, 3H), 2.06 (s, 3H), 2.03 (s, 3H).

2.16 4,6-Di-*O*-acetyl-2,3-dideoxy-α-D-erythro-hex-2-enopyranosyl cyanide (3p)[8]

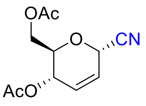

Prepared by the general procedure using **1a** (0.5 mmol, 136 mg) and Trimethylsilyl cyanide (1.0 mmol, 125 µL). Column chromatography purification using EtOAc/hexane (2:8) gave **3p** as colourless liquid (86 mg, 72%). ^{1}H NMR (300 MHz, $CDCl_3$) δ 6.05 (dt, *J* = 10.2, 1.9 Hz, 1H), 5.90 (ddd, *J* = 10.2, 3.5, 1.9 Hz, 1H), 5.35 (dd, *J* = 9.1, 2.0 Hz, 1H), 5.15 – 5.06 (m, 1H), 4.27 (d, *J* = 3.9 Hz, 2H), 4.08 – 4.01 (m, 1H), 2.13 (s, 3H), 2.12 (s, 3H). ^{13}C NMR (75 MHz, $CDCl_3$) δ 170.6, 170.0, 129.7, 123.5, 115.5, 72.0, 62.7, 62.6, 62.2, 20.8, 20.7

2.17 4,6-di-*O*-acetyl-2,3-dideoxy-α-D-*erythro*-hex-2-enopyranoside propadiene (3q)[9]

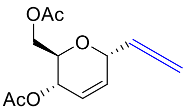

Prepared by the general procedure using **1a** (0.5 mmol, 136 mg) and Trimethyl(propargyl)silane (0.55 mmol, 82 µL). Column chromatography purification using EtOAc/hexane (2:8) gave **3q** as colourless liquid (88 mg, 70%). ^{1}H NMR (300 MHz, $CDCl_3$) δ 5.93 (ddd, *J* = 10.3, 2.8, 1.7 Hz, 1H), 5.87 – 5.74 (m, 1H), 5.36 – 5.22 (m, 2H), 4.92 – 4.89 (m, 2H), 4.34 (s, 1H), 4.22 (dd, *J* = 4.2, 2.3 Hz, 2H), 3.93 (ddd, *J* = 8.4, 5.0, 3.3 Hz, 1H), 2.12 (s, 3H), 2.10 (s, 3H). ^{13}C NMR (75 MHz, $CDCl_3$) δ 209.1, 171.0, 170.4, 130.6, 125.0, 89.3, 77.2, 70.5, 68.8, 65.0, 63.1, 21.0, 20.8.

2.18 Phenyl 4,6-di-*O*-acetyl-2,3-dideoxy-1-thio-α-D-*erythro*-hex-2-enopyranoside (3r)[7]

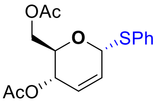

Prepared by the general procedure using **1a** (0.5 mmol, 136 mg) and thiophenol (0.55 mmol, 56 µL). Column chromatography purification using EtOAc/hexane (2:8) gave **3r** as colourless liquid (151 mg, 94%). ^{1}H NMR (300 MHz, $CDCl_3$) δ 7.54 – 7.40 (m, 2H), 7.29 – 7.12 (m, 3H), 5.99 (ddd, *J* = 10.1, 3.1, 1.9 Hz, 1H), 5.79 (dt, *J* = 10.1, 1.7 Hz, 1H), 5.69 (bs, 1H), 5.31 (dd, *J* = 9.5, 1.9 Hz, 1H), 4.46 – 4.31 (m, 1H), 4.30 – 4.09 (m, 2H), 2.04 (s, 3H), 2.00 (s, 3H). ^{13}C NMR (75 MHz, $CDCl_3$) δ 170.8, 170.3, 134.7, 131.8, 128.9, 128.5, 127.6, 127.6, 83.7, 67.3, 65.1, 63.1, 21.0, 20.8.

2.19 4-methylPhenyl 4,6-di-*O*-acetyl-2,3-dideoxy-1-thio-α-D-*erythro*-hex-2-enopyranoside (3s)[7]

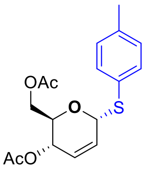

Prepared by the general procedure using **1a** (0.5 mmol, 136 mg) and 4-Methylbenzenethiol (0.55 mmol, 68 mg). Column chromatography purification using EtOAc/hexane (2:8) gave **3s** as light yellow liquid (151 mg, 90%). ^{1}H NMR (300 MHz, $CDCl_3$) δ 7.46 (d, *J* = 8.1 Hz, 2H), 7.14 (d, *J* = 8.0 Hz, 2H), 6.08 (ddd, *J* = 10.1, 3.1, 1.9 Hz, 1H), 5.93 – 5.80 (m, 1H), 5.70 (d, *J* = 1.3 Hz, 1H), 5.39 (dd, *J* = 9.5, 1.9 Hz, 1H), 4.56 – 4.44 (m, 1H), 4.27 (dd, *J* = 6.1, 4.3 Hz, 1H), 2.36 (s, 3H), 2.13 (s, 3H), 2.11 (s, 3H).

2.20 4,6-Di-*O*-acetyl-2,3-dideoxy-α-D-erythro-hex-2-enopyranosyl-4-methanesulfonamide (3t)[10]

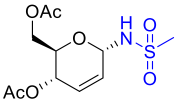

Prepared by the general procedure using **1a** (0.5 mmol, 136 mg) and Methanesulfonamide (0.55 mmol, 52 mg). Column chromatography purification using EtOAc:Hexane (3:7) solvent system gave **3t** as colourless liquid (99 mg, 65%). ^{1}H NMR (300 MHz, $CDCl_3$) δ 6.01 (d, *J* = 10.2 Hz, 1H), 5.88 (d, *J* = 12.3 Hz, 1H), 5.57 (q, *J* = 9.2 Hz, 2H), 5.26 (d, *J* = 9.0 Hz, 1H), 4.39 – 4.06 (m, 3H), 4.03 – 3.83 (m, 1H), 3.16 (s, 3H), 2.13 (s, 3H), 2.08 (s, 3H). ^{13}C NMR (75 MHz, $CDCl_3$) δ 170.5, 170.1, 130.3, 126.6, 76.7, 67.4, 64.7, 63.2, 43.0, 20.9, 20.7.

2.21 4,6-Di-*O*-acetyl-2,3-dideoxy-α-D-erythro-hex-2-enopyranosyl-4 methylbenzenesulfonamide (3u)[10]

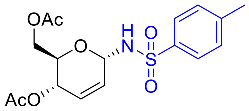

Prepared by the general procedure using **1a** (0.5 mmol, 136 mg) and p-toluenesulfonamide (0.55 mmol, 94 mg). Column chromatography purification using EtOAc:Hexane (3:7) solvent system gave **3u** as *colourless* liquid (124 mg, 65%). ^{1}H NMR (300 MHz, $CDCl_3$) δ 7.82 (d, *J* = 8.3 Hz, 2H), 7.37 – 7.26 (m, 2H), 6.03 (d, *J* = 8.8 Hz, 1H), 5.92 (d, *J* = 10.1 Hz, 1H), 5.81 (ddd, *J* = 10.1, 3.0, 1.9 Hz, 1H), 5.26 (dd, *J* = 9.3, 1.8 Hz, 1H), 3.89 (dd, *J* = 12.2, 3.4 Hz, 1H), 3.56 (dt, *J* = 9.2, 3.1 Hz, 1H), 3.36 (dd, *J* = 12.2, 2.7 Hz, 1H), 2.44 (s, 1H), 2.03 (s, 3H), 2.02 (s, 3H). ^{13}C NMR (75 MHz, $CDCl_3$) δ 170.6, 170.0, 143.8, 138.6, 130.4, 129.5, 127.1, 126.7, 66.7, 64.2, 61.8, 21.5, 20.8, 20.7.

2.22 Benzyl 2,4,6-tri-*O*-acetyl-3-deoxy-α/β-D-erythro-hex-2-enopyranoside (5a)[11]

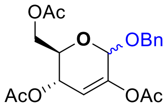

Prepared by the general procedure using **4a** (0.5 mmol, 165 mg) and benzyl alcohol (0.55 mmol, 57 µL). Column chromatography purification using EtOAc/hexane (2:8) gave **5a** as colourless liquid (128 mg, 68%). ^{1}H NMR (300 MHz, $CDCl_3$) δ 7.38 (m, 7H), 5.77 (d, *J* = 2.1 Hz, 1H), 5.50 (dd, *J* = 9.3, 1.5 Hz, 1H), 5.35 – 5.23 (m, 2H), 5.15 (s, 1H), 4.83 (d, *J* = 11.8 Hz, 1H), 4.63 (m, 1H), 4.35 (t, *J* = 5.9 Hz, 1H), 4.30 – 4.05 (m, 3H), 2.14 (s, 3H), 2.12 (s, 3H), 2.09 (s, 3H). ^{13}C NMR (75 MHz, $CDCl_3$) δ 170.8, 170.1, 168.2, 146.3, 140.9, 137.3, 128.5, 127.9, 127.6, 115.4, 112.2, 111.3, 93.06, 92.24, 72.6, 70.5, 69.9, 67.3, 63.2, 62.4, 20.93. 20.9, 20.8.

2.23 *n*-Propyl 2,4,6-tri-*O*-acetyl-3-deoxy-α/β-D-erythro-hex-2-enopyranoside (5b)[11]

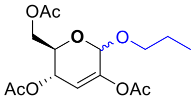

Prepared by the general procedure using **4a** (0.5 mmol, 165 mg) and *n*-propanol (0.55 mmol, 41 µL). Column chromatography purification using EtOAc/hexane (2:8) gave **5b** as colourless liquid (102 mg, 62%). ^{1}H NMR (300 MHz, $CDCl_3$) δ 5.74 (d, *J* = 2.1 Hz, 1H), 5.47 (dd, *J* = 9.4, 1.3 Hz, 1H), 5.08 (s, 1H), 4.29 (dd, *J* = 6.0, 2.9 Hz, 1H), 4.27 – 4.20 (m, 2H), 4.17 – 4.09 (m, 1H), 3.84 – 3.68 (m, 1H), 3.57 – 3.43 (m, 3H), 2.19 (s, 4H), 2.12 (s, 3H), 2.09 (s, 4H), 1.70 – 1.59 (m, 4H), 0.96 (t, *J* = 7.6 Hz, 5H).

2.24 Cyclohexyl 2,4,6-tri-*O*-acetyl-3-deoxy-α/β-D-erythro-hex-2-enopyranoside (5c)[11]

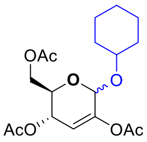

Prepared by the general procedure using **4a** (0.5 mmol, 165 mg) and cyclohexanol (0.55 mmol, 57 µL). Column chromatography purification using EtOAc/hexane (2:8) gave 5c as colourless liquid (129 mg, 70%). ^{1}H NMR (300 MHz, $CDCl_3$) δ 5.73 (s, 1H), 5.43 (m, 1H), 5.21 (s, 1H), 4.36 – 4.04 (m, 3H), 3.64 (dt, *J* = 9.1, 4.3 Hz, 1H), 2.16 (s, 3H), 2.10 (s, 3H), 2.08 (s, 3H), 1.97 – 1.68 (m, 4H), 1.58 – 1.38 (m, 2H), 1.38 – 1.19 (m, 4H). ^{13}C NMR (75 MHz, $CDCl_3$) δ 170.7, 170.1, 168.2, 146.9, 114.95, 92.51, 67.0, 65.4, 62.7, 33.5, 31.9, 25.4, 24.1, 23.8, 20.9, 20.8, 20.7.

2.25 4-Methylphenyl 2,4,6-tri-*O*-acetyl-3-deoxy-α/β-1-thio-D-erythro-hex-2-enopyranoside (5d)[11]

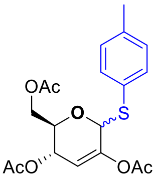

Prepared by the general procedure using **4a** (0.5 mmol, 165 mg) and 4-Methyl benzenethiol (0.55 mmol, 68 mg). Column chromatography purification using EtOAc/hexane (2:8) gave **5d** as light yellow colour liquid (105 mg, 78%). ^{1}H NMR (300 MHz, $CDCl_3$) δ 7.44 (d, *J* = 8.1 Hz, 2H), 7.14 (d, *J* = 8.2 Hz, 2H), 5.78 – 5.62 (m, 2H), 5.53 – 5.46 (m, 1H), 4.60 – 4.48 (m, 1H), 4.27 (dd, *J* = 8.6, 4.8 Hz, 2H), 2.35 (s, 3H), 2.22 (s, 3H), 2.12 (s, 3H), 2.11 (s, 3H). ^{13}C NMR (75 MHz, $CDCl_3$) δ 170.7, 170.1, 168.0, 146.7, 138.2, 134.1, 132.7, 130.1, 129.8, 129.5, 115.1, 83.9,81.6, 74.6, 67.5, 65.1, 64.9, 62.7, 21.1, 20.9, 20.7.

2.26 Allyl 4-*O*-acetyl-2,3,6-trideoxy-α-D-erythro-hex-2-enopyranoside (7a)[12]

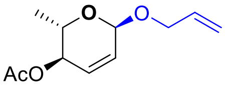

Prepared by the general procedure using **6a** (0.5 mmol, 107 mg) and allyl alcohol (0.55 mmol, 38 µL). Column chromatography purification using EtOAc/hexane (1:9) gave **7a** as colourless liquid (94 mg, 89%). ^{1}H NMR (300 MHz, $CDCl_3$) δ 6.04 – 5.76 (m, 3H), 5.32 (dd, *J* = 17.2, 1.6 Hz, 1H), 5.26 – 5.15 (m, 1H), 5.07 (dd, *J* = 9.2, 1.4 Hz, 1H), 5.03 (s, 1H), 4.27 (ddt, *J* = 6.6, 5.2, 1.4 Hz, 1H), 4.13 – 3.96 (m, 2H), 2.10 (s, 3H), 1.24 (d, *J* = 6.3

Hz,3H). ^{13}C NMR (75 MHz, $CDCl_3$) δ 170.54, 129.4, 127.9, 94.3, 70.9, 70.3, 64.6, 23.0, 21.0, 17.9, 10.6.

2.27 Propargyl 4-*O*-acetyl-2,3,6-trideoxy-α-D-erythro-hex-2-enopyranoside (7b)[12]

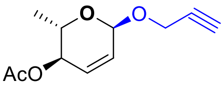

Prepared by the general procedure using **6a** (0.5 mmol, 107 mg) and propargyl alcohol(0.55 mmol, 32 µL). Column chromatography purification using EtOAc/hexane (1:9) gave **7b** as colourless liquid (81 mg, 78%). ^{1}H NMR (300 MHz, $CDCl_3$) δ 6.02 – 5.73 (m, 2H), 5.19 (s, 1H), 5.09 (dd, *J* = 9.2, 1.5 Hz, 1H), 4.32 (d, *J* = 2.4 Hz, 2H), 4.05 – 3.89 (m, 1H), 2.46 (t, *J* = 2.4 Hz, 1H), 2.10 (s, 3H), 1.25 (d, *J* = 6.3 Hz, 3H).

2.28 Benzyl 4-*O*-acetyl-2,3,6-trideoxy-α-D-erythro-hex-2-enopyranoside (7c)[12]

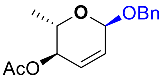

Prepared by the general procedure using **6a** (0.5 mmol, 107 mg) and Benzyl alcohol (0.55 mmol, 57 µL). Column chromatography purification using EtOAc/hexane (1:9) gave **7c** as colourless liquid (111 mg, 85%). 1H NMR (300 MHz, $CDCl_3$) δ 7.44 – 7.23 (m, 5H), 5.98 – 5.74 (m, 2H), 5.16 – 5.00 (m, 2H), 4.81 (d, *J* = 11.9 Hz, 1H), 4.63 (d, *J* = 11.9 Hz, 1H), 4.14 – 3.95 (m, 1H), 2.10 (s, 3H), 1.22 (d, *J* = 6.3 Hz, 3H). 13C NMR (75 MHz, $CDCl_3$) δ 170.5, 137.9, 129.8, 128.4, 127.9, 127.7, 127.7, 93.7, 93.7, 70.9, 70.1, 64.9, 21.1, 17.9.

2.29 Benzeneethyl 4-*O*-acetyl-2,3,6-trideoxy-α-D-erythro-hex-2-enopyranoside (7d)[12]

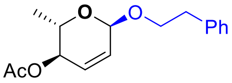

Prepared by the general procedure using **6a** (0.5 mmol, 107 mg) and 2-phenylethanol (0.55 mmol, 66 µL). Column chromatography purification using EtOAc/hexane (1:9) gave **7d** as colourless liquid (110 mg, 80%). ^{1}H NMR (300 MHz, $CDCl_3$) δ 7.39 – 7.19 (m, 5H), 5.95 – 5.75 (m, 2H), 5.05 (dd, *J* = 9.2, 1.5 Hz, 1H), 4.97 (s, 1H), 4.01 (dt, *J* = 9.7, 7.2 Hz, 1H), 3.89 (dd, *J* = 9.2, 6.3 Hz, 1H), 3.76 (dt, *J* = 9.7, 7.0 Hz, 1H), 2.95 (t, *J* = 7.1 Hz, 2H), 2.10 (s, 3H), 1.18 (d, *J* = 6.3 Hz, 3H).

2.30 Benzenebutyl 4-*O*-acetyl-2,3,6-trideoxy-α-D-erythro-hex-2-enopyranoside (7e)

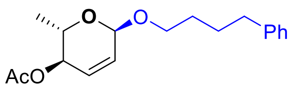

Prepared by the general procedure using **6a** (0.5 mmol, 107 mg) and 4-phenylbutanol (0.55 mmol, 84 µL). Column chromatography purification using EtOAc/hexane (1:9) gave **7e** as colourless liquid (120 mg, 79%). $[\alpha]_D^{20}$ -66.564 (c, 1.2, $CHCl_3$). ^{1}H NMR (300 MHz, $CDCl_3$) δ 7.29 (dd, *J* = 6.0, 0.9 Hz, 2H), 7.20 (dd, *J* = 5.2, 2.4 Hz, 3H), 5.90 – 5.73 (m, 2H), 5.05 (d, *J* = 1.4 Hz, 1H), 4.96 (s, 1H), 4.07 – 3.90 (m, 1H), 3.88 – 3.72 (m, 1H), 3.60 – 3.45 (m, 1H), 2.75 – 2.56 (m, 2H), 2.10 (s, 3H), 1.70 (m, 4H), 1.23 (d, *J* = 6.3 Hz, 3H). ^{13}C NMR (75 MHz, $CDCl_3$) δ 170.5, 162.6, 142.3, 129.5, 128.4, 128.3, 127.9, 125.7, 94.4, 70.9, 68.5, 64.7, 35.6, 29.4, 28.0, 21.0, 17.9

2.31 Cyclohexyl 4-*O*-acetyl-2,3,6-trideoxy-α-D-erythro-hex-2-enopyranoside (7f)[13]

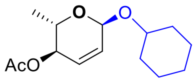

Prepared by the general procedure using **6a** (0.5 mmol, 107 mg) and cyclohexanol (0.55 mmol, 57 µL). Column chromatography purification using EtOAc/hexane (1:9) gave **7f** as colourless liquid (101 mg, 80%). ^{1}H NMR (300 MHz, $CDCl_3$) δ 5.89 – 5.72 (m, 2H), 5.10 (bs, 1H), 5.05 (dd, *J* = 6.7, 2.6 Hz, 1H), 4.03 (dd, *J* = 9.2, 6.3 Hz, 1H), 3.70 – 3.55 (m, 1H), 2.08 (s, 3H), 1.91 (m, 2H), 1.81 – 1.69 (m, 2H), 1.59 – 1.49 (m, 2H), 1.47 – 1.30 (m, 4H), 1.22 (d, *J* = 6.3 Hz, 3H). ^{13}C NMR (75 MHz, $CDCl_3$) δ 170.59, 152.93, 129.2, 128.5, 92.6, 76.4, 71.0, 64.5, 33.9, 32.2, 25.6, 24.3, 24.1, 21.0, 17.9.

2.32 4-*O*-acetyl-2,3,6-trideoxy-D-threo-hex-2-enopyranoside-(α1-6)-1,2:3,4-di-*O*-isopropylidene-D-galactopyranoside (7g)[6]

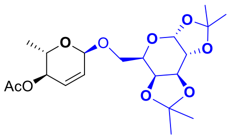

Prepared by the general procedure using **6a** (0.5 mmol, 107 mg) and 1,2:3,4-di-*O*-isopropylidene-α-D-galactopyranose (0.55 mmol, 143 mg). Column chromatography purification using EtOAc/hexane (1:9) gave **7g** as colourless liquid (107 mg, 52%). ^{1}H NMR (300 MHz, $CDCl_3$) δ 5.85 (s, 2H), 5.56 (d, *J* = 5.0 Hz, 1H), 5.07 (d, *J* = 7.8 Hz, 2H), 4.62 (dd, *J* = 7.9, 2.3 Hz, 1H), 4.31 (ddd, *J* = 9.4, 6.5, 1.9 Hz, 2H), 4.06 – 3.92 (m, 2H), 3.77 – 3.62 (m, 1H), 2.10 (s, 3H), 1.56 (s, 3H), 1.47 (s, 3H), 1.36 (s, 6H), 1.24 (d, *J* = 6.3 Hz, 3H). ^{13}C NMR (75 MHz, $CDCl_3$) δ 170.5, 129.5, 127.9, 109.2, 108.5, 96.3, 94.2, 71.13, 71.0, 70.6, 70.5, 67.1, 66.5, 64.8, 26.1, 26.0, 24.9, 24.4, 21.1, 17.8.

2.33 4-Methyl phenyl 4-*O*-acetyl-2,3,6-trideoxy-1-thio-α-D-*erythro*-hex-2-enopyranoside (7h)[14]

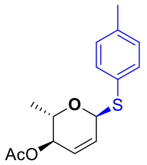

Prepared by the general procedure using **6a** (0.5 mmol, 107 mg) and 4-methylbenzenethiol (0.55 mmol, 68 mg). Column chromatography purification using EtOAc/hexane (1:9) gave **7h** as colourless liquid (122 mg, 88%). ^{1}H NMR (300 MHz, $CDCl_3$) δ 7.44 (d, *J* = 8.1 Hz, 2H), 7.15 (d, *J* = 8.1 Hz, 2H), 6.05 (ddd, *J* = 10.1, 2.8, 2.0 Hz, 1H), 5.84 (dd, *J* = 10.1, 1.5 Hz, 1H), 5.66 (s, 1H), 5.17 (ddd, *J* = 9.0, 3.7, 1.8 Hz, 1H), 4.40 – 4.19 (m, 1H), 2.36 (s, 3H), 2.13 (s, 3H), 1.30 (d, *J* = 6.3 Hz, 3H). ^{13}C NMR (75 MHz, $CDCl_3$) δ 170.5, 137.5, 132.0, 129.7, 128.6, 127.9, 84.0, 70.7, 65.35, 21.1, 18.0.

References

1. J. A. M. Santos, C. S. Santos, C. L.A. Almeida, T. D.S. Silva, J. R. F. Filho, G. C.G. Militao, T. G. da Silva, C. H.B. da Cruz, J. C.R. Freitas, P. H. Menezes, *Eur. J. Org. Chem*., **2017**, *128*, 192-201.
2. A. T. Khan, R. S. Basha, M. Lal, *Arkivoc*, **2013** (ii), 201-212.
3. S. Jung, A. Inoue, S. Nakamura, T. Kishi, A. Uwamizu, M. Sayama, M. Ikubo, Y. Otani, K. Kano, K. Makide, J. Aoki, T. Ohwada, *J. Med. Chem*., **2016**, *59*, 3750−3776.
4. P. Chen, B. Bi, *Tetrahedron Lett*., **2015**, *56*, 4895–4899.
5. J. C. R. Freitas, T. R. Couto, A. A. S. Paulino, J. R. de Freitas Filho, I. Malvestiti, R. A. Oliveira, P. H. Menezes, *Tetrahedron*, **2012**, *68*, 10611-10620.
6. a) A. Sau, M. C. Galan, *Org. Lett*., **2017**, *19*, 2857−2860; b) Chervin,S. M.; Abada, P.; Koreeda, M. *Org. Lett*. **2000**, *2*, 369.
7. B. V. Subba Reddy, C. Divyavani, J. S. Yadav, *Synthesis*, **2010**, *10*, 1617–1620.
8. G. Huang, M. Isobe, *Tetrahedron*, **2001**, *57*, 10241-10246.
9. P. Chen, X. Zhanga, *Tetrahedron Lett*., **2017**, *58*, 309-312.
10. T. R. Reddy, S. Chittel, S. Kashyap, *Tetrahedron*, **2014**, *70*, 9224-9229.
11. P. Gupta, N. Kumari, A. Agarwal, Y. D. Vankar, *Org. Biomol. Chem*., **2008**, *6*, 3948–3956.
12. S. K. Das, K. A. Reddy, J. Roy, *Synlett*, **2003**, *11*, 1607-1610.
13. P. Chen, S. Li, *Tetrahedron Lett*., **2014**, *55*, 5813–5816.
14. D. Stevanovic, A. Pejovic, I. Damljanovic, A. Minic, G. A. Bogdanovic, M. Vukicevic, N. S. Radulovic, R. D. Vukicevic, *Carbohydr. Res*., **2015**, *407*, 111-121.

3. Spectral images of compounds 3a–u, 5a–d and 7a–h

^{1}H NMR of compound 3a

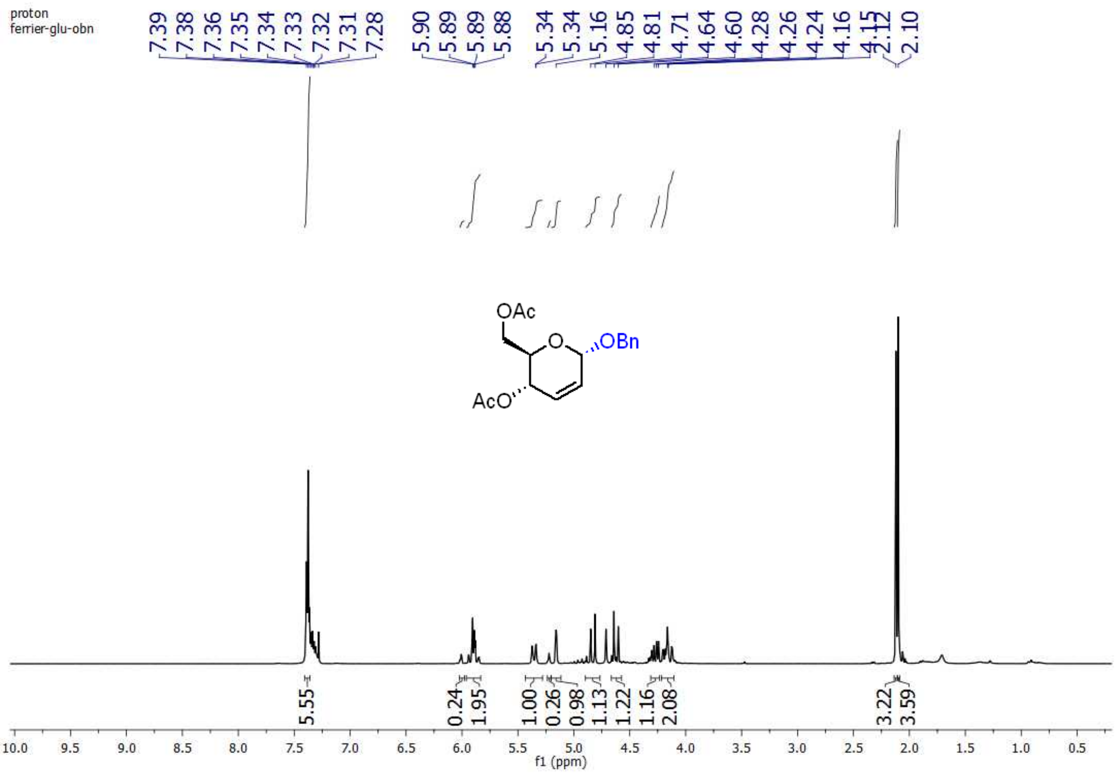

^{13}C NMR of compound 3a

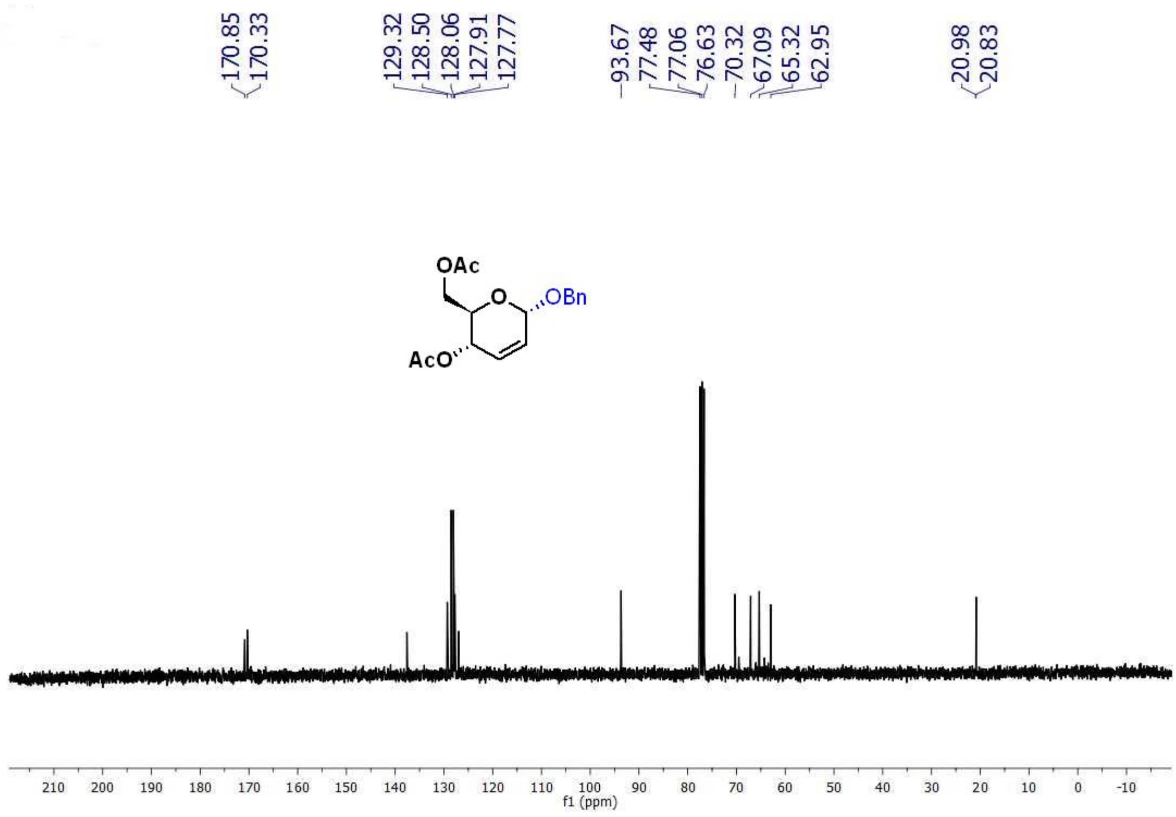

^{1}H NMR of compound 3b

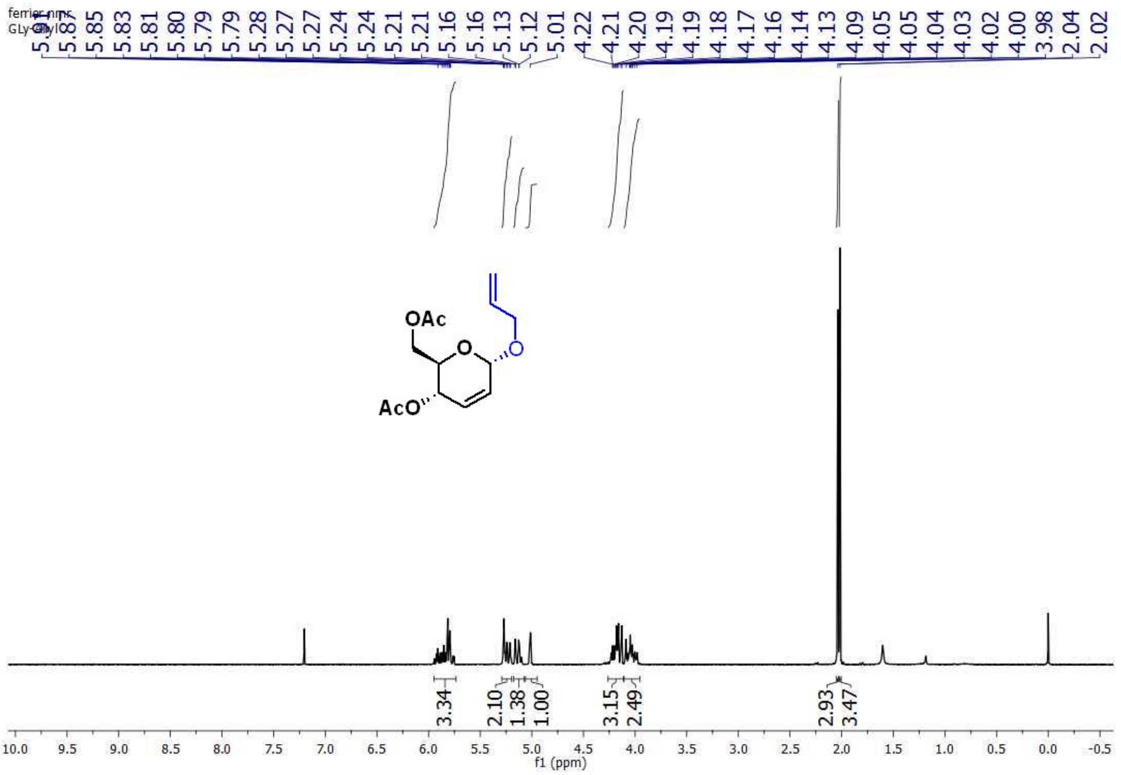

^{13}C NMR of compound 3b

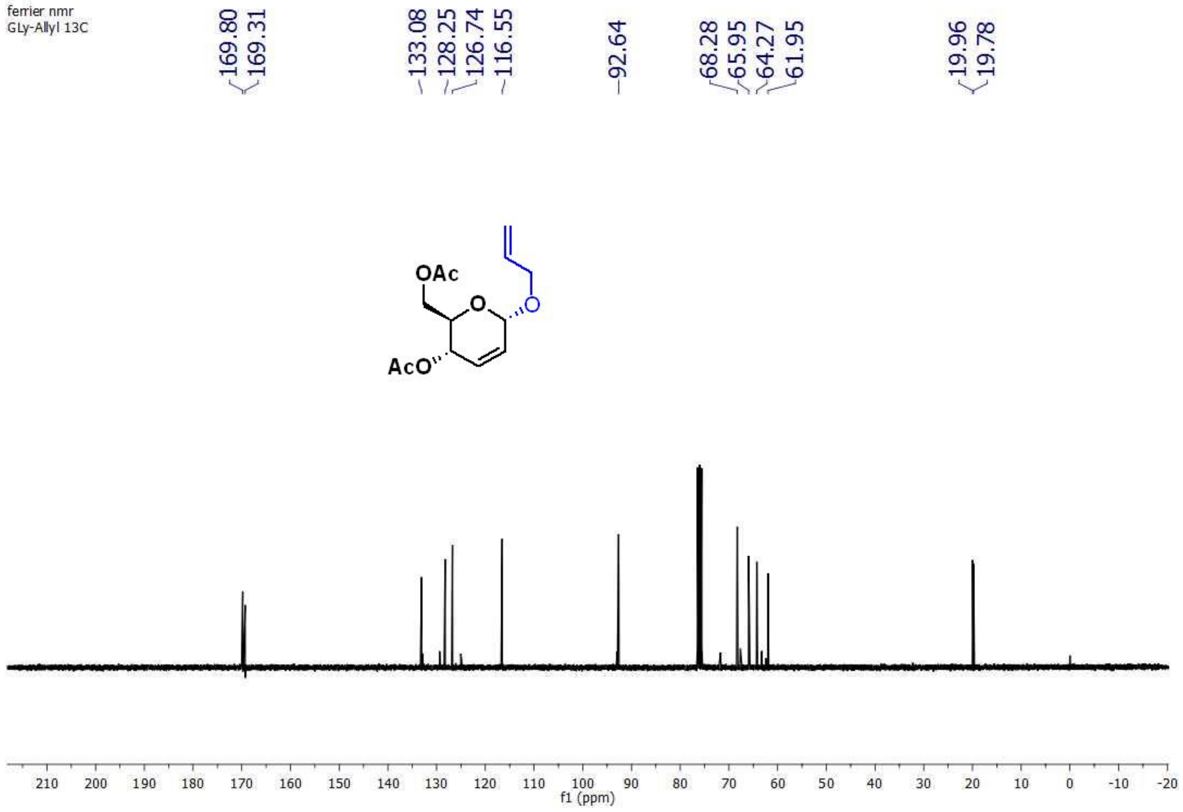

^{1}H NMR of compound 3c

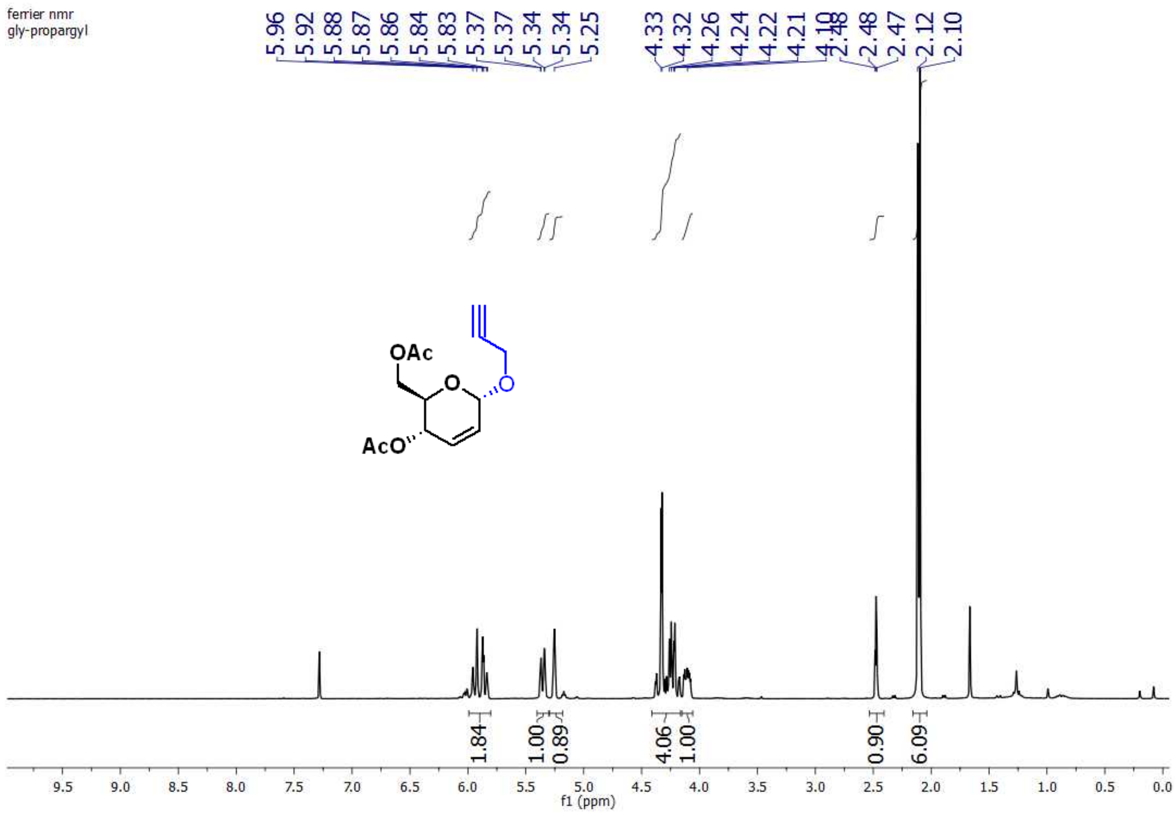

^{13}C NMR of compound 3c

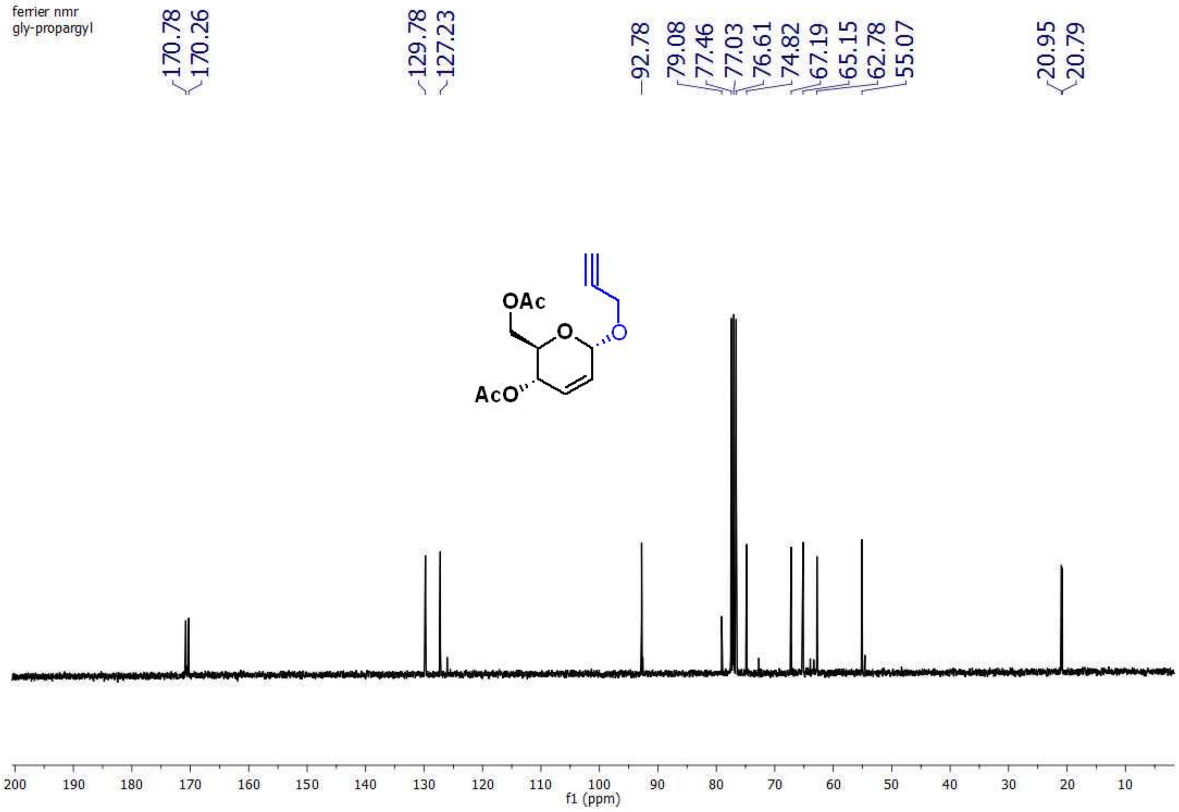

¹H NMR of compound 3d

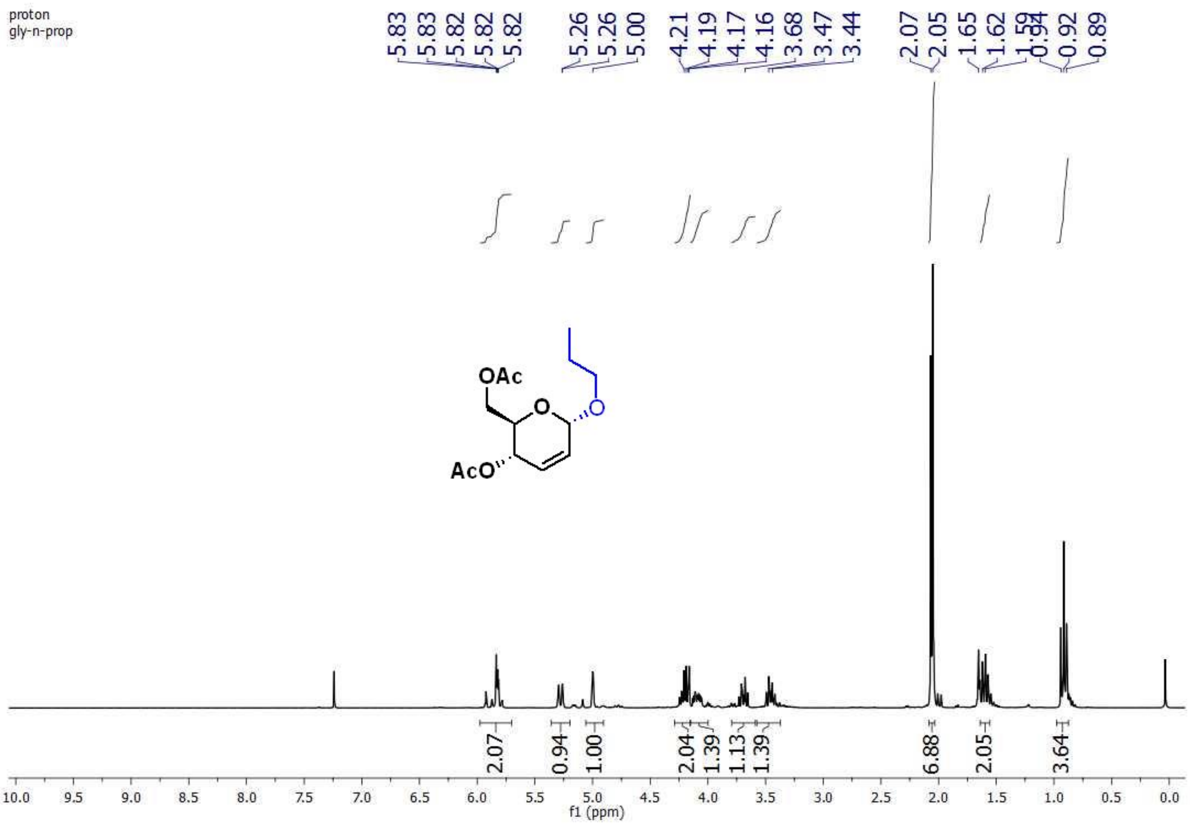

^{1}H NMR of compound 3e

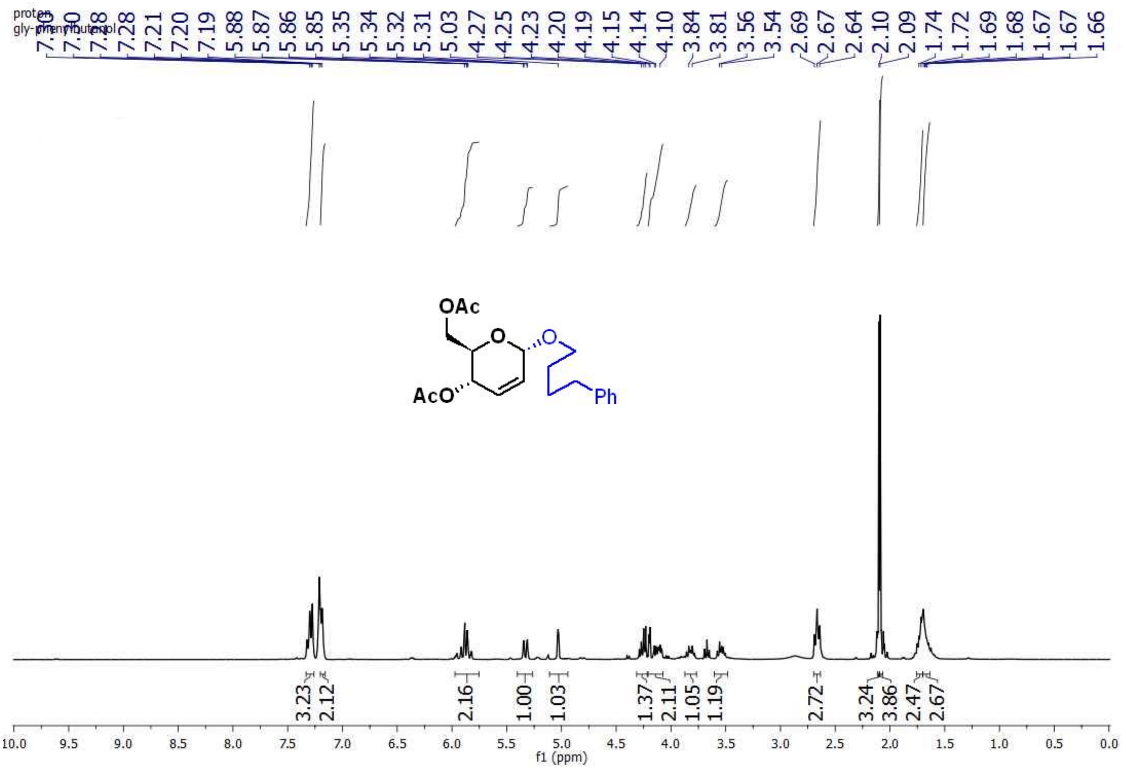

^{13}C NMR of compound 3e

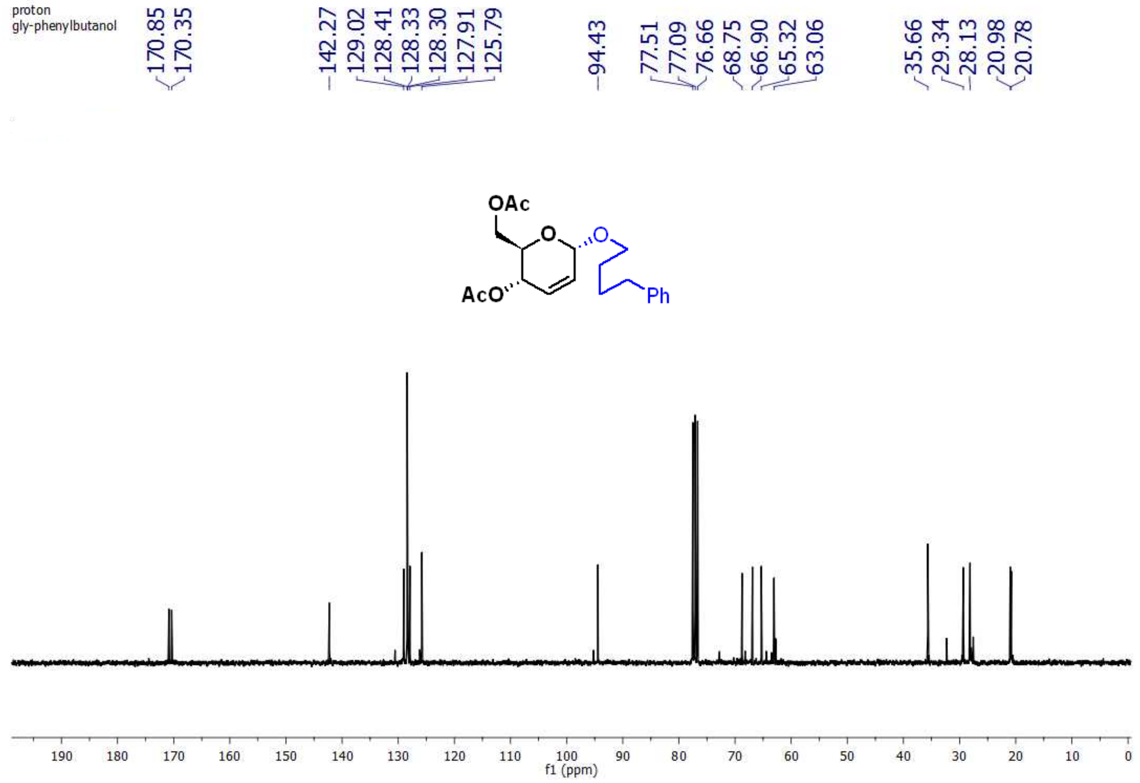

^{1}H NMR of compound 3f

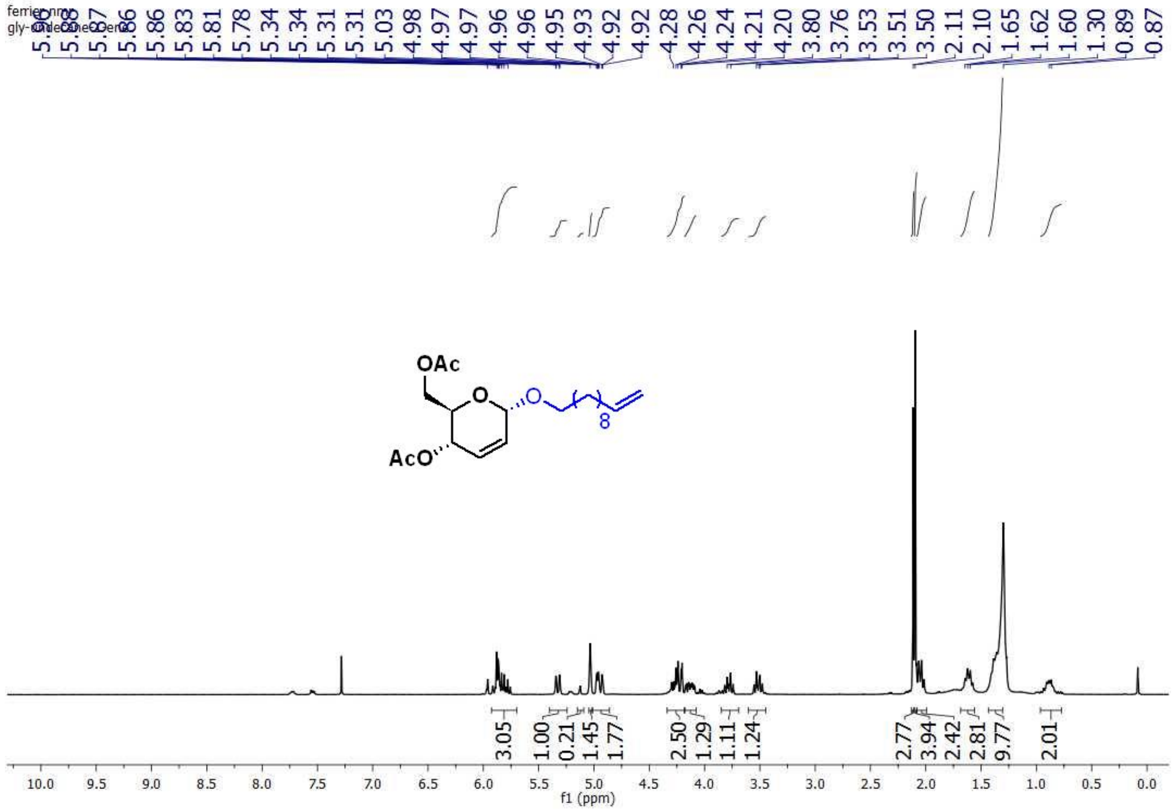

^{13}C NMR of compound 3f

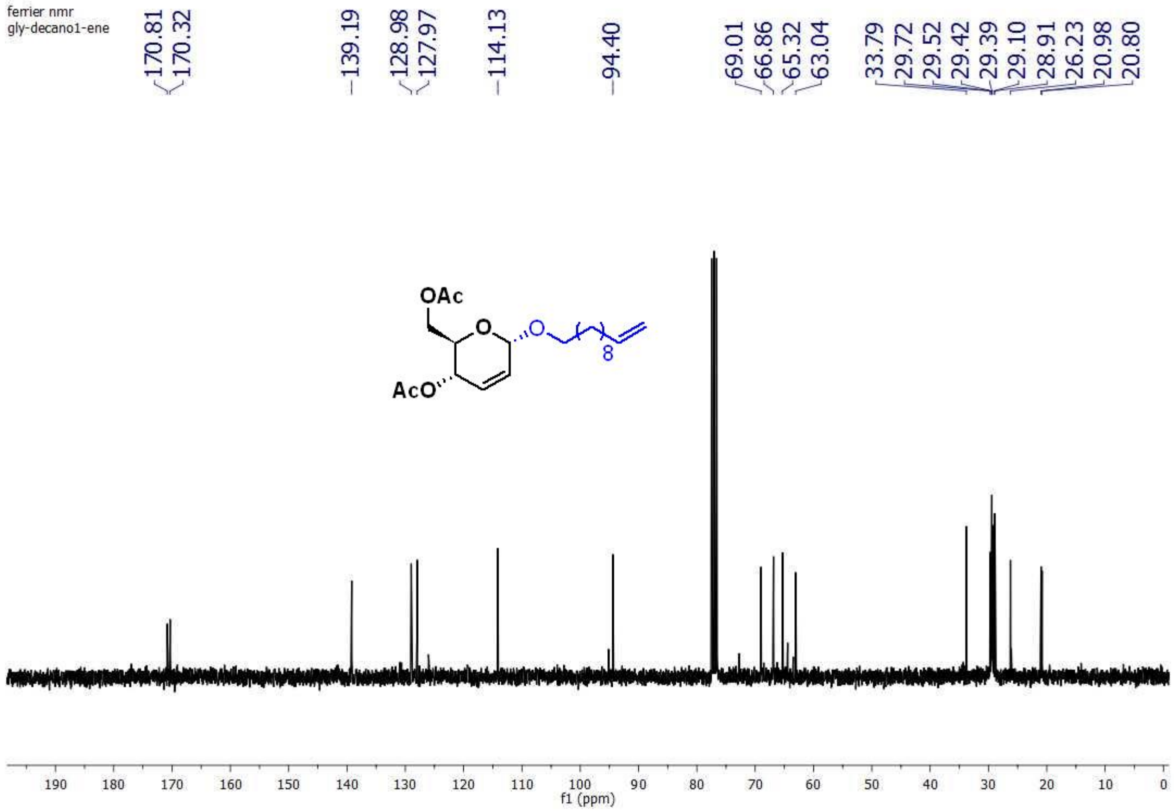

^{1}H NMR of compound 3g

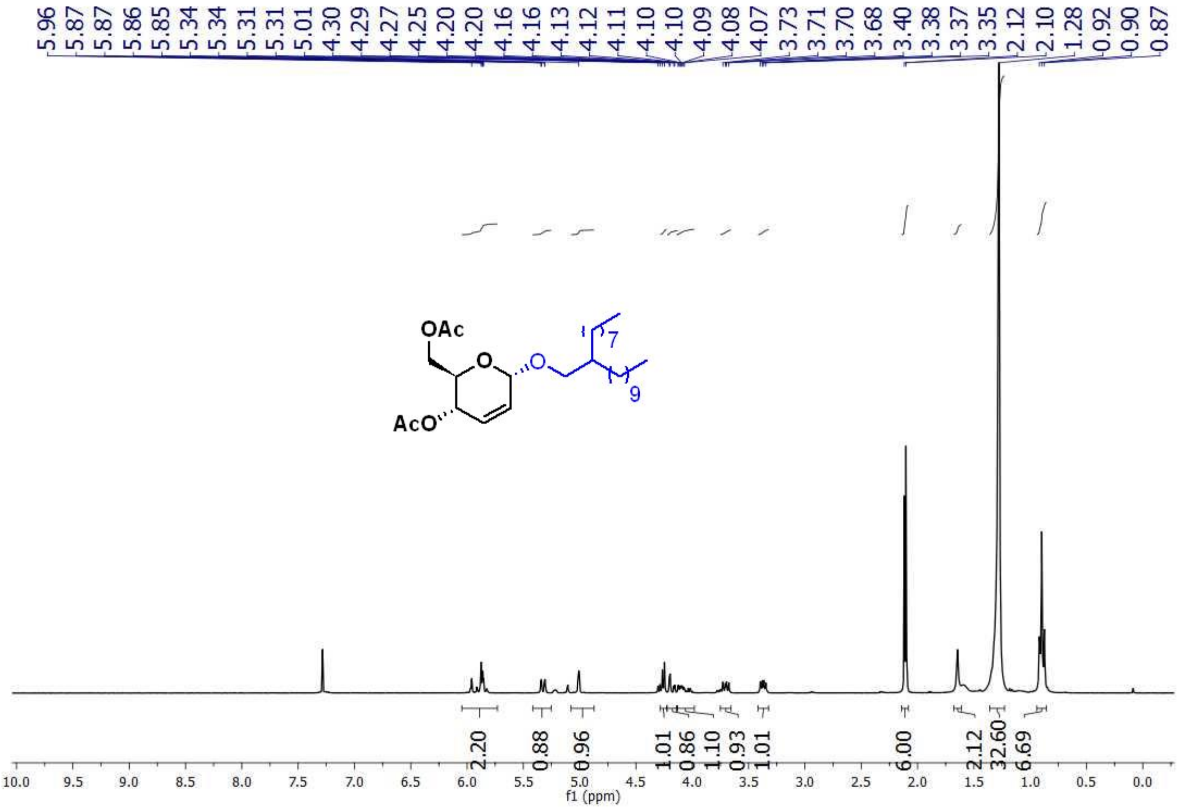

^{13}C NMR of compound 3g

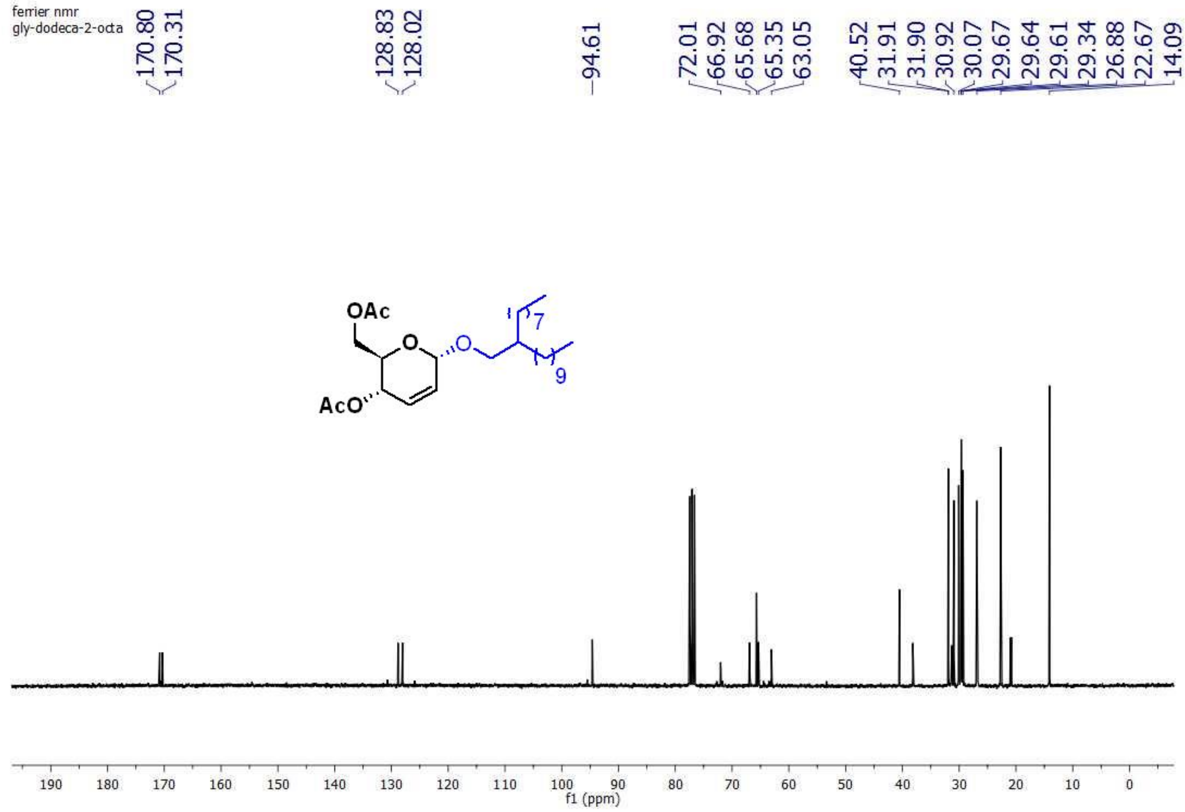

^{1}H NMR of compound 3h

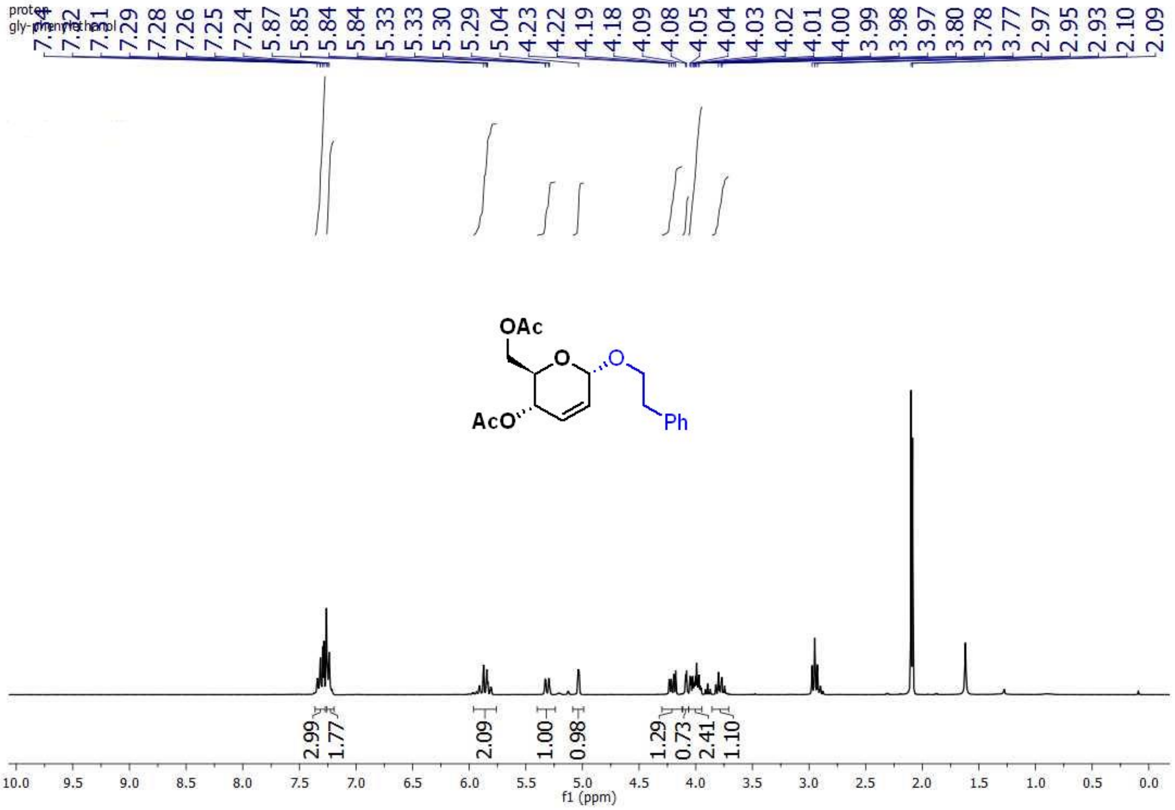

^{13}C NMR of compound 3h

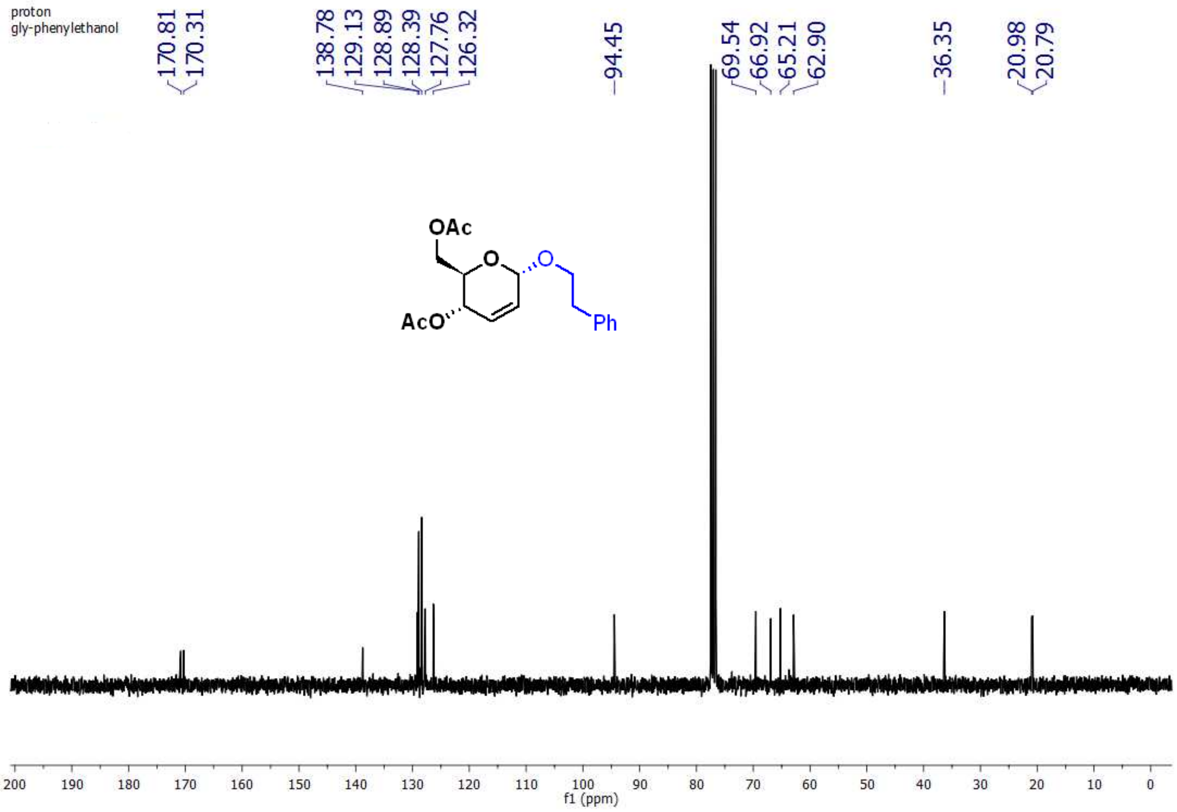

^{1}H NMR of compound 3i

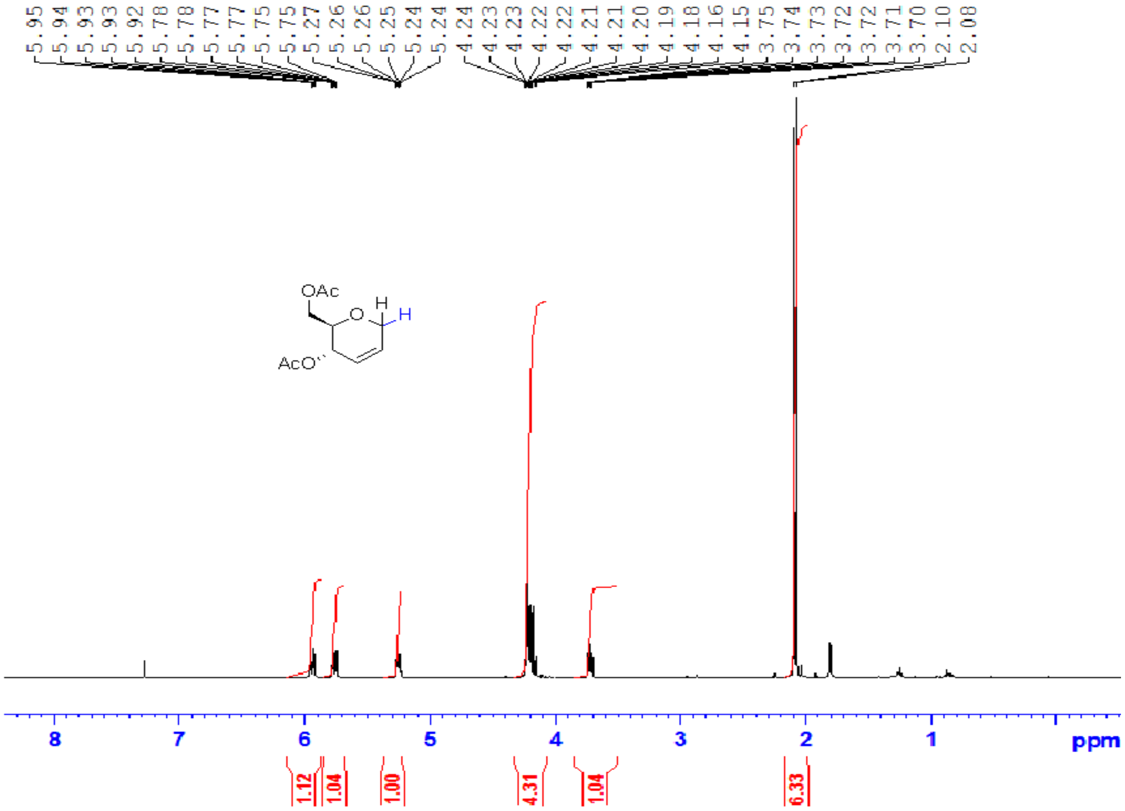

^{13}C NMR of compound 3i

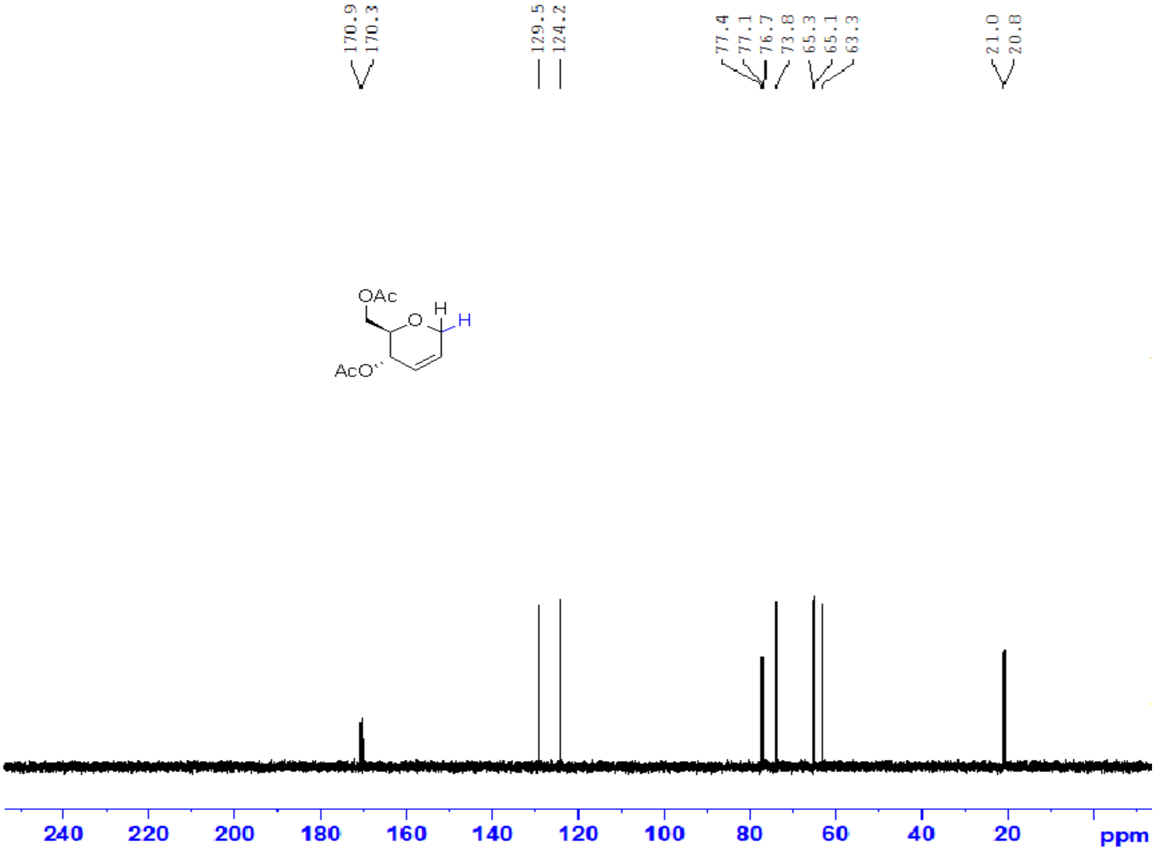

^{1}H NMR of compound 3j

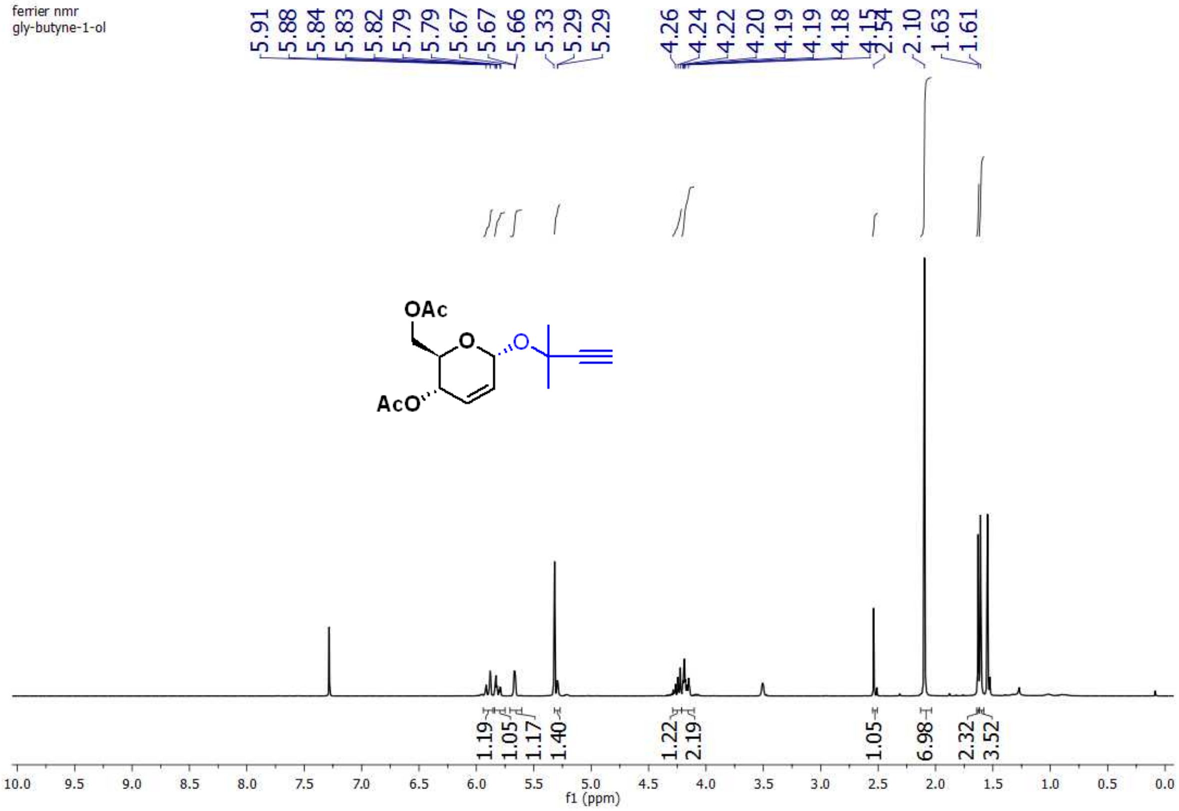

^{13}C NMR of compound 3j

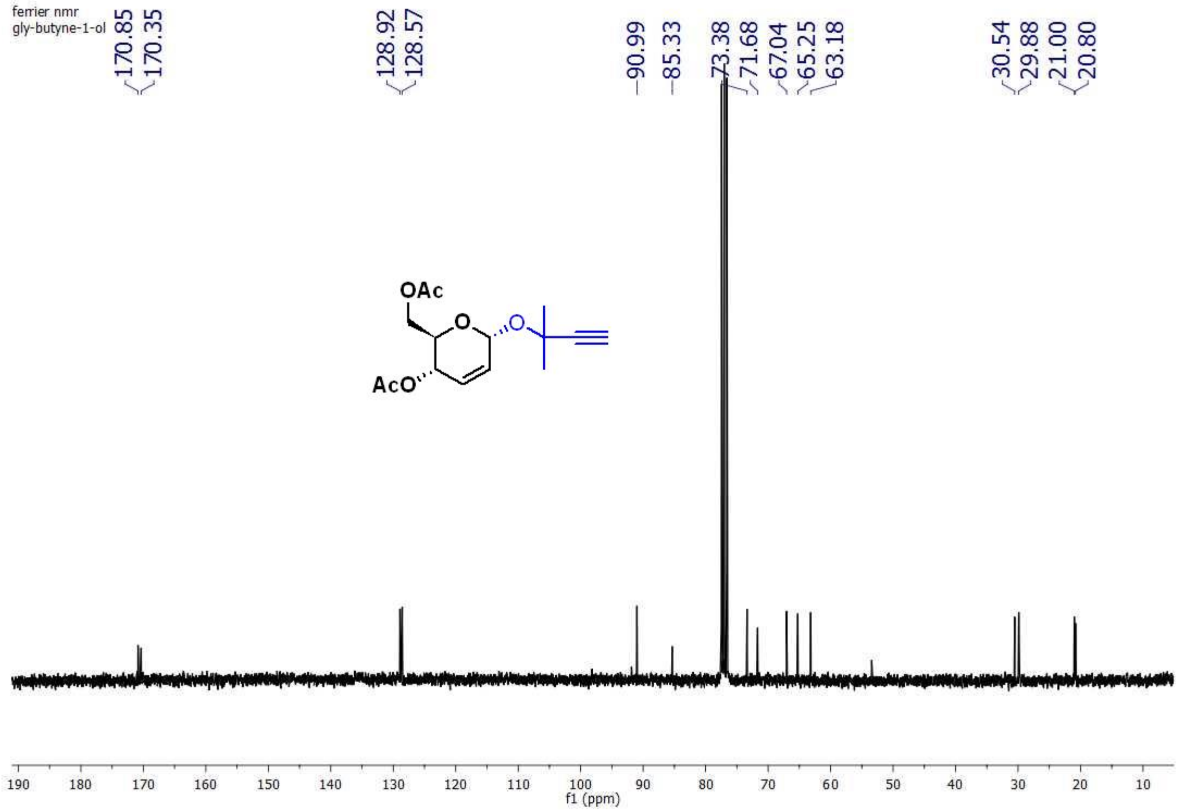

^{1}H NMR of compound 3k

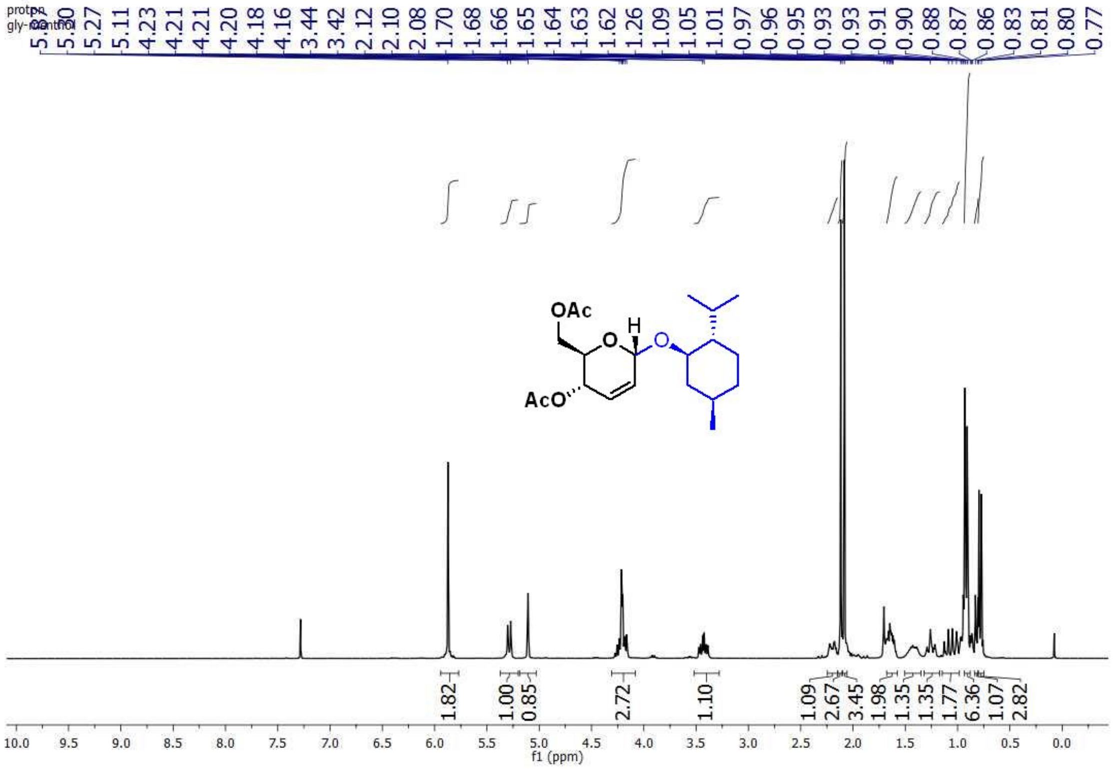

^{13}C NMR of compound 3k

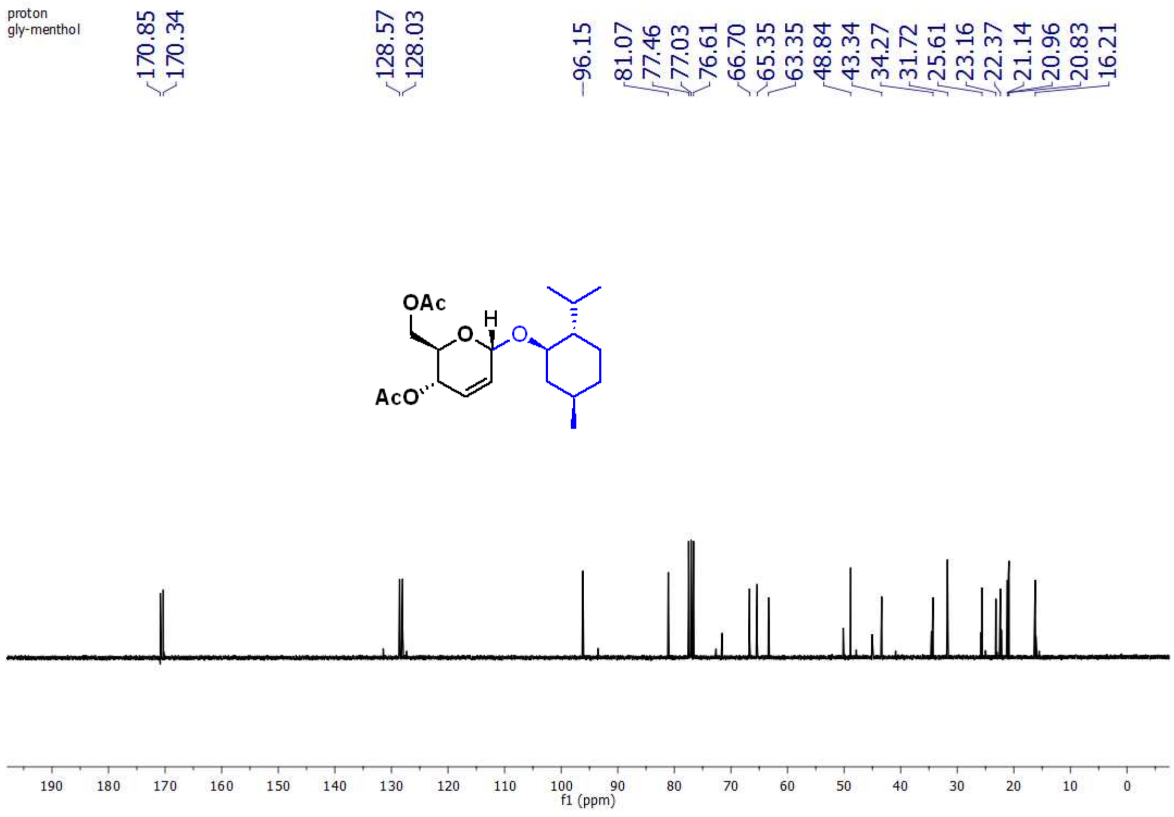

^{1}H NMR of compound 3l

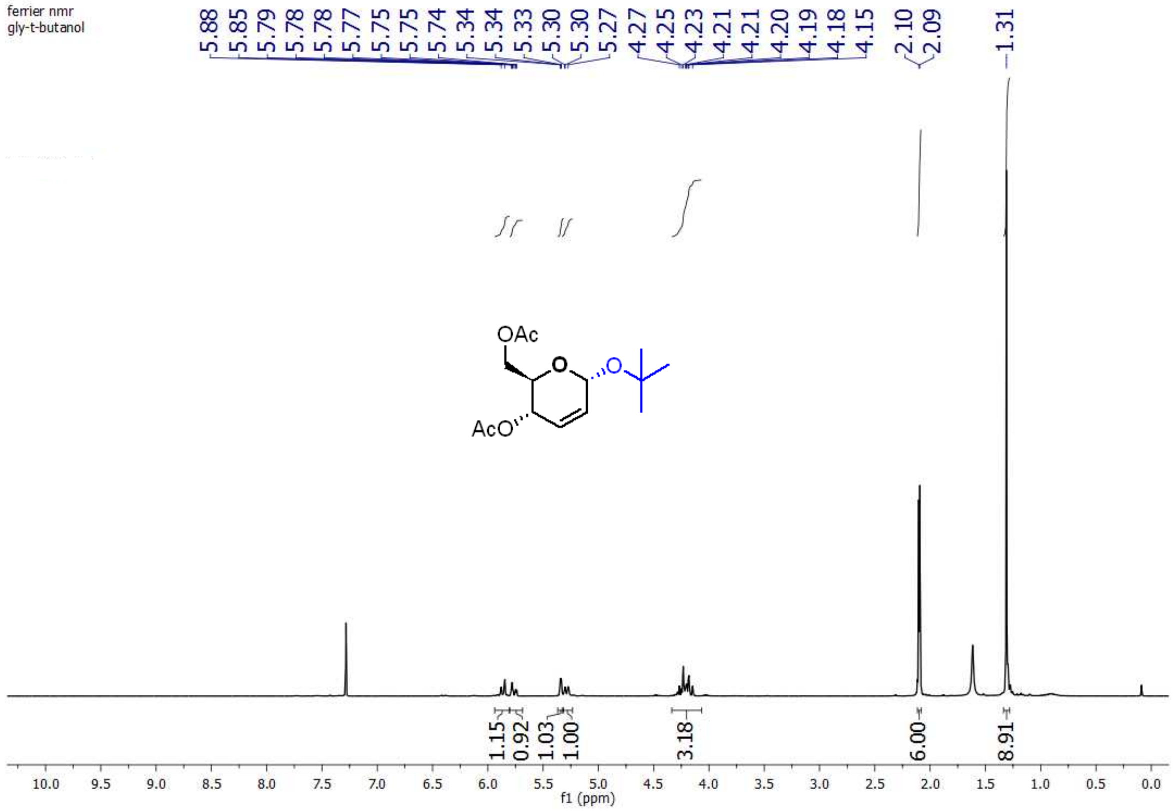

^{13}C NMR of compound 3l

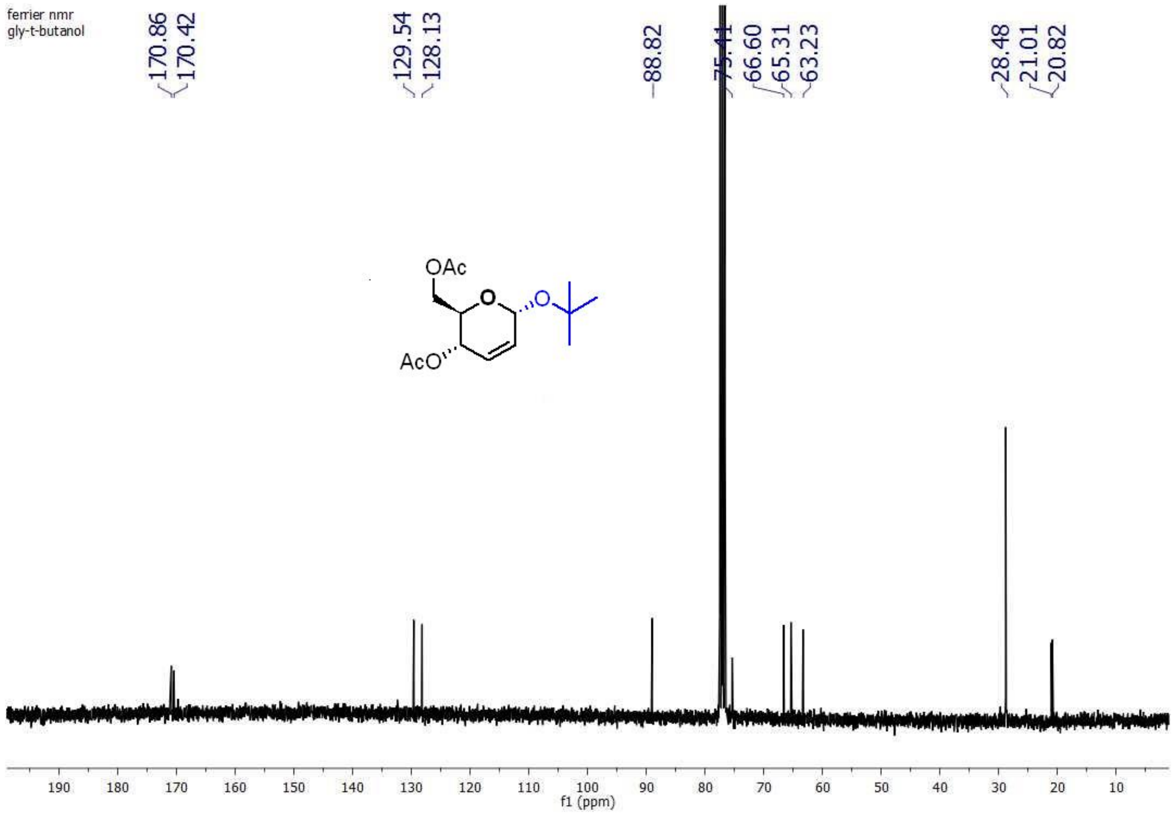

^{1}H NMR of compound 3m

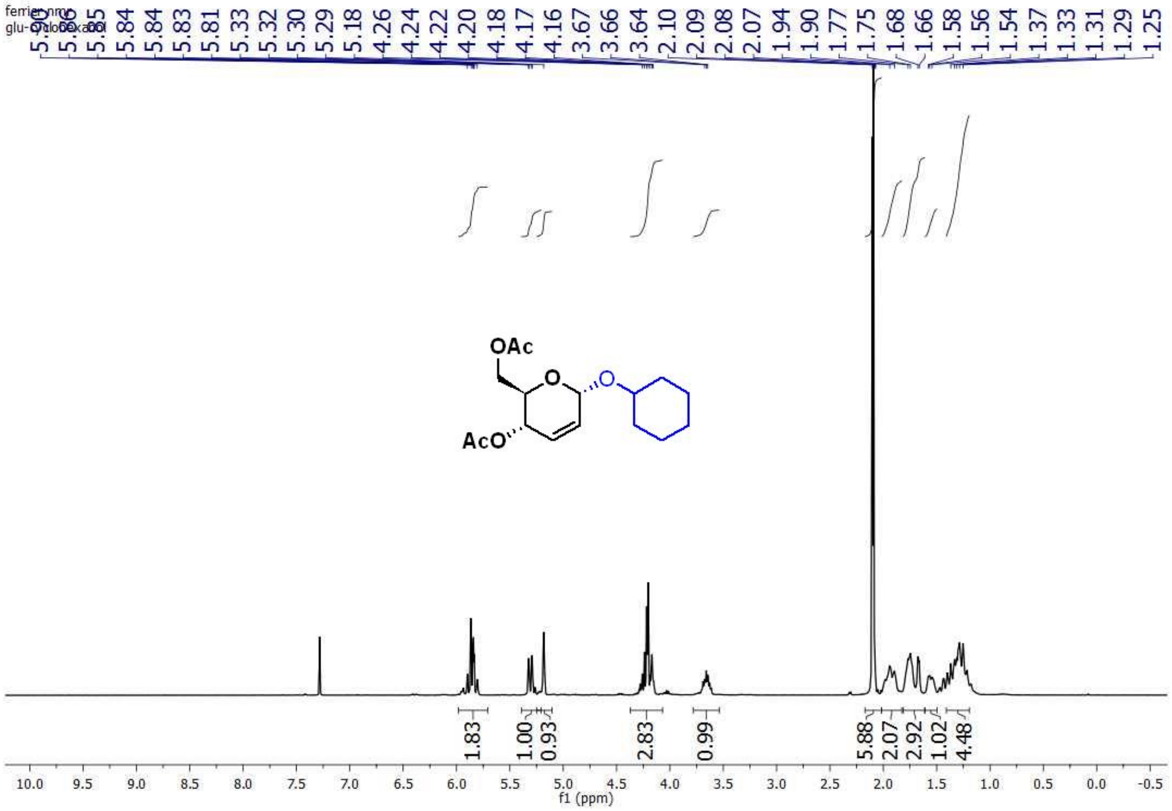

^{13}C NMR of compound 3m

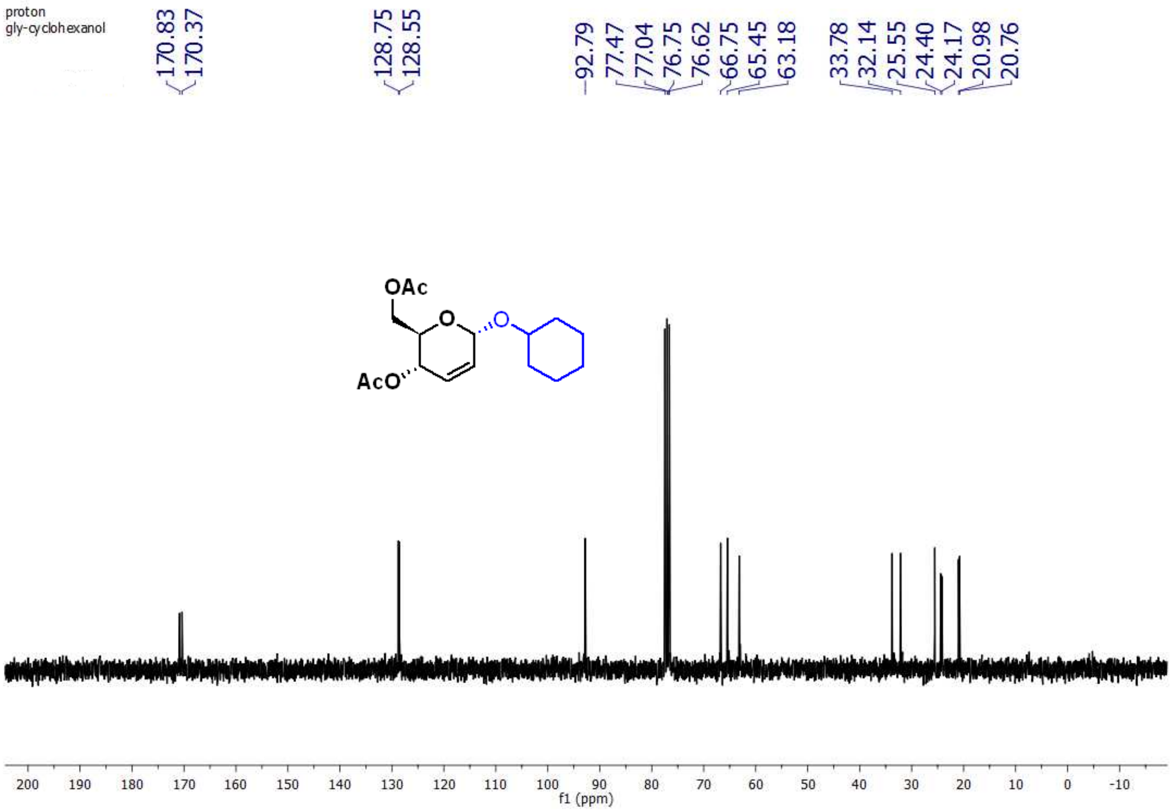

^{1}H NMR of compound 3n

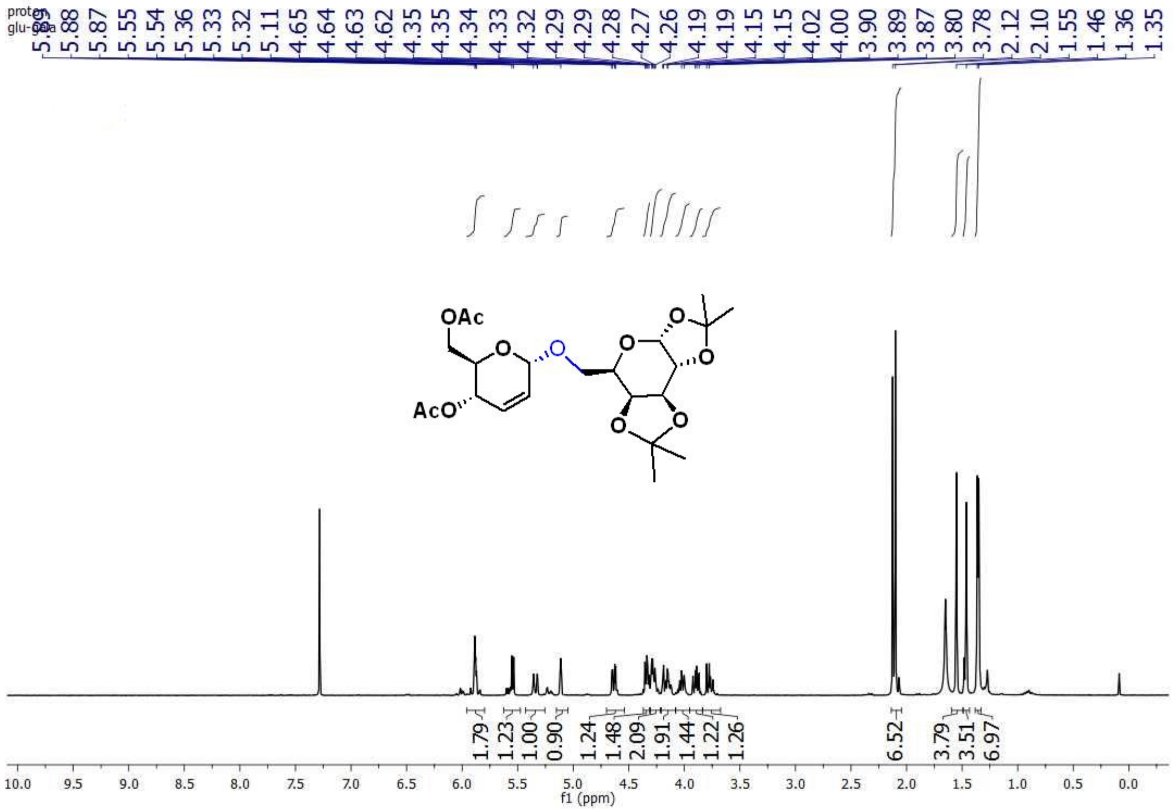

^{13}C NMR of compound 3n

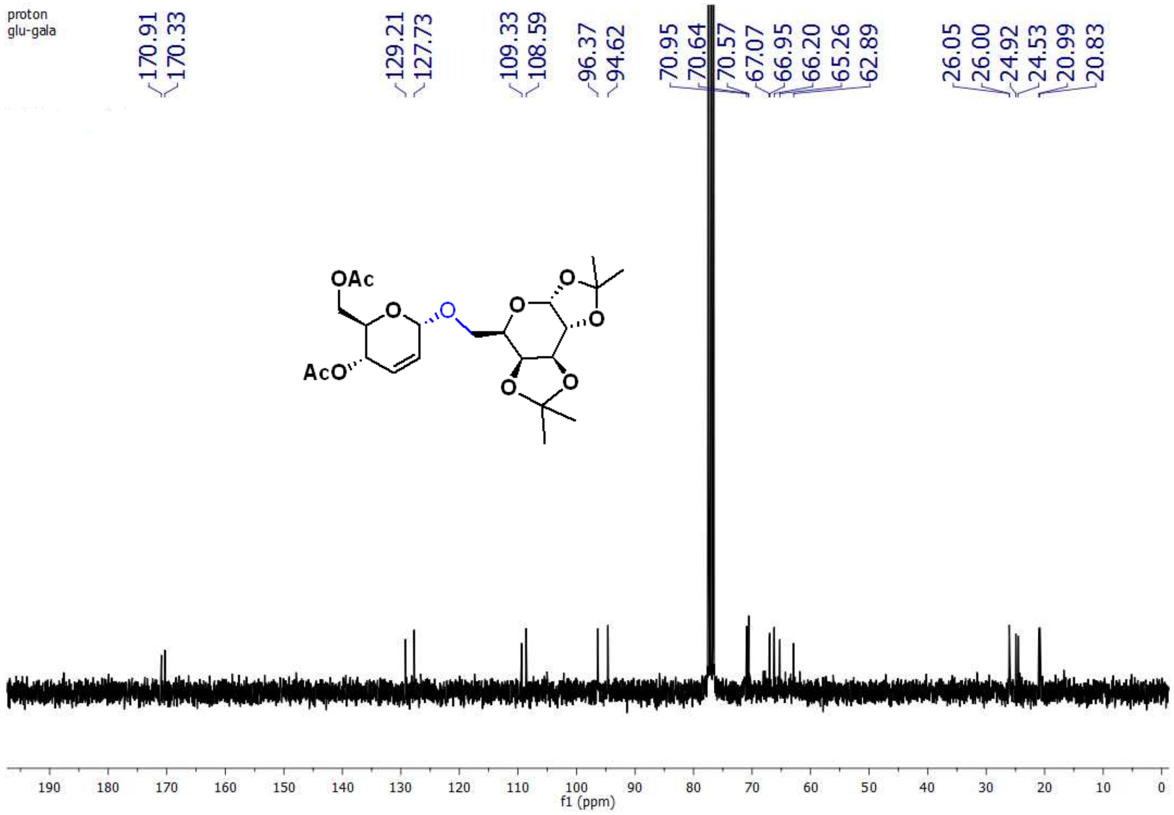

^{1}H NMR of compound 3o

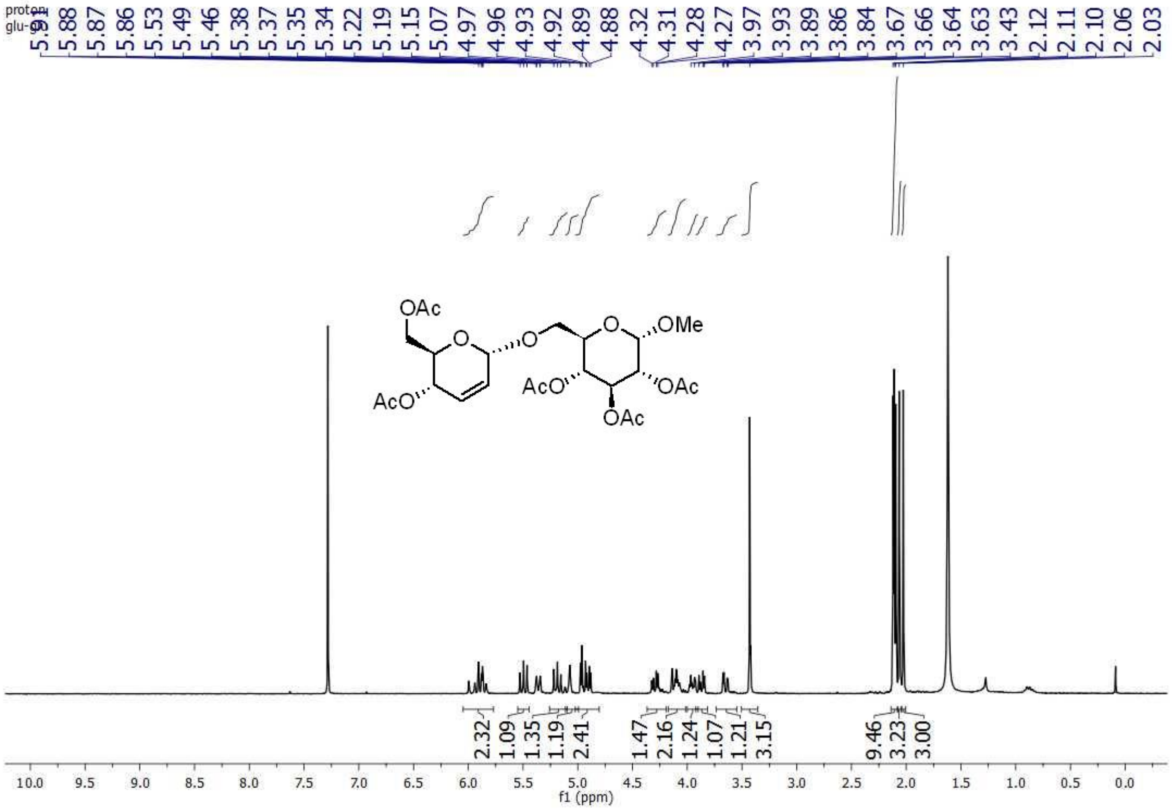

^{1}H NMR of compound 3p

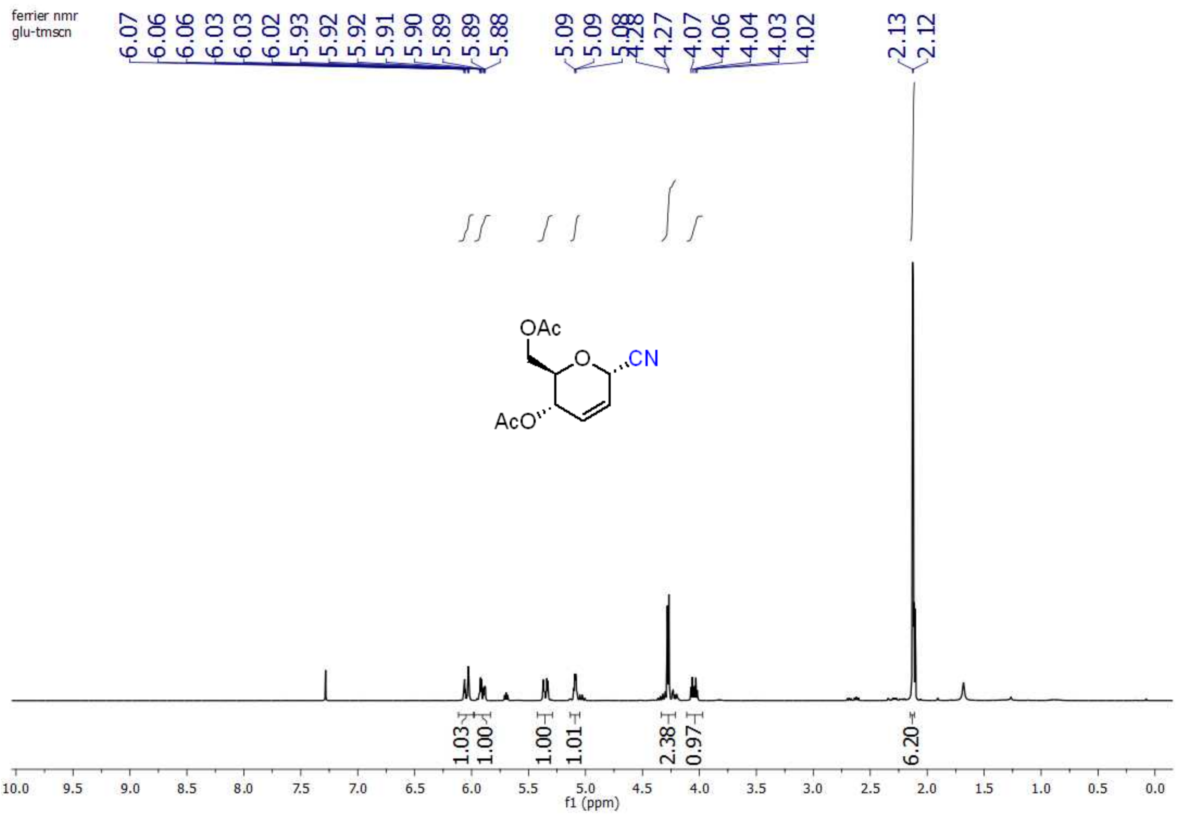

^{13}C NMR of compound 3p

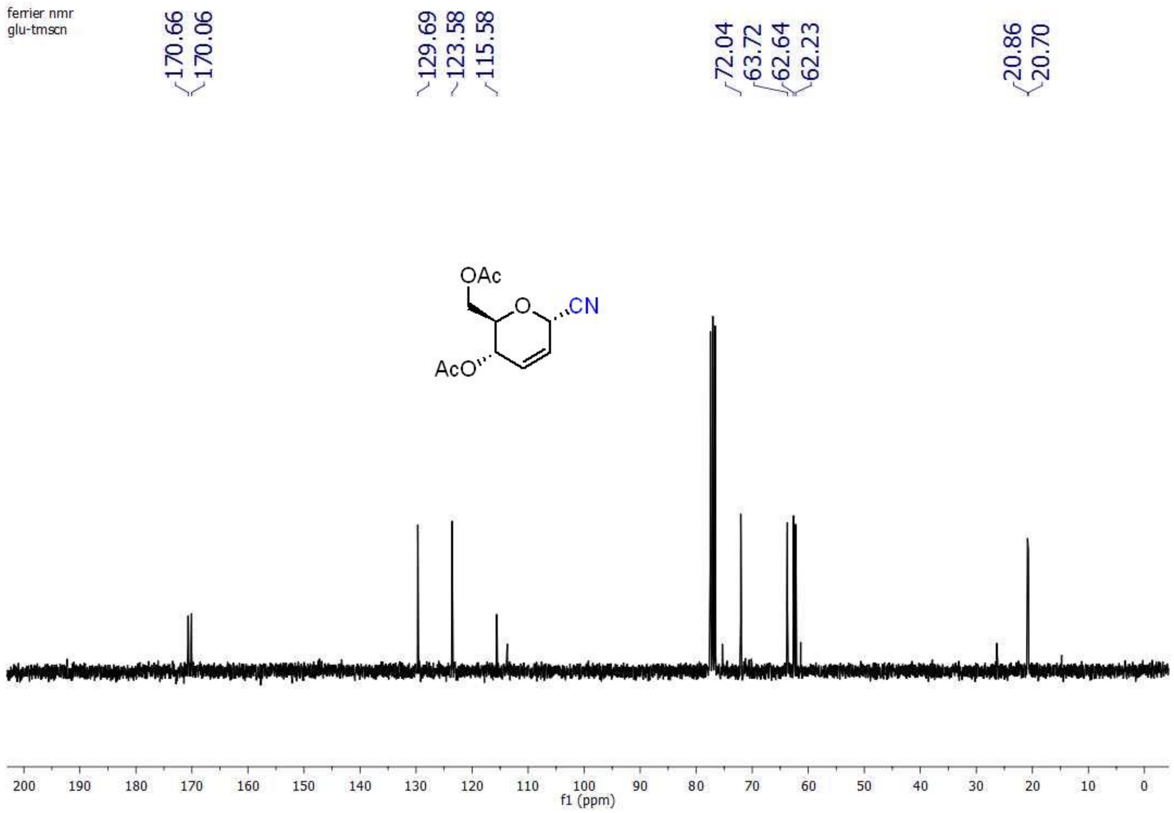

^{1}H NMR of compound 3q

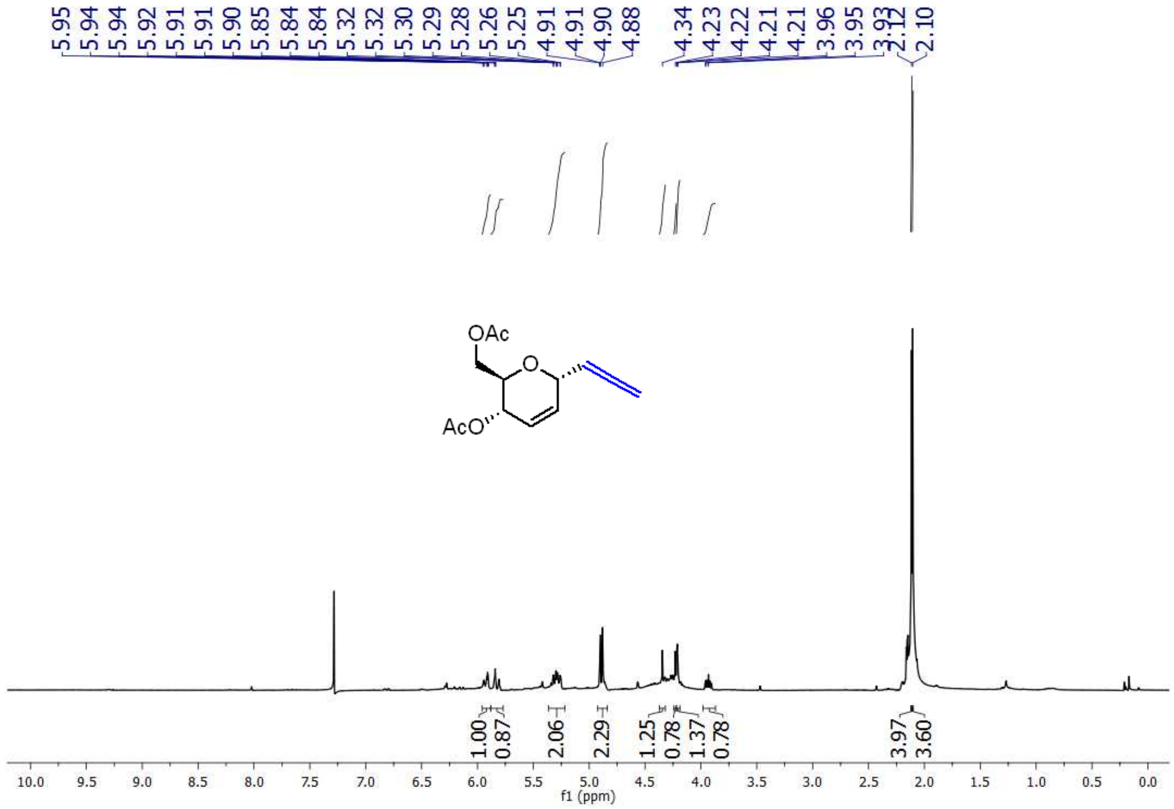

^{13}C NMR of compound 3q

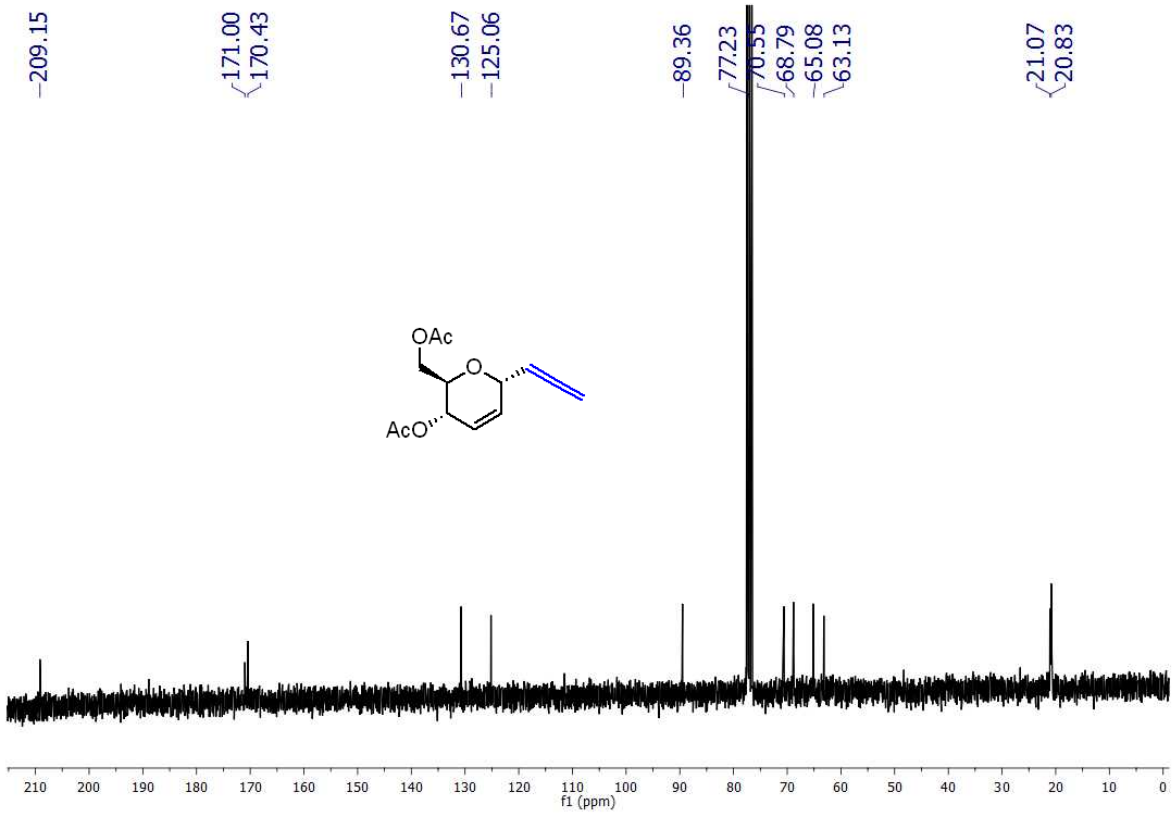

^{1}H NMR of compound 3r

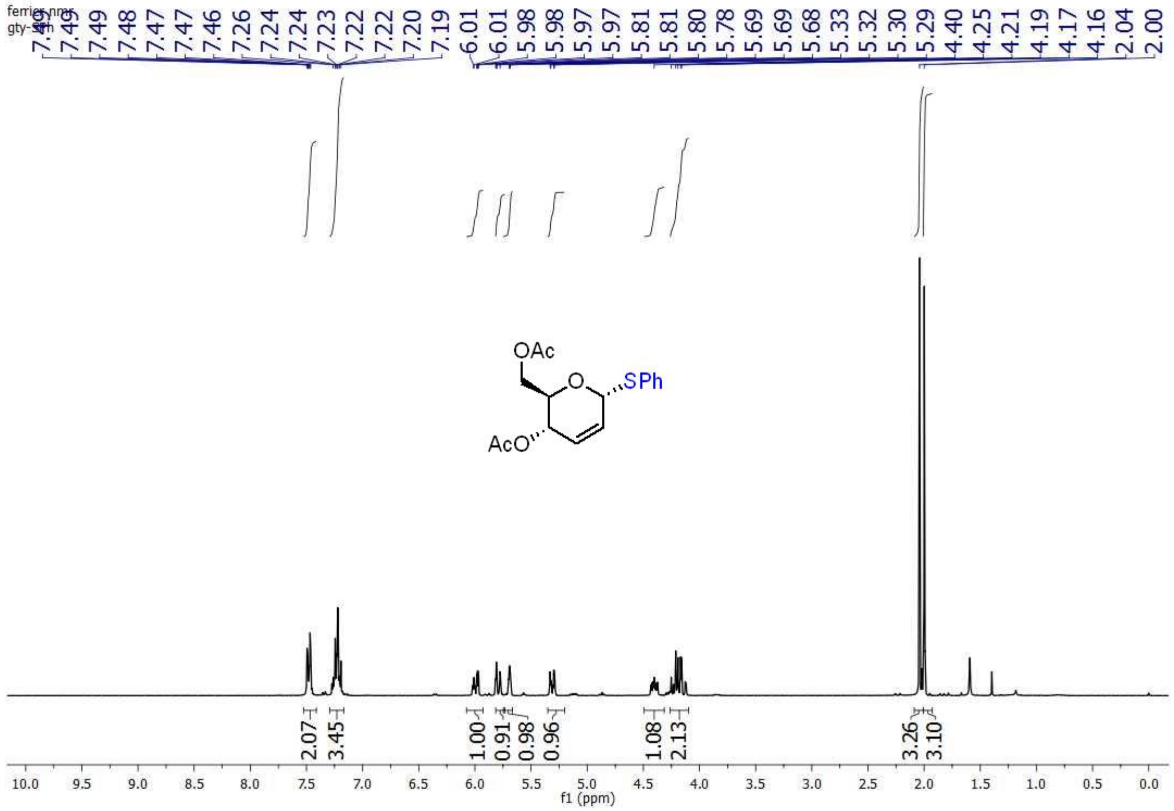

^{13}C NMR of compound 3r

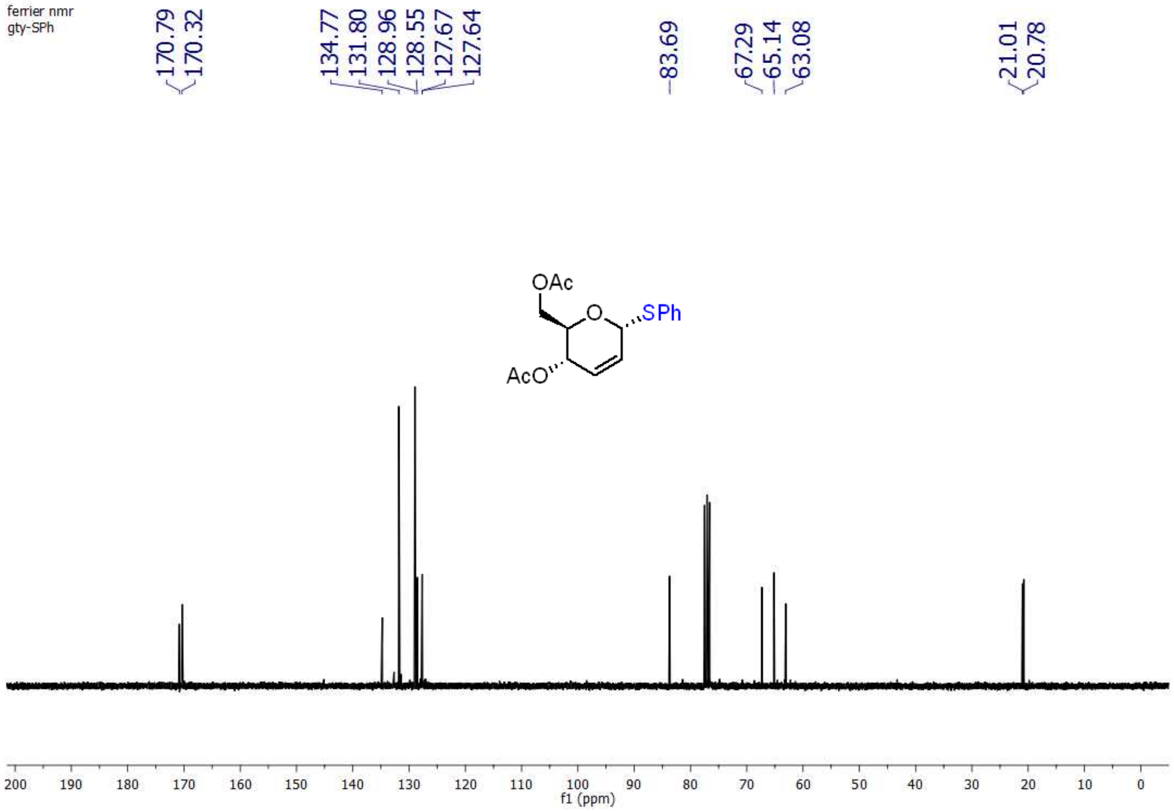

^{1}H NMR of compound 3s

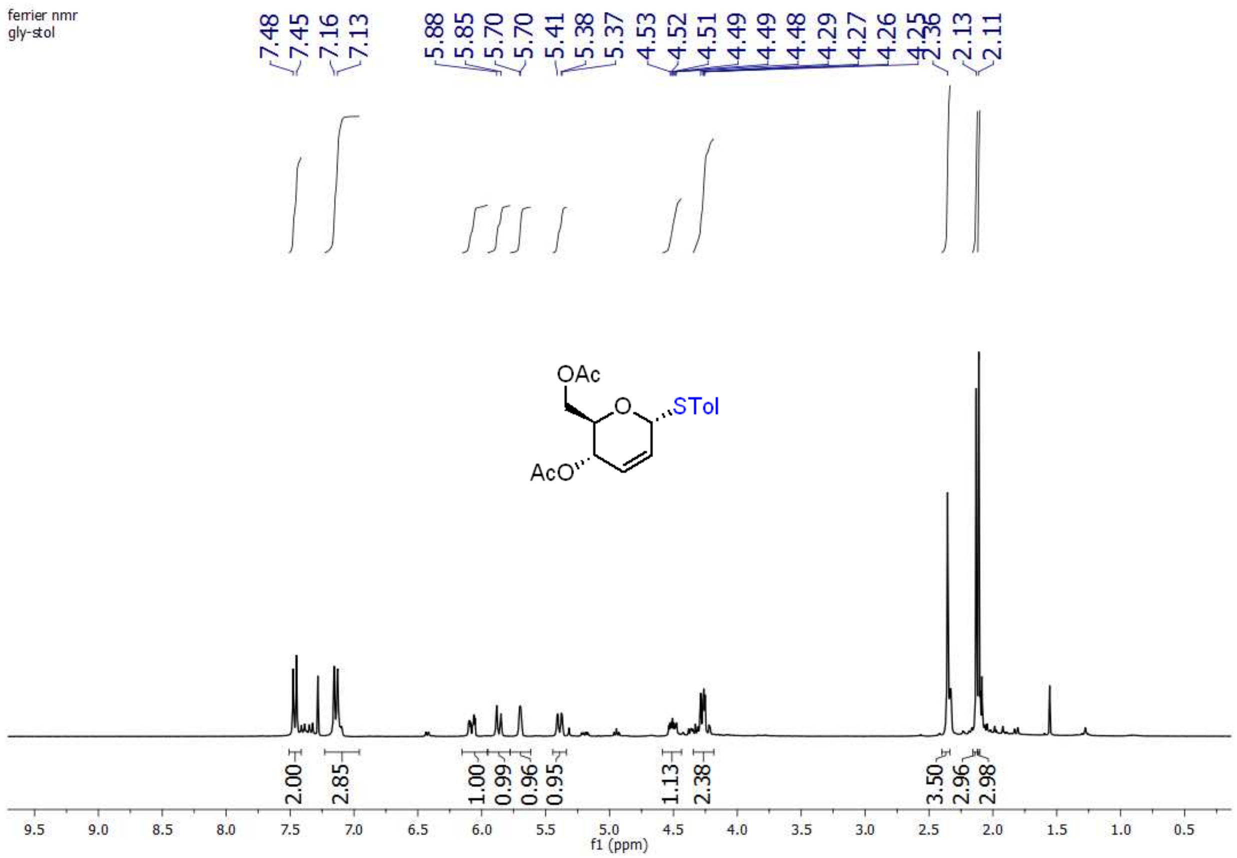

^{1}H NMR of compound 3t

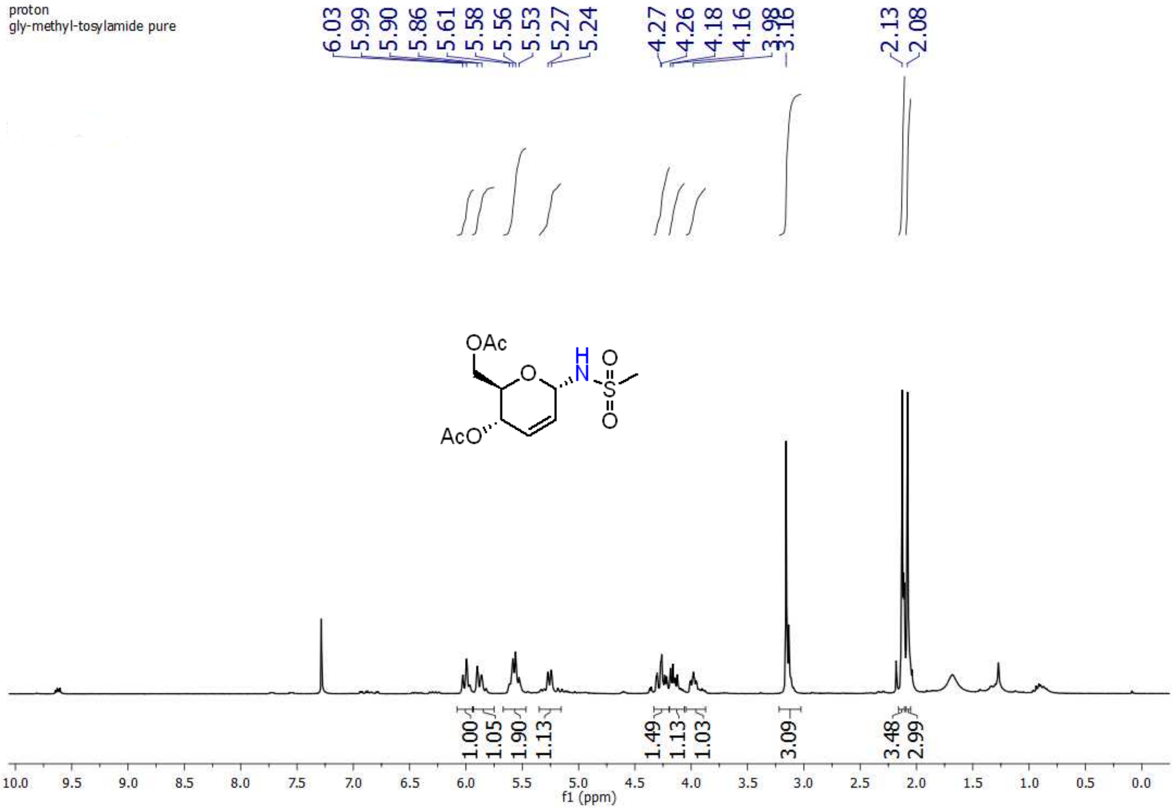

^{13}C NMR of compound 3t

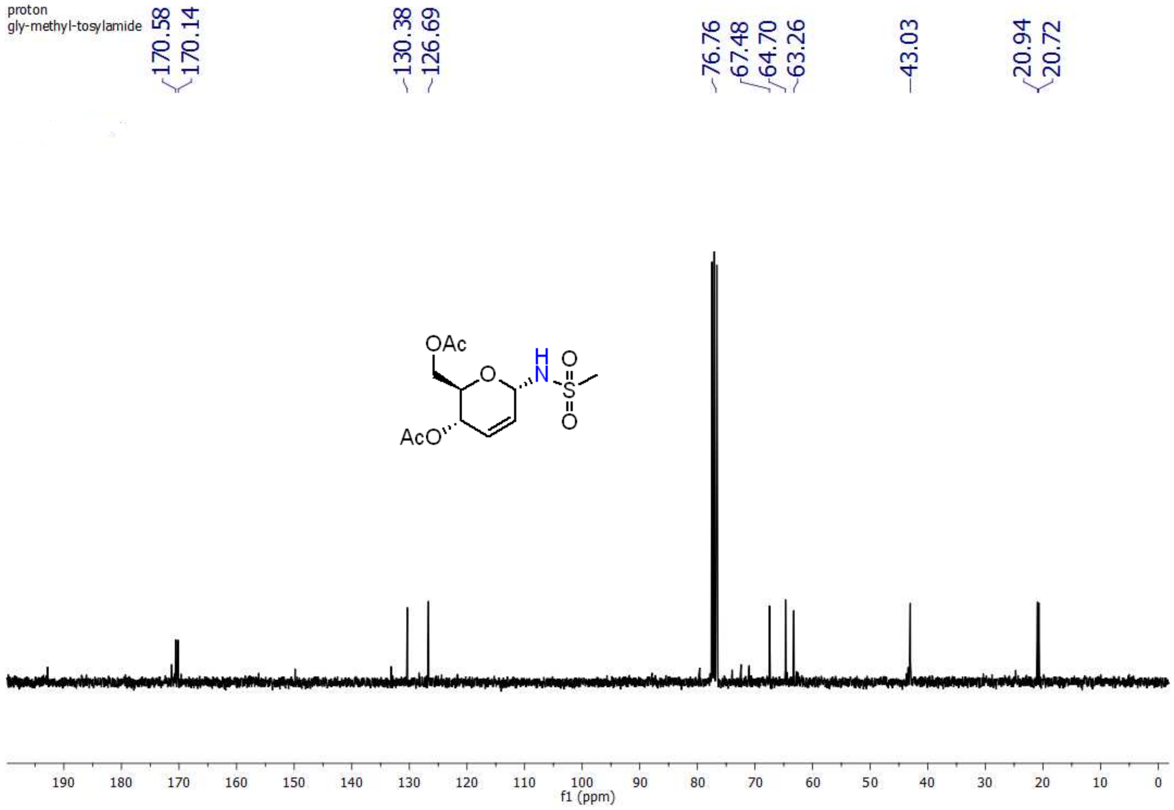

^{1}H NMR of compound 3u

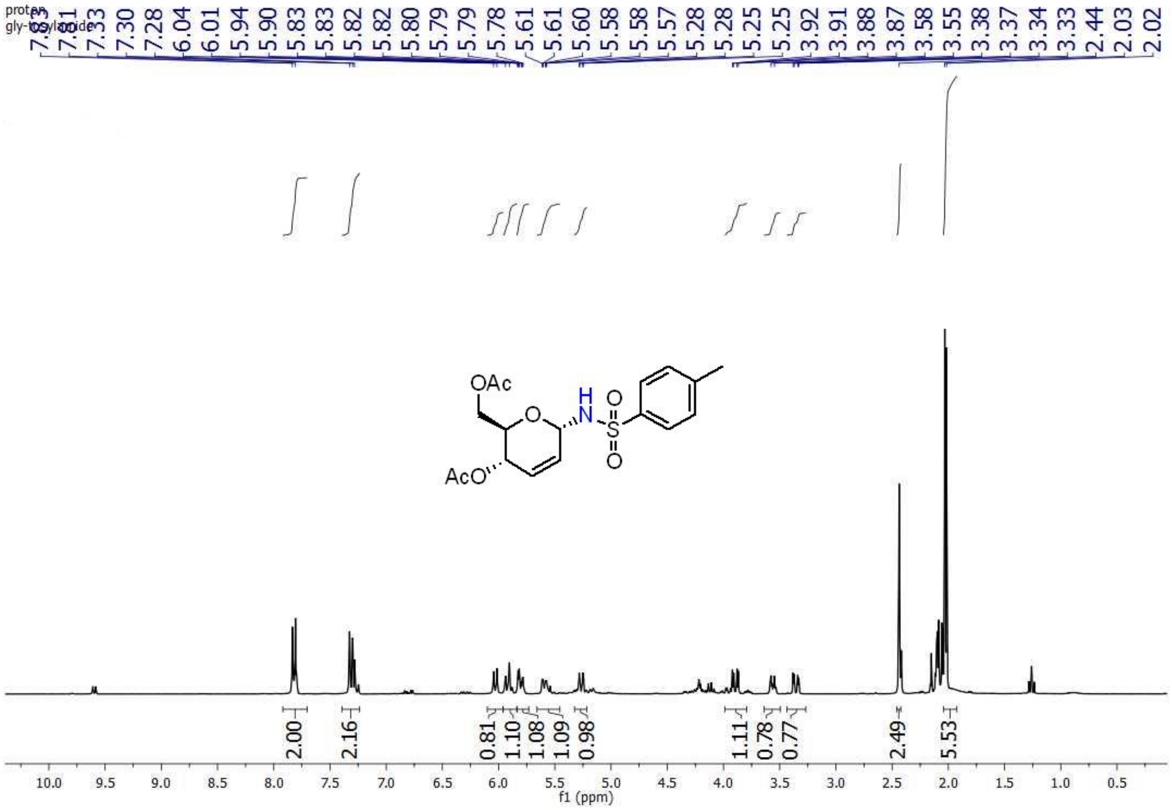

^{13}C NMR of compound 3u

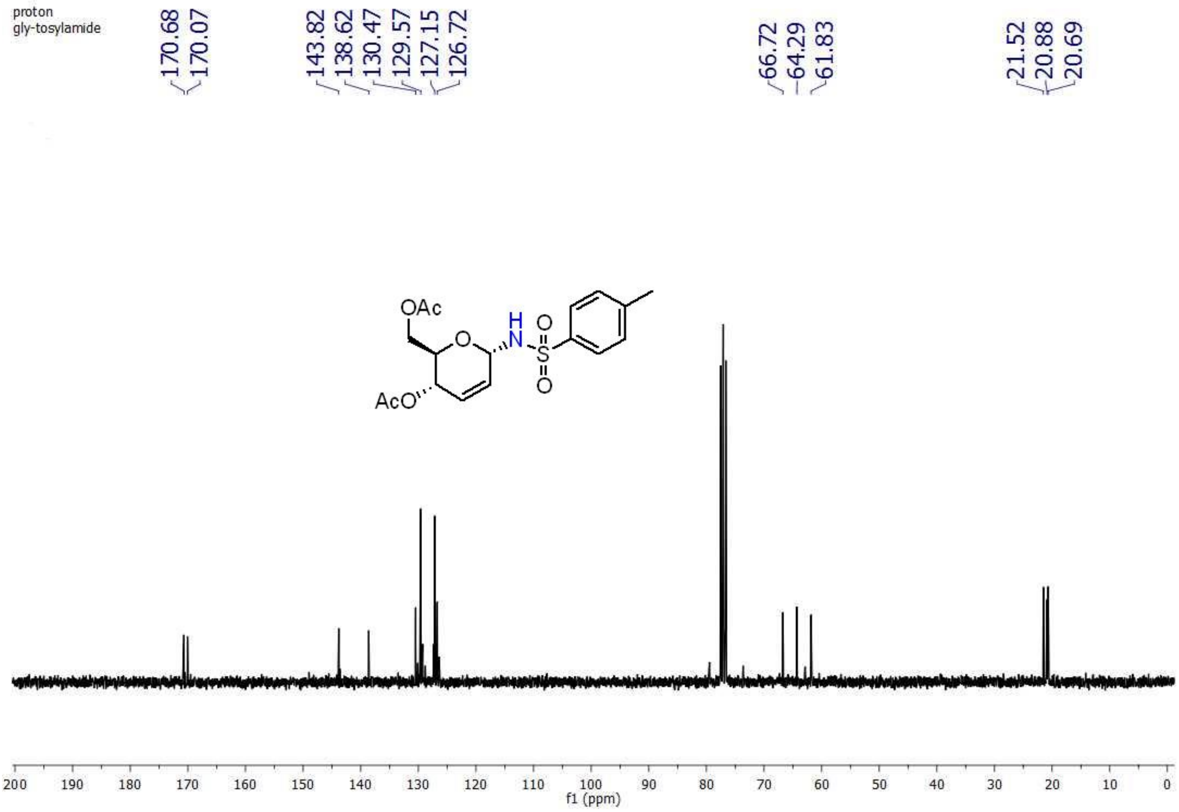

^{1}H NMR of compound 5a

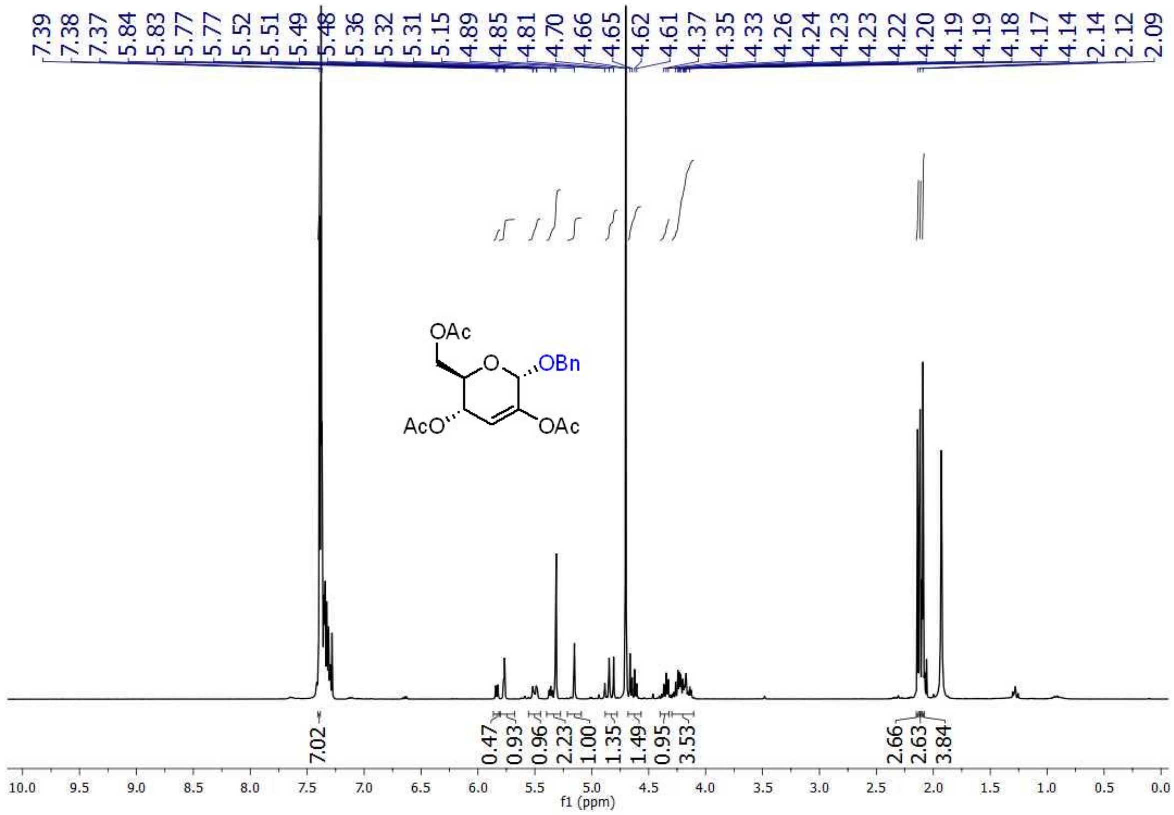

^{13}C NMR of compound 5a

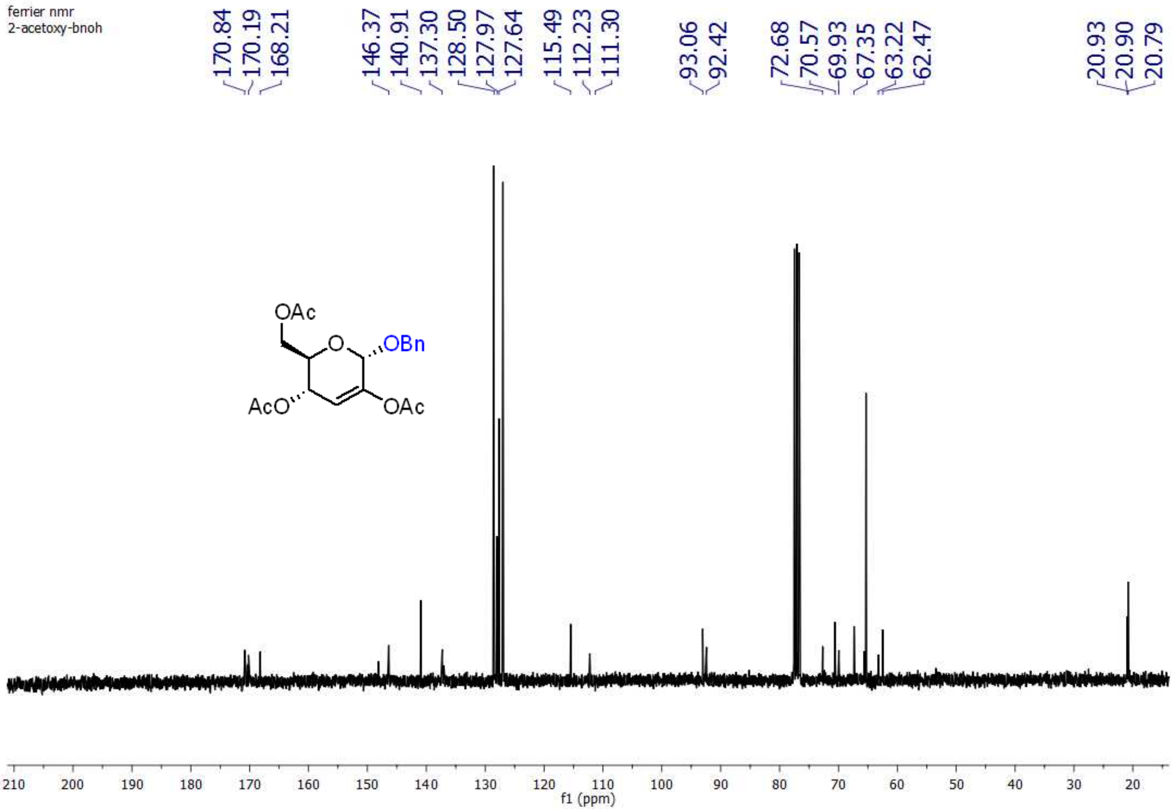

^{1}H NMR of compound 5b

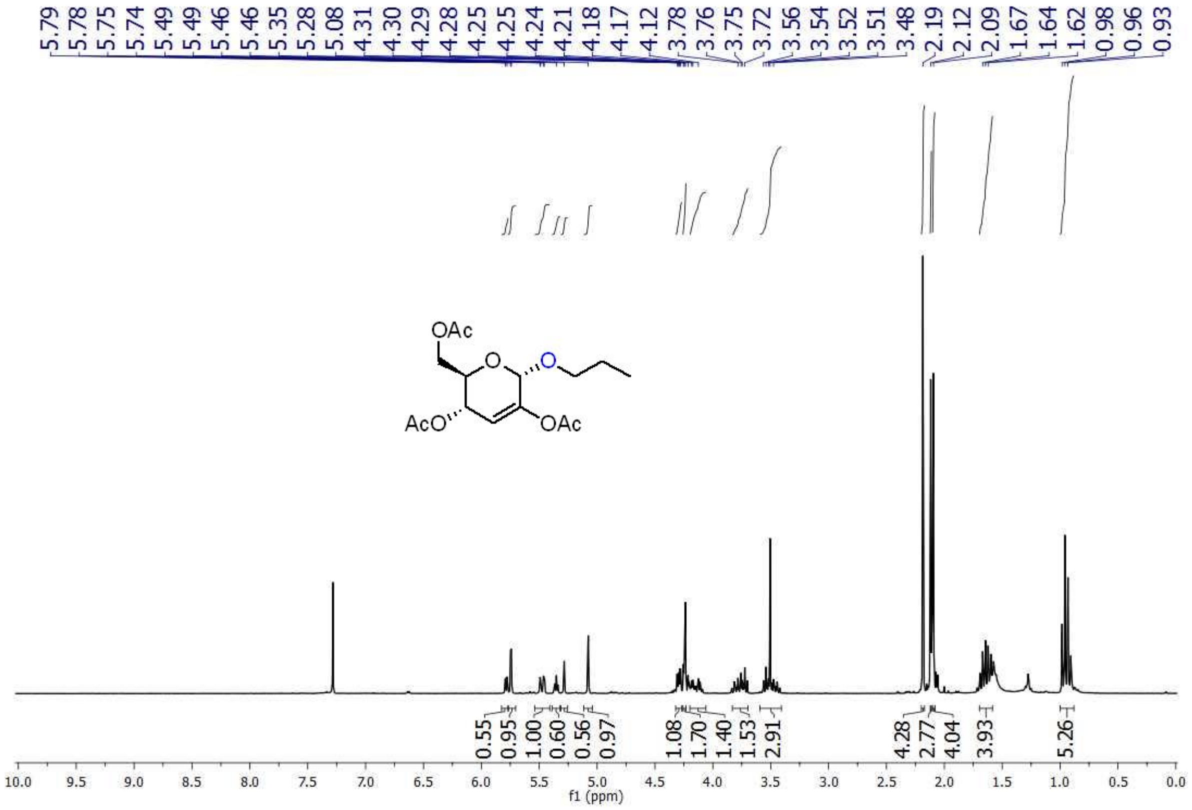

^{1}H NMR of compound 5c

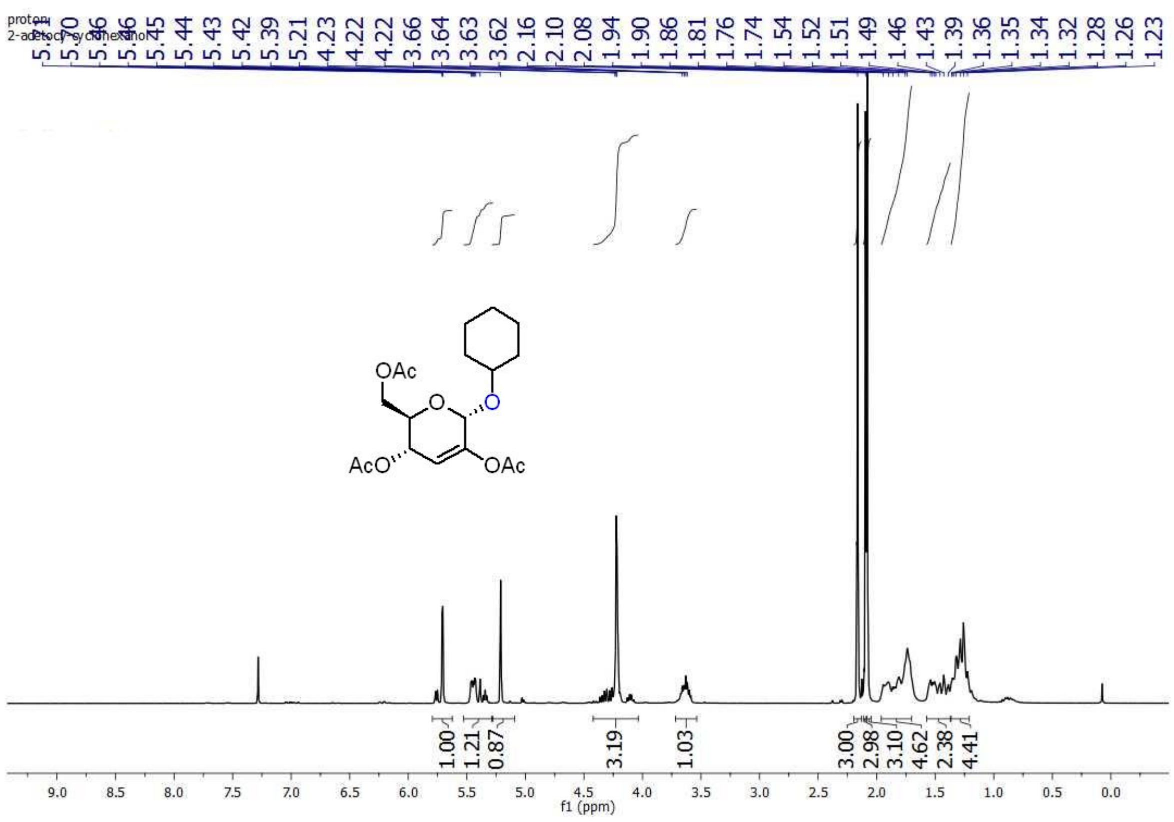

^{13}C NMR of compound 5c

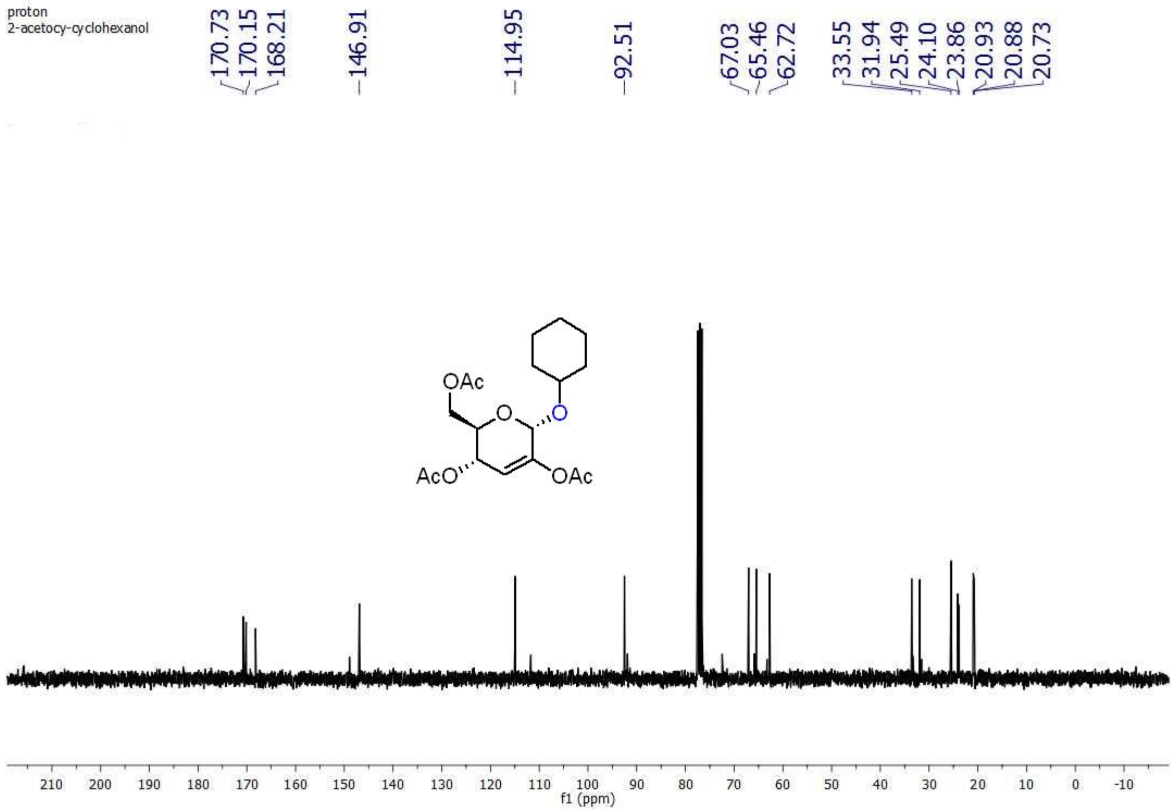

^{1}H NMR of compound 5d

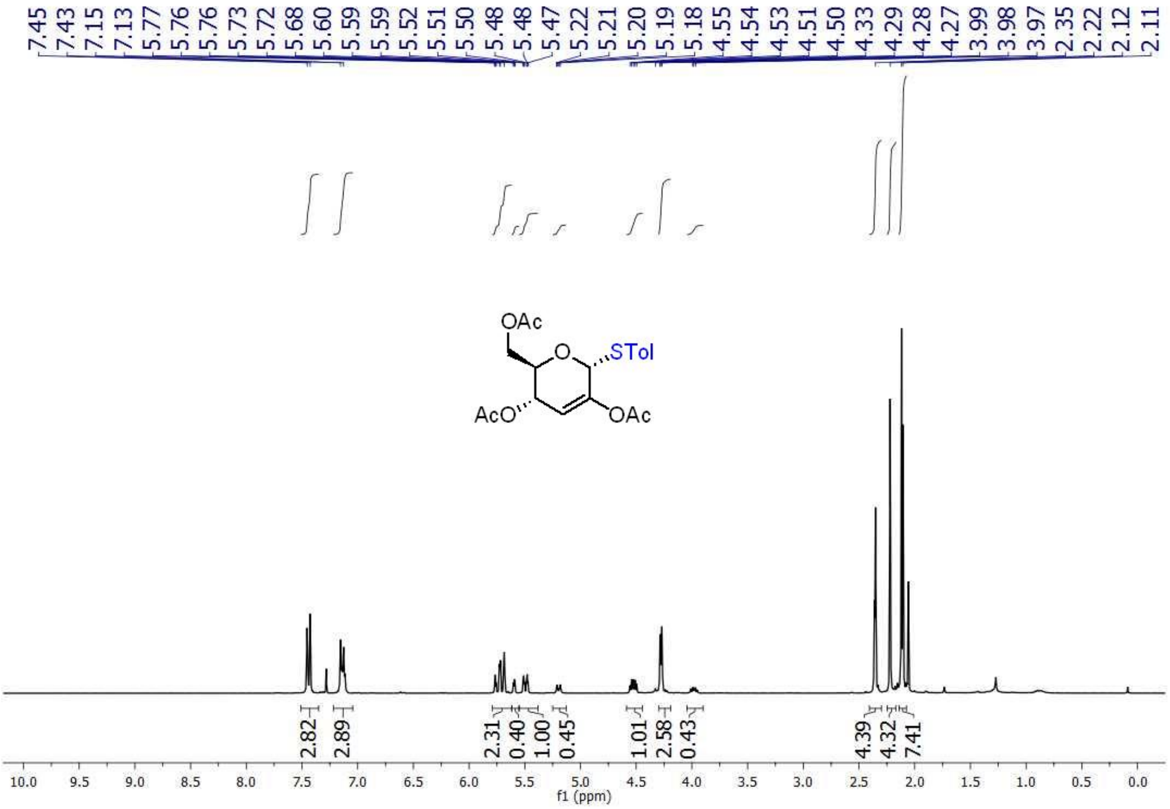

^{13}C NMR of compound 5d

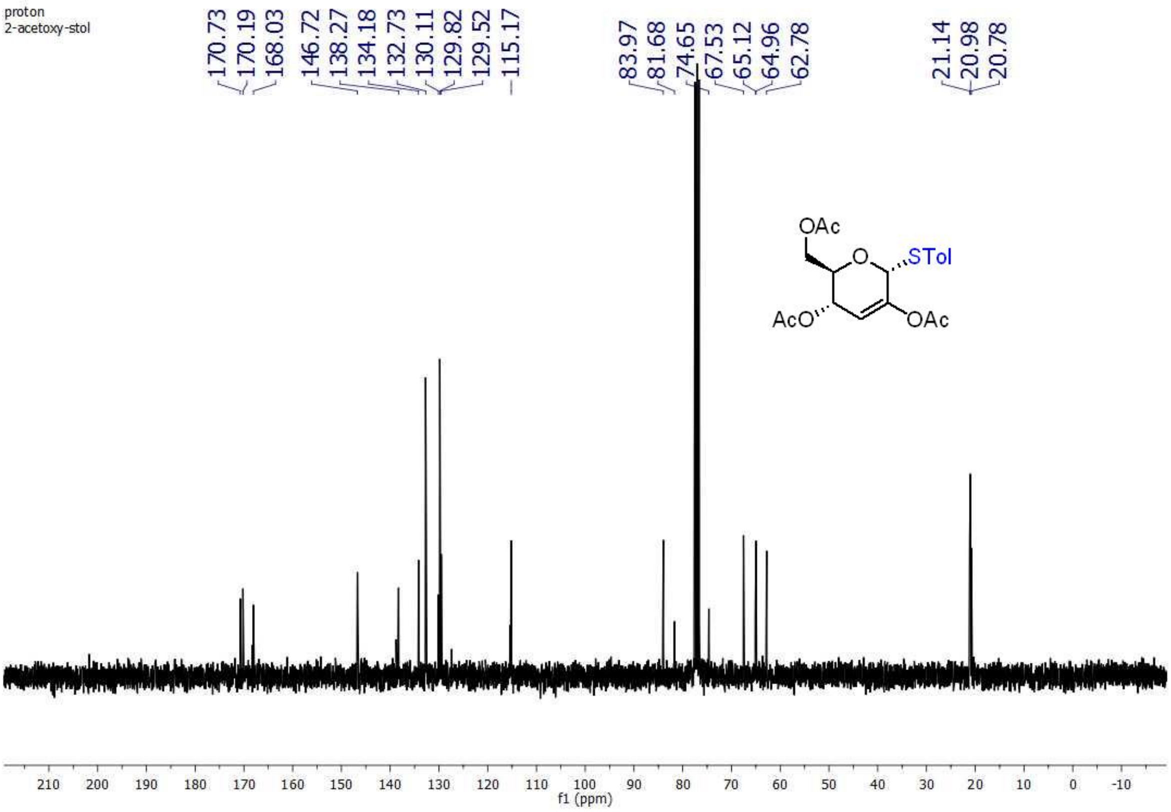

^{1}H NMR of compound 7a

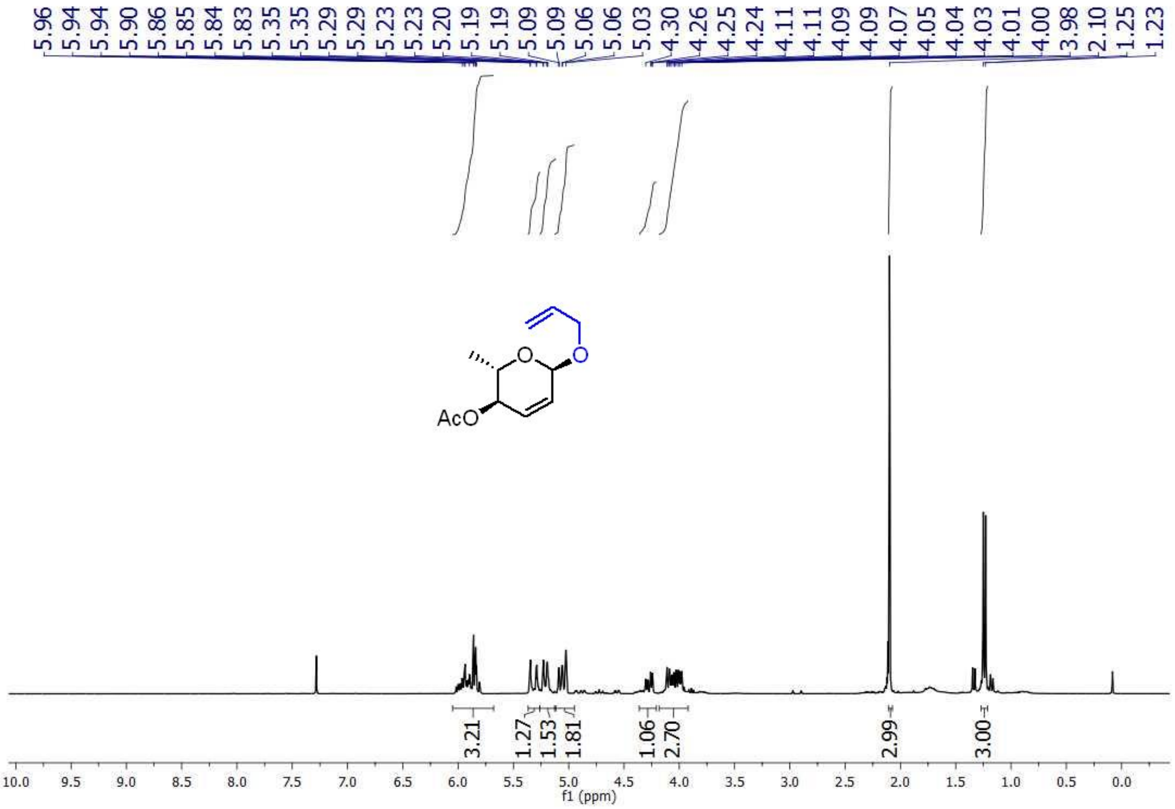

^{13}C NMR of compound 7a

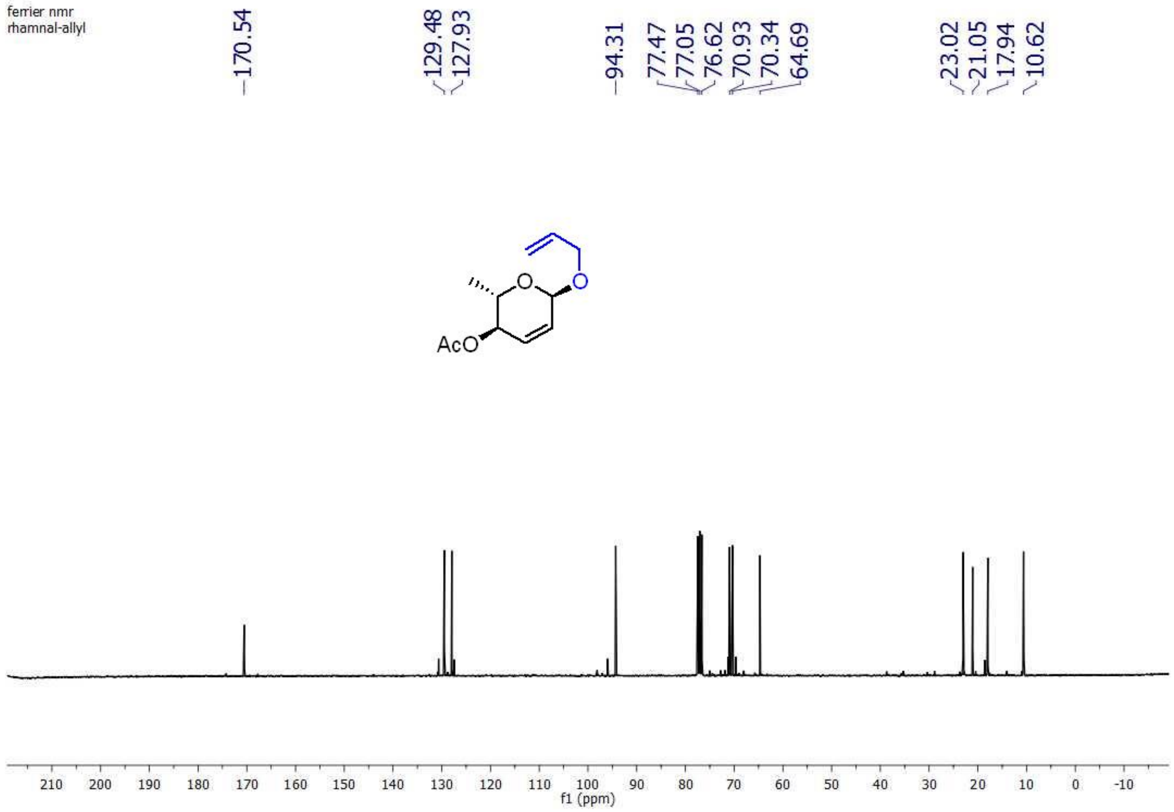

^{1}H NMR of compound 7b

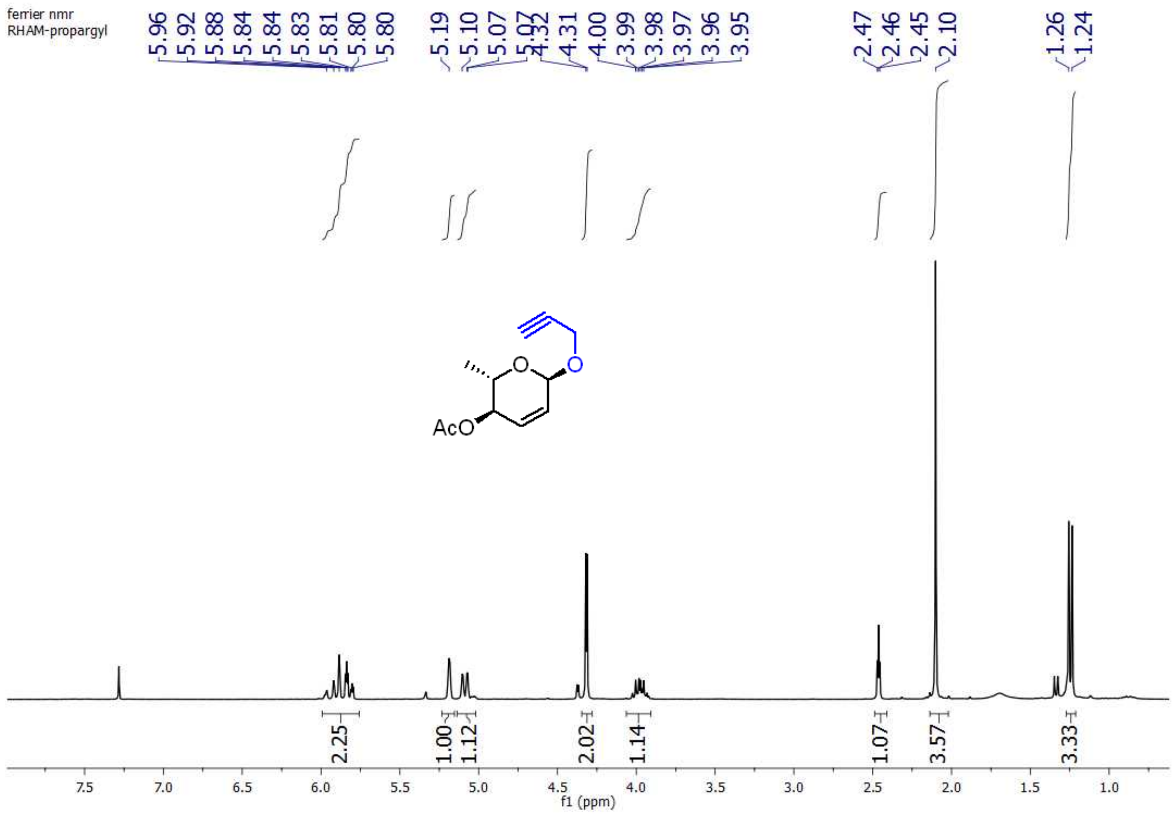

^{1}H NMR of compound 7c

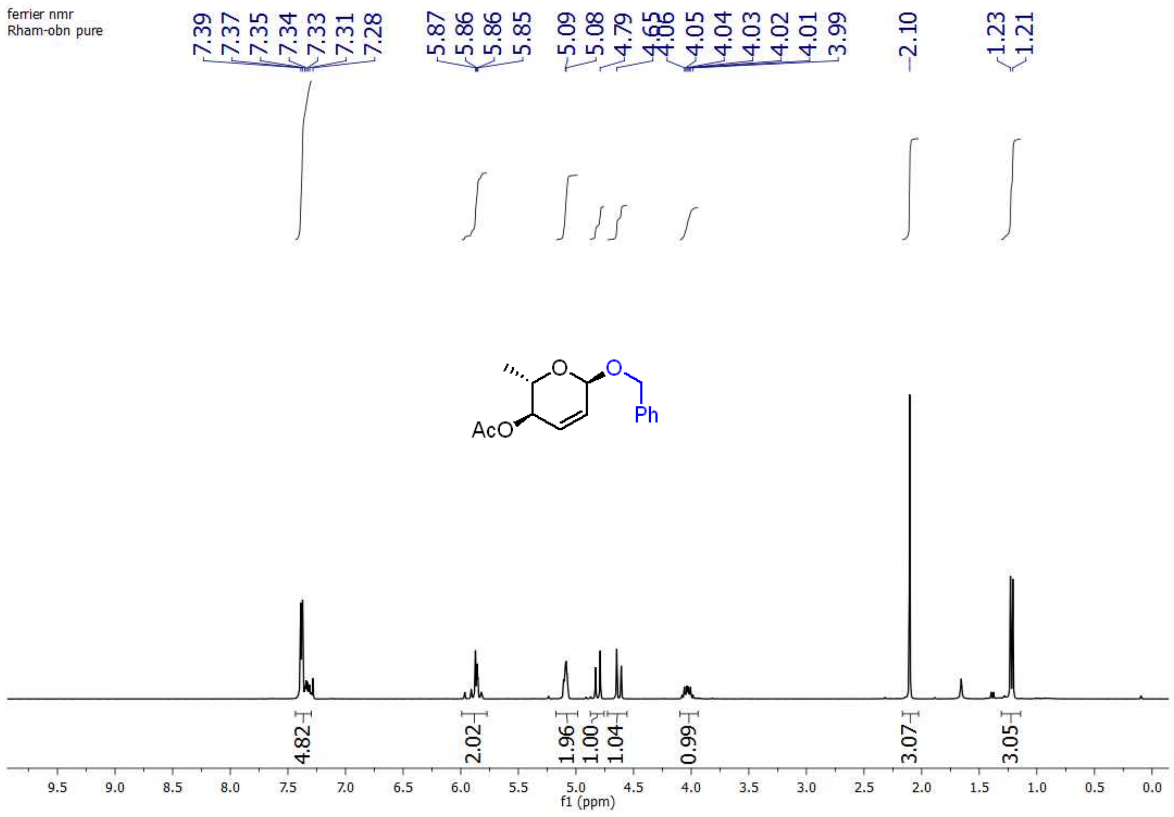

^{13}C NMR of compound 7c

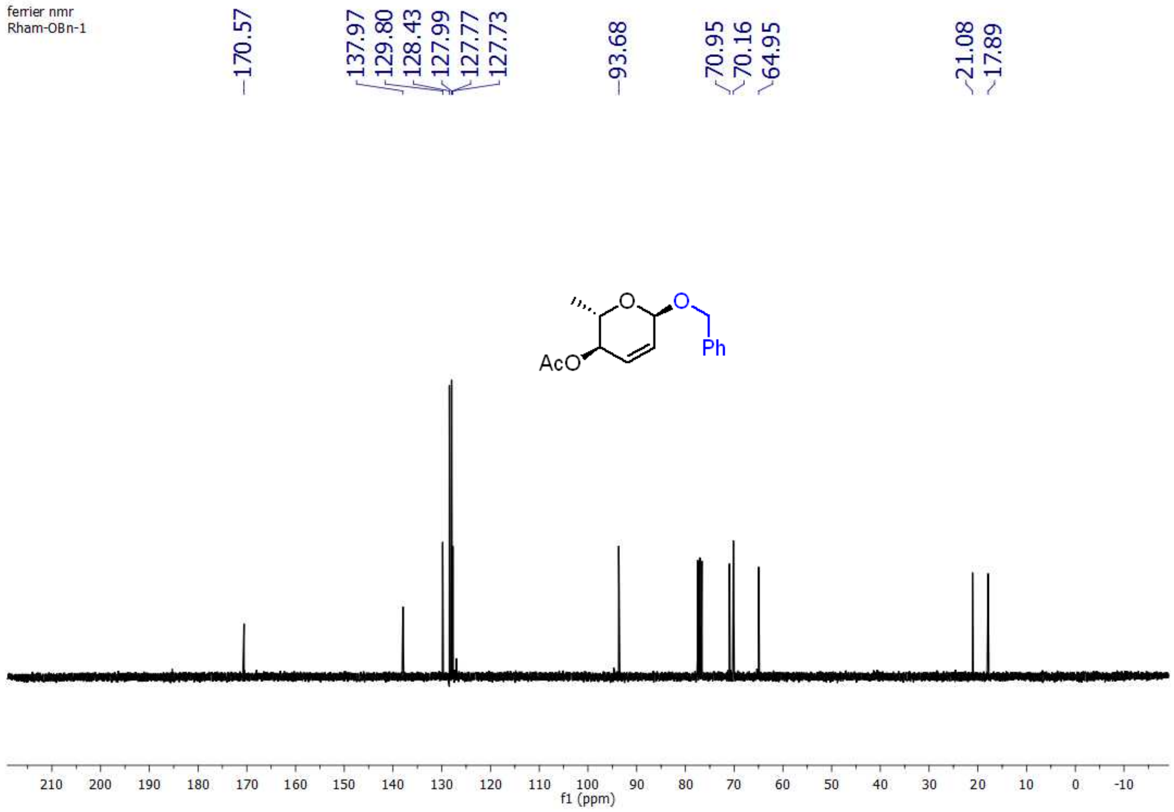

^{1}H NMR of compound 7d

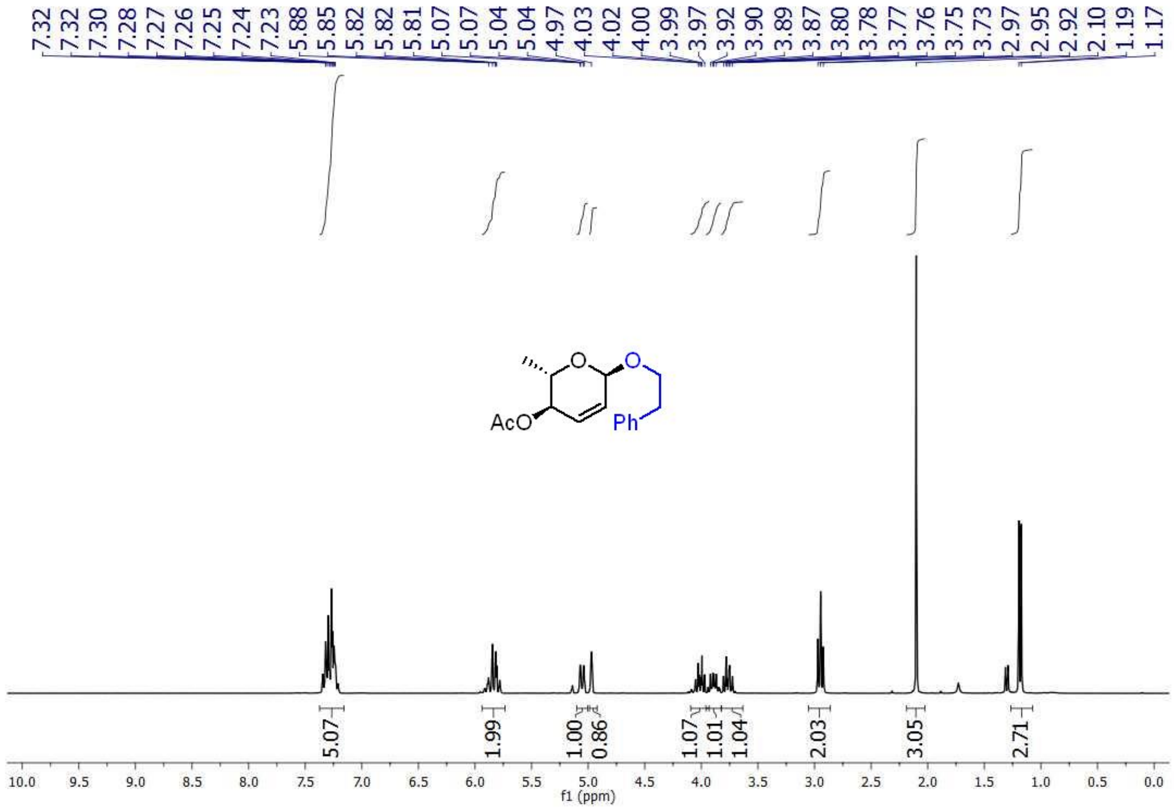

^{1}H NMR of compound 7e

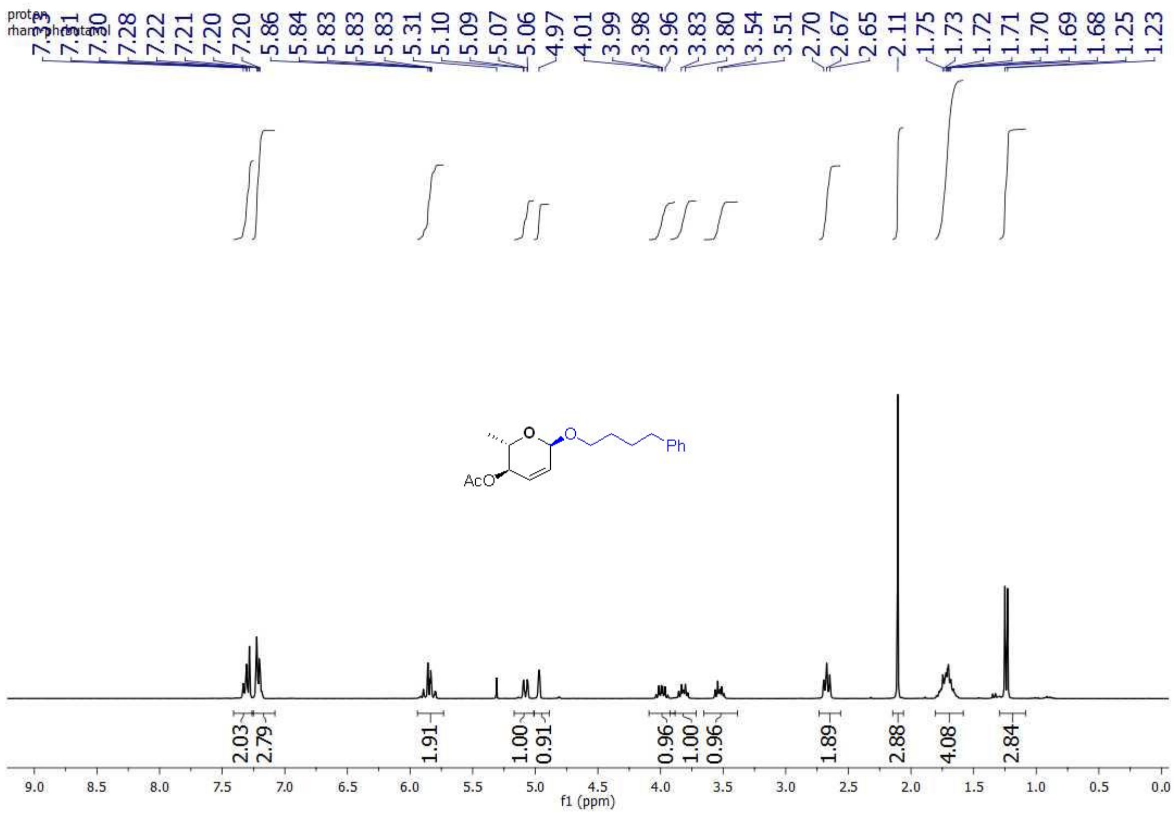

^{13}C NMR of compound 7e

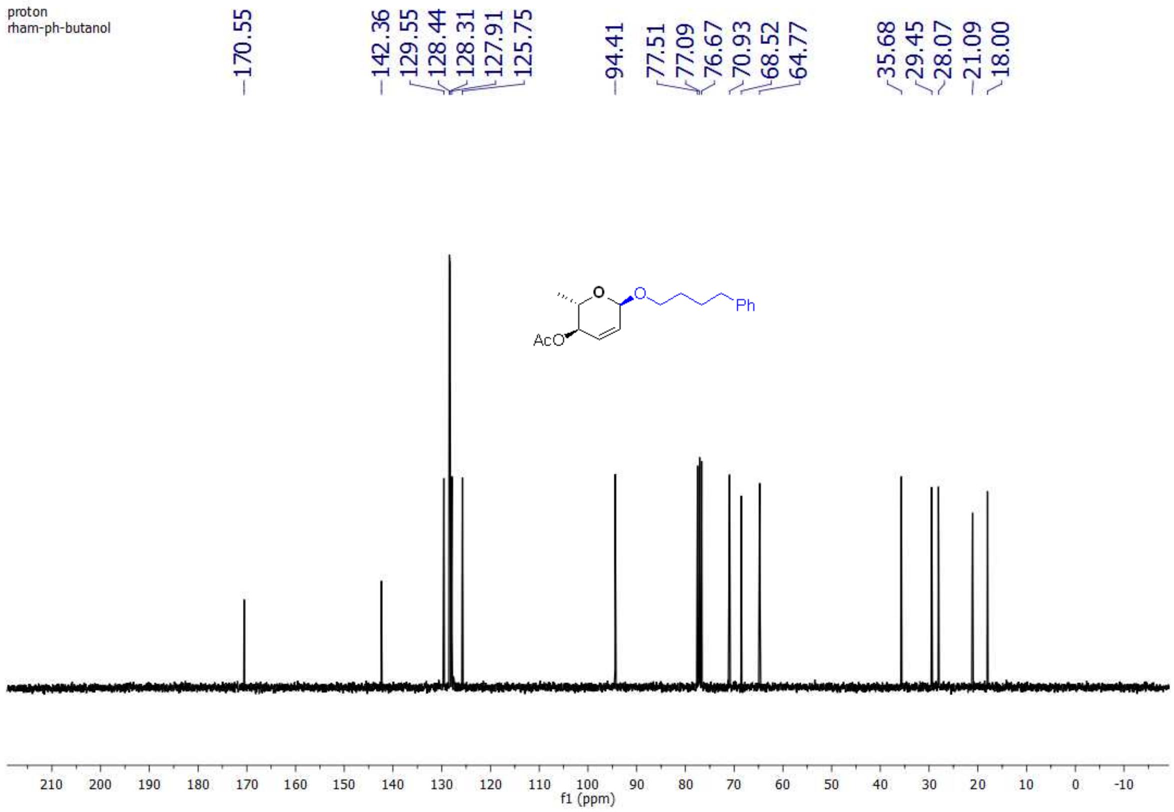

^{1}H NMR of compound 7f

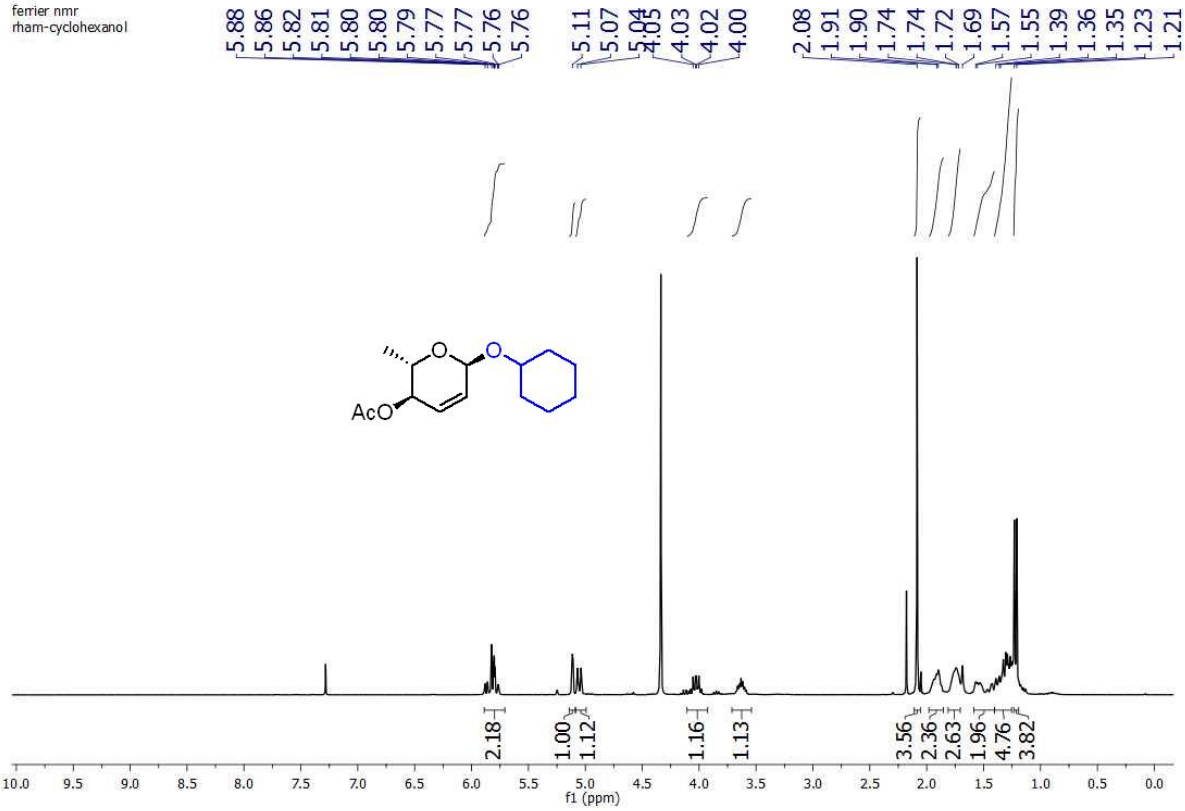

^{13}C NMR of compound 7f

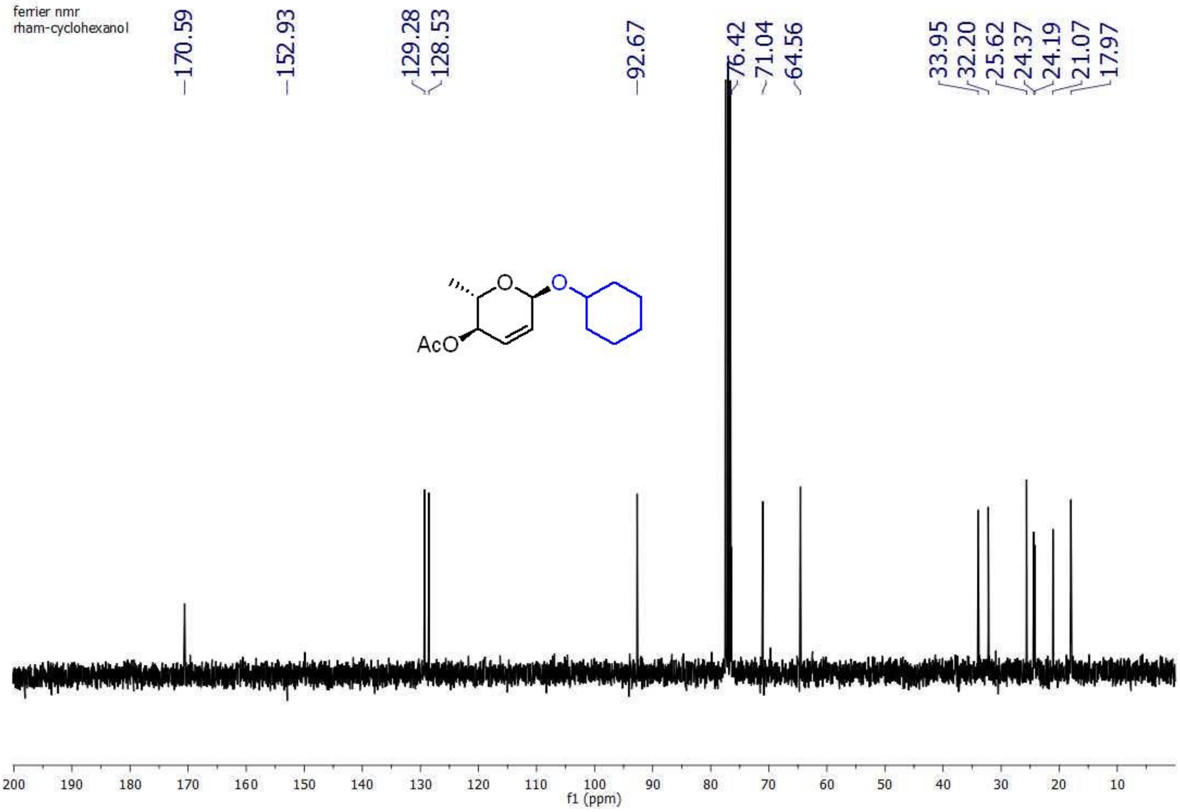

^{1}H NMR of compound 7g

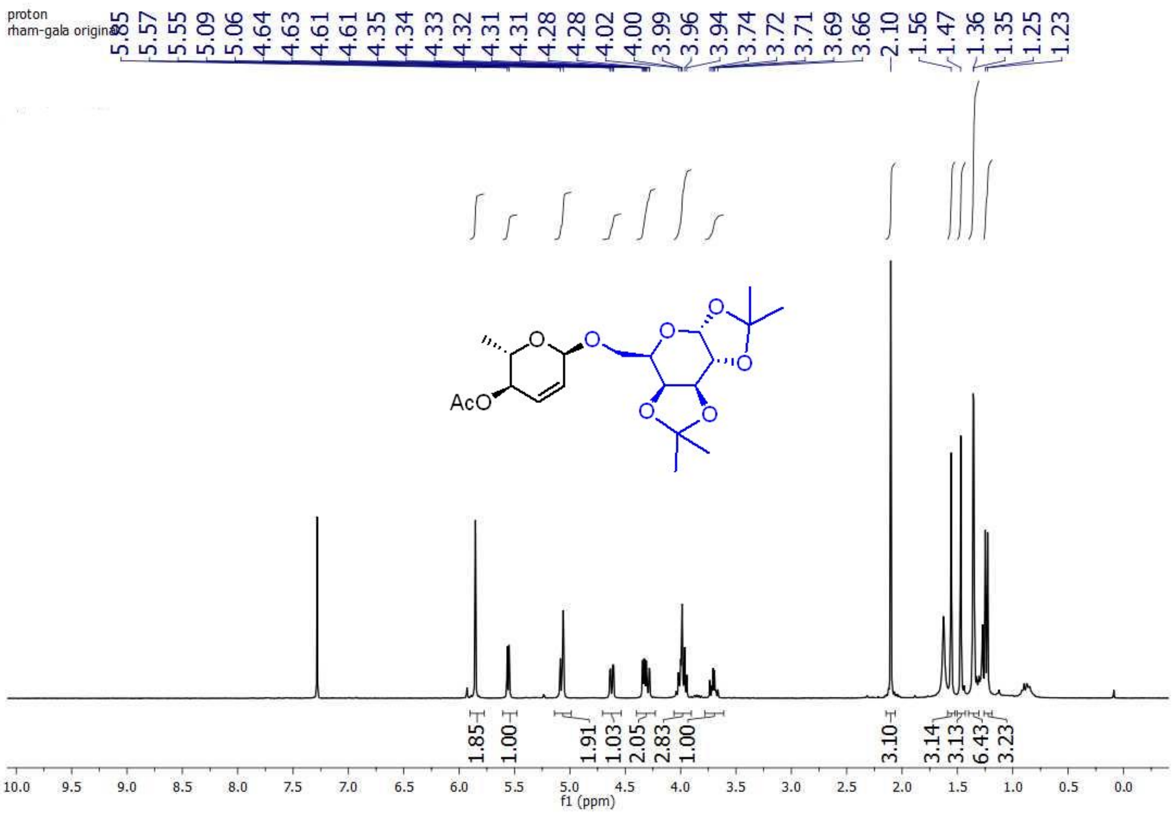

^{13}C NMR of compound 7g

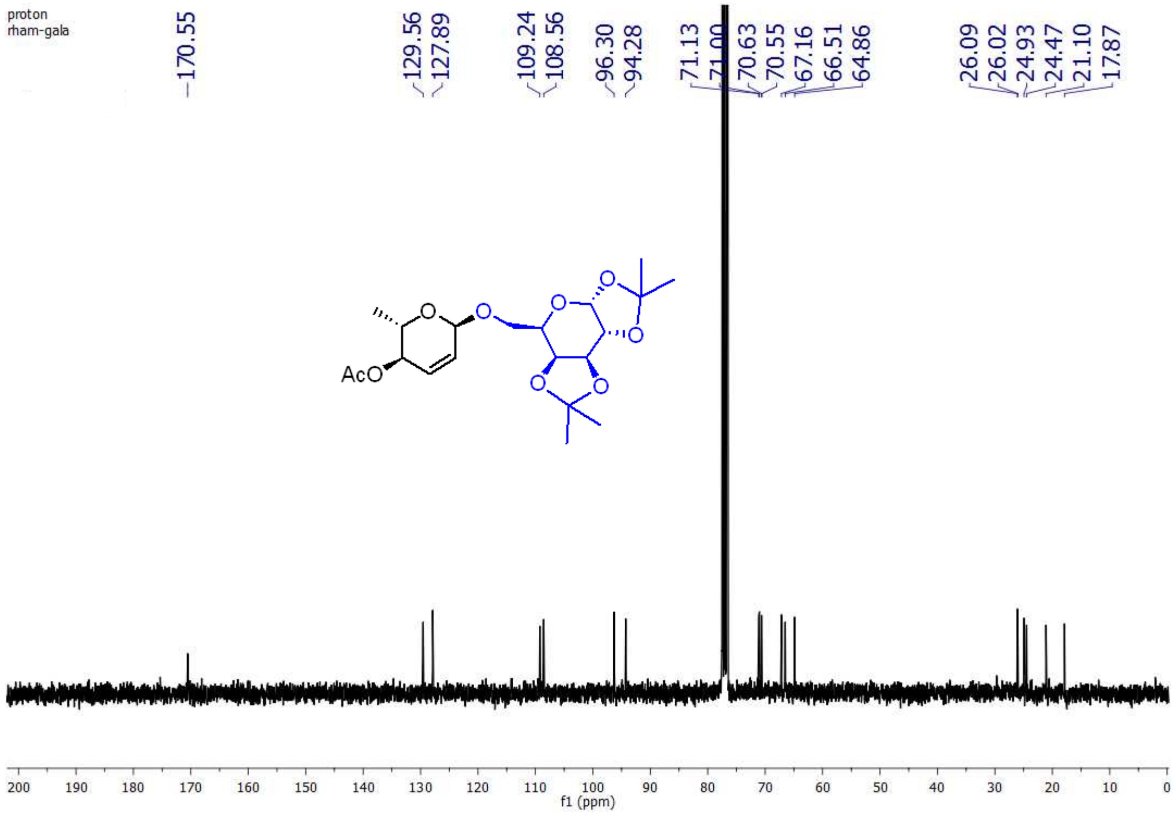

^{1}H NMR of compound 7h

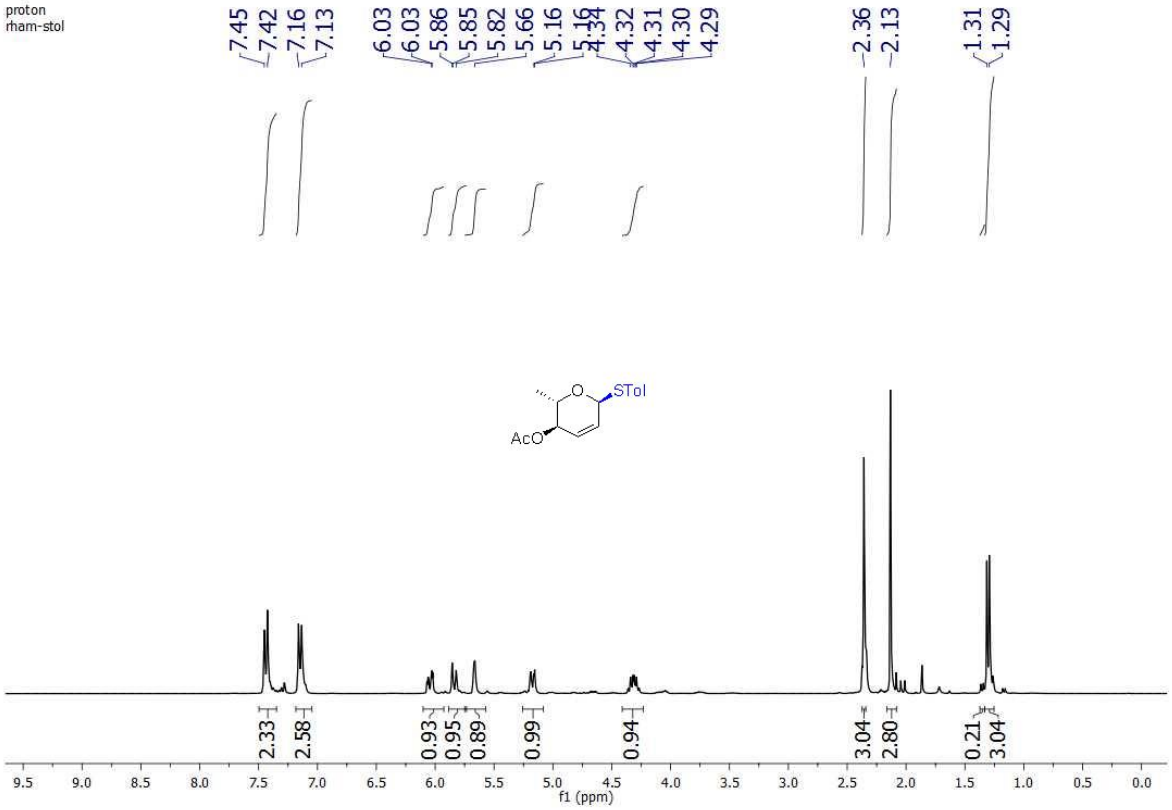

^{13}C NMR of compound 7h

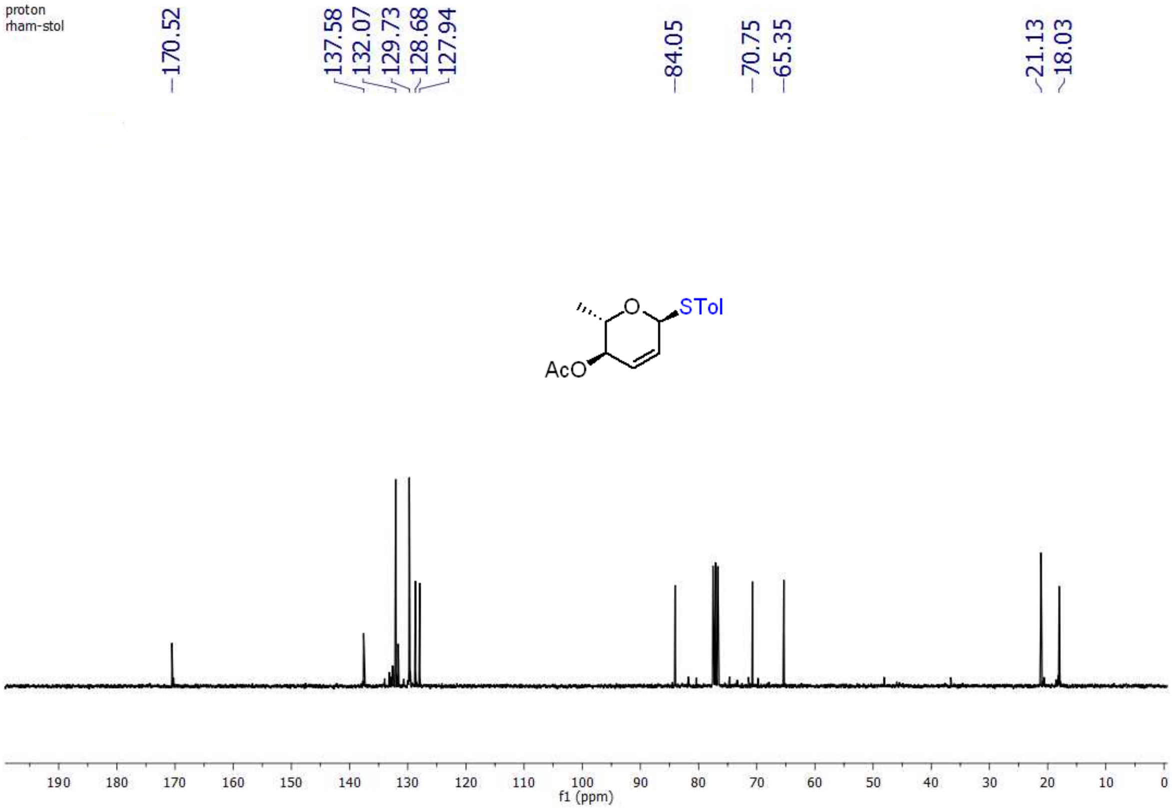

Supplement: File 1 — Experimental data and copies of 1H and 13C NMR spectra of glycosides 3a–u, 5a–d and 7a–h are provided. [file Beilstein_J_Org_Chem-15-1275-s001.pdf]
